# Detection of Synergistic Interaction on an Additive Scale Between Two Drugs on Abnormal Elevation of Serum Alanine Aminotransferase Using Machine-Learning Algorithms

**DOI:** 10.3389/fphar.2022.910205

**Published:** 2022-07-06

**Authors:** Hayato Akimoto, Takuya Nagashima, Kimino Minagawa, Takashi Hayakawa, Yasuo Takahashi, Satoshi Asai

**Affiliations:** ^1^ Division of Pharmacology, Department of Biomedical Sciences, Nihon University School of Medicine, Tokyo, Japan; ^2^ Division of Genomic Epidemiology and Clinical Trials, Clinical Trials Research Center, Nihon University School of Medicine, Tokyo, Japan

**Keywords:** machine-learning (ML) algorithms, liver function, drug induced liver injury (DILI), alanine aminotransferase, drug interaction, relative excess risk due to interaction (RERI), synergistic effect

## Abstract

Drug-induced liver injury (DILI) is a common adverse drug reaction, with abnormal elevation of serum alanine aminotransferase (ALT). Several clinical studies have investigated whether a combination of two drugs alters the reporting frequency of DILI using traditional statistical methods such as multiple logistic regression (MLR), but this model may over-fit the data. This study aimed to detect a synergistic interaction between two drugs on the risk of abnormal elevation of serum ALT in Japanese adult patients using three machine-learning algorithms: MLR, logistic least absolute shrinkage and selection operator (LASSO) regression, and extreme gradient boosting (XGBoost) algorithms. A total of 58,413 patients were extracted from Nihon University School of Medicine’s Clinical Data Warehouse and assigned to case (*N* = 4,152) and control (*N* = 54,261) groups. The MLR model over-fitted a training set. In the logistic LASSO regression model, three combinations showed relative excess risk due to interaction (RERI) for abnormal elevation of serum ALT: diclofenac and famotidine (RERI 2.427, 95% bootstrap confidence interval 1.226–11.003), acetaminophen and ambroxol (0.540, 0.087–4.625), and aspirin and cilostazol (0.188, 0.135–3.010). Moreover, diclofenac (adjusted odds ratio 1.319, 95% bootstrap confidence interval 1.189–2.821) and famotidine (1.643, 1.332–2.071) individually affected the risk of abnormal elevation of serum ALT. In the XGBoost model, not only the individual effects of diclofenac (feature importance 0.004) and famotidine (0.016), but also the interaction term (0.004) was included in important predictors. Although further study is needed, the combination of diclofenac and famotidine appears to increase the risk of abnormal elevation of serum ALT in the real world.

## Introduction

Drug-induced liver injury (DILI) is a common adverse drug reaction and a cause of acute liver failure (Senior, 2007; Lee, 2013). Various clinical studies have been conducted worldwide to assess the effect of individual drugs and therapeutic drug classes on the risk of DILI (Sgro et al., 2002; Björnsson and Olsson, 2005; Takikawa et al., 2009; Chalasani et al., 2015; Akimoto et al., 2021). In particular, several retrospective studies regarded patients who met Hy’s law as cases of hepatocellular DILI when assessing acute liver injury caused by potential hepatotoxic drugs (Robles-Diaz et al., 2014; Uetake et al., 2018; Shen et al., 2019). Hy’s law cases are generally defined as patients who experienced increased serum alanine aminotransferase (ALT) >3 × the upper limit of normal (ULN) and total bilirubin (TBL) >2 × ULN. However, Hy’s law cases are very rare events, and all drugs that can cause Hy’s law cases more frequently cause elevation of serum ALT >3 × ULN without appreciable elevation of TBL (Church and Watkins, 2018). For this reason, abnormal elevation of serum TBL is less likely to detect modest DILI. It is known that aspartate aminotransferase (AST) is less liver-specific than ALT, and alkaline phosphatase (ALP) is an indicator of cholestasis (Pratt and Kaplan, 2000). An isolated increase in serum AST or ALP is considered only as a biochemical abnormality rather than a sign of liver injury (Teschke et al., 2013). Thus, it is important to test for abnormal elevation of ALT when evaluating potential risk factors for modest-to-severe DILI.

In 2004, it was reported that using two or more potential hepatotoxic drugs increases the risk of acute liver failure by a factor of 6 compared to using one in the UK population (de Abajo et al., 2004). Following this study, several studies have investigated whether there are combinations of two drugs that alter the reporting frequency of DILI using a multiple logistic regression (MLR) model, which is one of the traditional statistical models (Suzuki et al., 2009; Suzuki et al., 2015; Yazici et al., 2015). Although a MLR model can evaluate associations between drugs and the risk of DILI with adjustment for covariates, the generated regression model may over-fit the data (Kim et al., 2018). Machine learning is an alternative analytical approach that can handle complex relationships between variables in very large data sets. Machine-learning algorithms have been used to predict DILI and are superior to traditional statistical models because they handle non-linearity and complex feature interaction (Cruz-Monteagudo et al., 2008; Ekins et al., 2010; Rodgers et al., 2010; Liu et al., 2011; Low et al., 2011; Zhu et al., 2014). However, few studies have investigated the associations between a combination of two drugs and the risk of DILI. Hence, the aim of this study was to investigate whether there is a combination of two drugs that has combined effects on the risk of abnormal elevation of serum ALT using machine-learning algorithms.

## Materials and Methods

### Data Source

The present study was a population-based case-control study utilizing electronic medical records from the Nihon University School of Medicine’s Clinical Data Warehouse (NUSM’s CDW) between April 1, 2004 and July 1, 2021. NUSM’s CDW is a centralized data repository that integrates separate databases, including patient demographics, diagnoses, and laboratory data, from the hospital information systems at three hospitals affiliated with the NUSM; Nihon University Itabashi Hospital, Nerima Hikarigaoka Hospital, and Surugadai Nihon University Hospital. To protect patient privacy, patient identifiers are replaced by anonymous identifiers in all databases of the CDW.

### Study Subjects and Binary Outcome

First, 122,285 Japanese patients who underwent liver function tests at least three times within 90 days and had serum ALT values within the normal range [4.0–44.0 units per liter (U/L)] on the first and second measurement days were extracted from NUSM’s CDW. The latest measurement day was regarded as the index date. Among these patients, those whose ALT value reached >3 × ULN [>132.0 U/L] on the index date were regarded as patients with abnormal elevation of serum ALT and were assigned to the case group (outcome = 1; *N* = 14,634). On the other hand, those whose ALT value was within the normal range on all measurement days including the index date were assigned to the control group (outcome = 0; *N* = 80,484). Patients who had an ALT value between >44.0 and ≤132.0 U/L on the index date were excluded because mild-to-moderate elevation of serum ALT also occurs in patients with other diseases including dyslipidemia, diabetes and metabolic syndrome (Clark et al., 2003; Liu et al., 2014). Next, among the patients in the case and control groups, patients who met the following exclusion criteria were excluded. The remaining 58,413 patients (the case group *N* = 4,152; the control group *N* = 54,261) were included in the development and testing of a prediction model derived from machine-learning algorithms (Supplementary Figure S1).

### Excluded Patients

1) Under 18 years old
2) Missing any of four liver function test values on both the first measurement day and index date: serum AST, TBL, ALP and ALT
3) Pre-existing liver disease (International classification of disease 10 [ICD-10] codes are shown in Supplementary Table S1): infectious hepatitis such as viral hepatitis, alcoholic liver disease, nonalcoholic fatty acid disease, malignant neoplasm of liver, and other causes
4) Taken any hepatoprotectants: glycyrrhizic acid (Anatomical Therapeutic Chemical [ATC] fifth level: A05BA08), glutathione (V03AB32), lactulose (A06AD11), L-arginine glutamate (A05BA01), tiopronin (G04BX16), lactitol (A06AD12), rifaximin (A07AA11), ursodeoxycholic acid (A05AA02), liver hydrolysate (not applicable), or branched chain amino acid preparations (not applicable)

### Features

We obtained 1,375 features in the eligible patients: six demographic characteristics, medical history which included five diagnoses, use or non-use of 180 different drugs, 1,050 product terms of these drugs, and presence or absence of 134 different therapeutic classes. The demographic information was composed of age, sex, three hospitals (Itabashi, Hikarigaoka, and Surugadai, dummy variables), and the number of concomitant drugs. The number of concomitant drugs was the sum of drugs that were continued to be used at least until the index date irrespective of the start date. The medical history was composed of hypertension (ICD-10 codes; I10), diabetes (E10.X, E11.X, and E14.X), dyslipidemia (E78.0-E78.5), heart failure (I50.0, I50.1, and I50.9), and sepsis (A40.X and A41.X), which are known as risk factors for acute liver injury.

To detect departure from additivity in the association between a combination of two drugs and the risk of abnormal elevation of ALT, it is necessary to include a product term of the two drugs as well as the effects of the individual drugs in a dataset for machine-learning. In the eligible patients, 1,089 different drugs were newly started within 90 days before the index date and continued to be used at least until the index date. Continuation of drug use until the index date means that there is a prescription date before and after the index date (drugs A and B in Supplementary Figure S2), or date that added days of supply to the latest prescription date exceeds the index date (drugs C and D in Supplementary Figure S2). Among these drugs, 180 different drugs were used in combination with each other in ≥100 patients until the index date, and the number of combinations was 1,050. Use and non-use of the 180 different drugs were treated as features. Regarding the combinations, 1,050 product terms (i.e., 1,050 two-way interaction terms) between the 180 drugs were calculated and treated as features. When the included product term is 1, it means that the two drugs were used in combination on the index date, whereas when it is 0, it means that one of the two drugs was used or neither of them was used on the index date. That is, the present study evaluated whether the 1,050 combinations of the 180 drugs had a synergistic interaction on the risk of abnormal elevation of ALT.

Of the 1,089 newly started drugs, the remaining 909 drugs were used until the index date, but were not frequently used in combination with each other (<100 patients). Since approximately 1 in 100 patients develops DILI during hospitalization (Meier et al., 2005), it is difficult to evaluate the association between these infrequent drugs and DILI. Hence, we classified these drugs into chemical subgroups using the fourth levels of the ATC classification published by WHO Collaborating Centre for Drug Statistics Methodology, and then the chemical subgroups were assigned to 134 therapeutic classes (Supplementary Table S2). The presence and absence of these 134 therapeutic classes were treated as features. Finally, a two-dimensional dataset (58,413 × 1,375) for machine-learning was generated. Data imputation was not performed because all the observations in the dataset had no missing values.

### Construction of Machine-Learning Models and Model Evaluations

The dataset for machine-learning was randomly split into a training set for the development of algorithms (70%; *N* = 40,889) and a testing set for evaluation (30%; *N* = 17,524). To evaluate the effects of individual drugs and their product terms on the risk of abnormal elevation of serum ALT, three machine-learning approaches were utilized in this study: MLR model and logistic least absolute shrinkage and selection operator (LASSO) regression model, which are linear algorithms, and extreme gradient boosting (XGBoost) tree model, which is a tree-based algorithm. All machine-learning approaches were performed using R software (version 4.0.4; R Foundation for Statistical Computing, Vienna, Austria).

MLR was performed with the log odds for abnormal elevation of ALT as a binary dependent variable. The independent variables consisted of the 1,375 features; that is, the 1,372 features excluding the individual effects of the two drugs of interest and their product terms were regarded as covariates. The same dataset was also used in the logistic LASSO regression and XGBoost models. When constructing the logistic LASSO regression model, we ran 10-fold cross-validation to determine a lambda (λ) minimizing the misclassification error rate for the training set and to avoid over-fitting to the training set using R “glmnet” package. The LASSO regularized regression equation was obtained using the optimized λ value (Supplementary Figure S3). The XGBoost model was constructed using R “xgboost” package. The hyperparameters of the XGBoost model are roughly divided into the following four parameters: general, booster, learning task, and command line parameters. Of these parameters, booster parameters were optimized by grid search. Finally, the XGBoost model with the optimized hyperparameters was constructed (Supplementary Table S3).

To assess the prediction performance of each algorithm, area under the receiver operating characteristic curve (AUROC; C statistic) was calculated and compared between the three algorithms using the DeLong Test. However, the dataset was strongly imbalanced since the ratio of patients in the control and case groups was approximately 93:7. When doing machine-learning with such an imbalanced dataset, it is important to evaluate the proportion of true positive cases among positive predictions (Saito and Rehmsmeier, 2015). Thus, sensitivity (recall), positive predictive value (PPV, precision), specificity, negative predictive value (NPV), F1-score, and area under the precision-recall curve (AUPR) were also calculated. R “pROC” and “PRROC” packages were used to calculate these metrics.

### Detection of Synergistic Interaction on an Additive Scale Between Two Drugs for Risk of Abnormal Elevation of ALT

The adjusted odds ratio (aOR) and corresponding 95% confidence interval (95%CI), which represent the association of a feature with the risk of an outcome, can be calculated from regression coefficients and their standard errors in the MLR model. However, as the MLR model over-fitted the training set, detection of synergistic interaction was not performed in the MLR model. In the logistic LASSO regression model, a point estimate for each feature is calculated, but its SE is not. Thus, 95%CI for aOR was estimated with a bootstrap percentile method (Jung et al., 2019). To obtain 95% bootstrap percentile CI (95%BootCI), 2,000 bootstrap samples, each of which was the same size as the training set, were generated by resampling with replacement from the training set. After a parameter estimate was calculated from each bootstrap sample, 2,000 parameter estimates in all the bootstrap samples were sorted in ascending order. The interval between the 50th and 1950th quantile values of the 2,000 parameter estimates was regarded as the 95%BootCI.

Relative excess risk due to interaction (RERI) has been used to detect whether there are combined effects of two exposures on an outcome. RERI can be calculated by substituting the regression coefficients from the logistic LASSO regression model into the following formulae (1) and (2) (Knol et al., 2007). A RERI of 0 indicates no interaction on an additive scale. $\hat{\beta_{1}}$ , $\hat{\beta_{2}}$ , and $\hat{\beta_{3}}$ represent regression coefficients for drug 1, drug 2, and a product term of drugs 1 and 2, respectively. 95%CI for RERI was estimated as BootCI by substituting parameter estimates in all the bootstrap samples into these formulae.

$$\left(e^{\hat{\beta_{1}}+\hat{\beta_{2}}+\hat{\beta_{3}}}-1\right)\neq \left(e^{\hat{\beta_{1}}}-1\right)+\left(e^{\hat{\beta_{2}}}-1\right)$$ (1)

and

$$\mathrm{RERI}=\;e^{\hat{\beta_{1}}+\hat{\beta_{2}}+\hat{\beta_{3}}}-e^{\hat{\beta_{1}}}-e^{\hat{\beta_{2}}}+1$$ (2)

In the present study, combinations that had a product term with a lower limit of aOR 95%BootCI >1 and a lower limit of RERI 95%BootCI >0 were considered to have a positive synergy for the risk of abnormal elevation of ALT. Furthermore, feature importance was obtained to confirm whether these combinations that have a synergistic interaction on an additive scale could also be important predictors in the XGBoost model.

### Statistical Analysis

To compare the patient characteristics between the case and control groups and between the training and testing sets, unpaired 2-tailed Welch’s *t*-test or Wilcoxon rank-sum test for continuous data and chi-squared test for categorical data were performed. The level of statistical significance was set at 5.0% for all statistical analyses. All statistical analyses were performed using R software.

## Results

### Patient Characteristics

A total of 58,413 patients were extracted from NUSM’s CDW and assigned to the case (*N* = 4,152) and control (*N* = 54,261) groups. Differences in patient characteristics are shown in Table 1. Mean age (standard deviation) in the case and control groups was 63.8 (16.6) and 57.0 (19.6) years, respectively. The male percentage and the number of concomitant drugs in the case group were higher than those in the control group. There were significant differences between the two groups in all patient characteristics (*p* < 0.001, respectively). On the other hand, patients in the case and control groups had similar characteristics between the training and testing sets (Supplementary Tables S4, S5).

**TABLE 1:** Patient characteristics in case and control groups.

Characteristics	Case group (*N* = 4,152)	Control group (*N* = 54,261)	*p* value
Age (years), mean (SD)	63.8 (16.6)	57.0 (19.6)	<0.001
Male, *n* (%)	2,639 (63.6)	21,941 (40.4)	<0.001
Number of concomitant drugs, median (IQR)	7.0 (4.0–10.0)	4.0 (2.0–6.0)	<0.001
Hospital, *n* (%)			<0.001
Itabashi	3,425 (82.5)	42,311 (78.0)	
Hikarigaoka	370 (8.9)	6,107 (11.3)	
Surugadai	357 (8.6)	5,843 (10.8)	
Medical history, *n* (%)			
Hypertension	1,203 (29.0)	9,399 (17.3)	<0.001
Diabetes	1,412 (34.0)	15,035 (27.7)	<0.001
Dyslipidemia	737 (17.8)	6,930 (12.8)	<0.001
Heart failure	871 (21.0)	5,623 (10.4)	<0.001
Sepsis	319 (7.7)	616 (1.1)	<0.001
Liver function tests, median (IQR)			
ALT (U/L)	182.0 (150.0–276.2)	14.0 (11.0–20.0)	<0.001
AST (U/L)	171.0 (105.0–342.0)	19.0 (15.0–23.0)	<0.001
TBL (mg/dl)	0.8 (0.5–1.5)	0.5 (0.4–0.7)	<0.001
ALP (U/L)	361.0 (239.0–618.0)	209.0 (165.0–264.0)	<0.001

Unpaired two-tailed Welch’s t-test was performed for differences in age. Wilcoxon rank-sum test was performed for differences in number of concomitant drugs and liver function test values. Chi-squared test was performed for differences in categorical variables such as sex, hospital, and medical history. ALP, alkaline phosphatase; ALT, alanine aminotransferase; AST, aspartate aminotransferase; IQR, interquartile range; SD, standard deviation; TBL, total bilirubin; U/L, units per liter.

### Predictive Performance of Machine-Learning Algorithms

**FIGURE 1:**
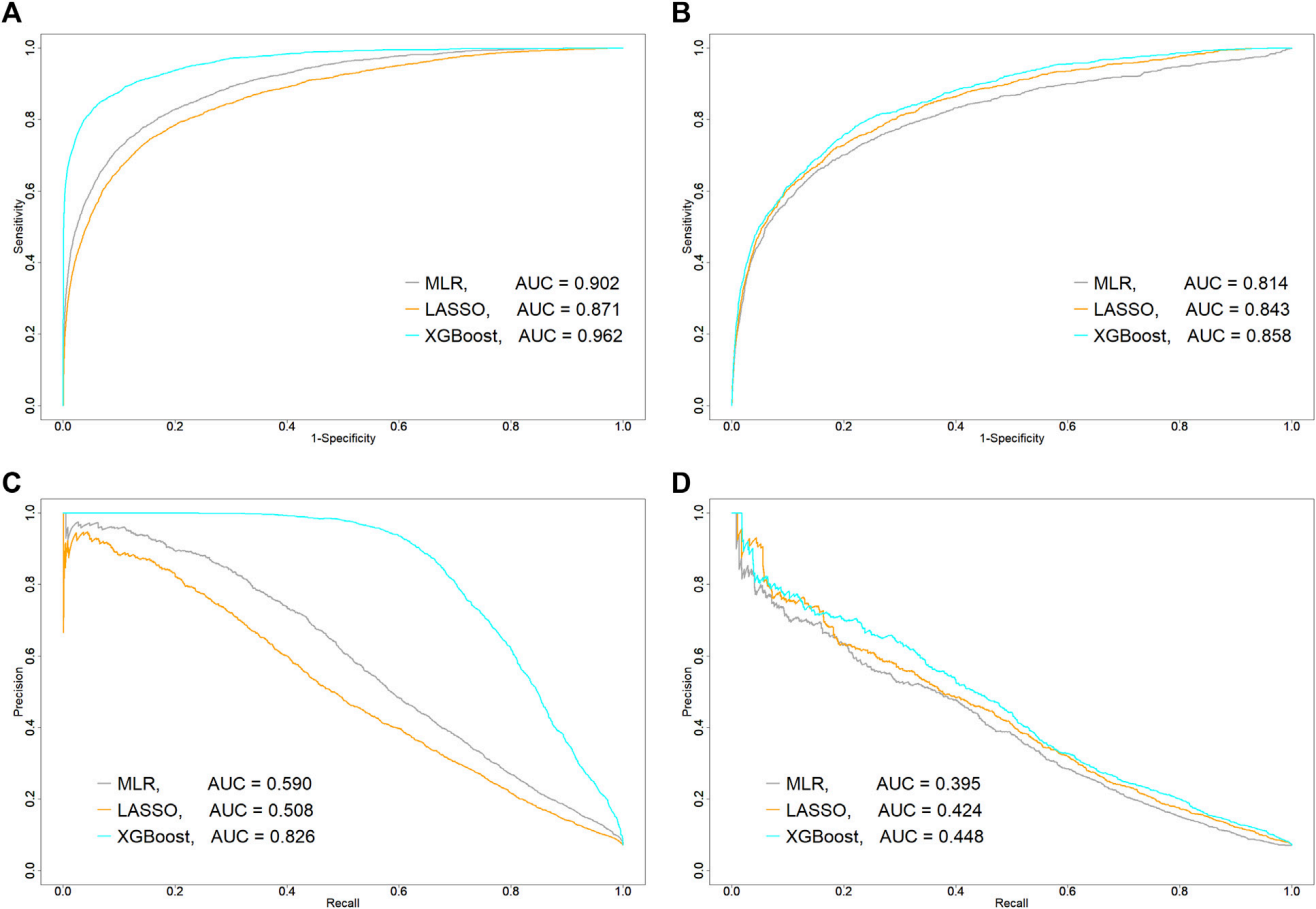
Comparison of predictive performance of each machine-learning algorithm between training and testing sets. **(A)** Area under receiver operating characteristic curve (AUROC, C statistic) in training set and **(B)** testing set. **(C)** Area under precision-recall curve (AUPR) in training set and **(D)** testing set. AUC, area under the curve; LASSO, least absolute shrinkage and selection operator; MLR, multiple logistic regression; ROC, receiver operating characteristic curve; XGBoost, extreme gradient boosting.

Confusion matrices for three machine-learning models were shown in Table 2. Among the patients (*N* = 17,524) in the testing set, 1,236 patients who experienced abnormal elevation of serum ALT (“Actual: Cases” in Table 2). The XGBoost model had the highest number of these patients and the 966 patients were correctly classified into the case group. The XGBoost model (C statistic, 0.962; 95%CI, 0.958–0.965) had the highest AUROC in the training set (Figure 1A), followed by the MLR (0.902; 0.896–0.908) and logistic LASSO regression models (0.871; 0.864–0.878), and AUROC was significantly different among the three algorithms (*p* < 0.001, respectively). However, in the testing set, AUROC in the logistic LASSO regression model (0.843; 0.830–0.854) exceeded that in the MLR model (0.814; 0.798–0.828) (*p* < 0.001). Regarding AUPR, the XGBoost model (AUPR, 0.826) was also the highest among the three algorithms in the training set, followed by MLR (0.590) and logistic LASSO regression (0.508). However, similar to AUROC, the logistic LASSO regression model (0.424) had higher AUPR than the MLR model (0.395) in the testing set. Other predictive performance metrics for the testing set are shown in Table 3. The XGBoost model had the best performance among the three algorithms, with sensitivity of 78.2%, PPV of 21.2%, specificity of 78.0%, NPV of 97.9%, and F1-score of 0.334. After this model, the logistic LASSO regression model had sensitivity of 74.8%, PPV of 20.7%, specificity of 78.3%, NPV of 97.6%, and F1-score of 0.324.

**TABLE 2:** Confusion matrices for three machine-learning models.

A		
	Actual: Cases	Actual: Controls
Predicted: Cases	906	3,790
Predicted: Controls	330	12,498

B		
	Actual: Cases	Actual: Controls
Predicted: Cases	924	3,537
Predicted: Controls	312	12,751

C		
	Actual: Cases	Actual: Controls
Predicted: Cases	966	3,590
Predicted: Controls	270	12,698

**(A)** Multiple logistic regression model, **(B)** logistic least absolute shrinkage and selection operator (LASSO) regression model, and **(C)** extreme gradient boosting (XGBoost) model. FN, false negative; FP, false positive; TN, true negative; TP, true positive.

**TABLE 3:** Prediction performance of each machine learning algorithm.

	Machine-learning models		
Evaluation metrics	MLR	Logistic LASSO regression	XGBoost
Sensitivity (recall), %	73.3	74.8	78.2
PPV (precision), %	19.3	20.7	21.2
Specificity, %	76.7	78.3	78.0
NPV, %	97.4	97.6	97.9
F1-score	0.305	0.324	0.334
AUPR	0.395	0.424	0.448
AUROC [95%CI]	0.814 [0.798–0.828]	0.843 [0.830–0.854]	0.858 [0.846–0.868]

AUPR, area under the precision-recall curve; AUROC, area under the receiver operating characteristic curve; CI, confidence interval; LASSO, least absolute shrinkage and selection operator; MLR, multiple logistic regression; NPV, negative predictive value; PPV, positive predictive value; XGBoost, extreme gradient boosting.

The magnitude of regression coefficients in each linear algorithm is shown in Supplementary Table S6. The estimated coefficients in the MLR model were very large compared with those in the logistic LASSO regression model. In addition, the MLR model showed poorly-calibrated prediction because the calibration curve was far from the diagonal plot, when compared to the other algorithms (Supplementary Figure S4). Hence, the MLR model over-fitted the training set due to the large regression coefficients, poorly-calibrated prediction, and lowest evaluation metrics for the testing set.

### Detection of Synergistic Interaction on Risk of Abnormal Elevation of ALT in Logistic LASSO Regression

**FIGURE 2:**
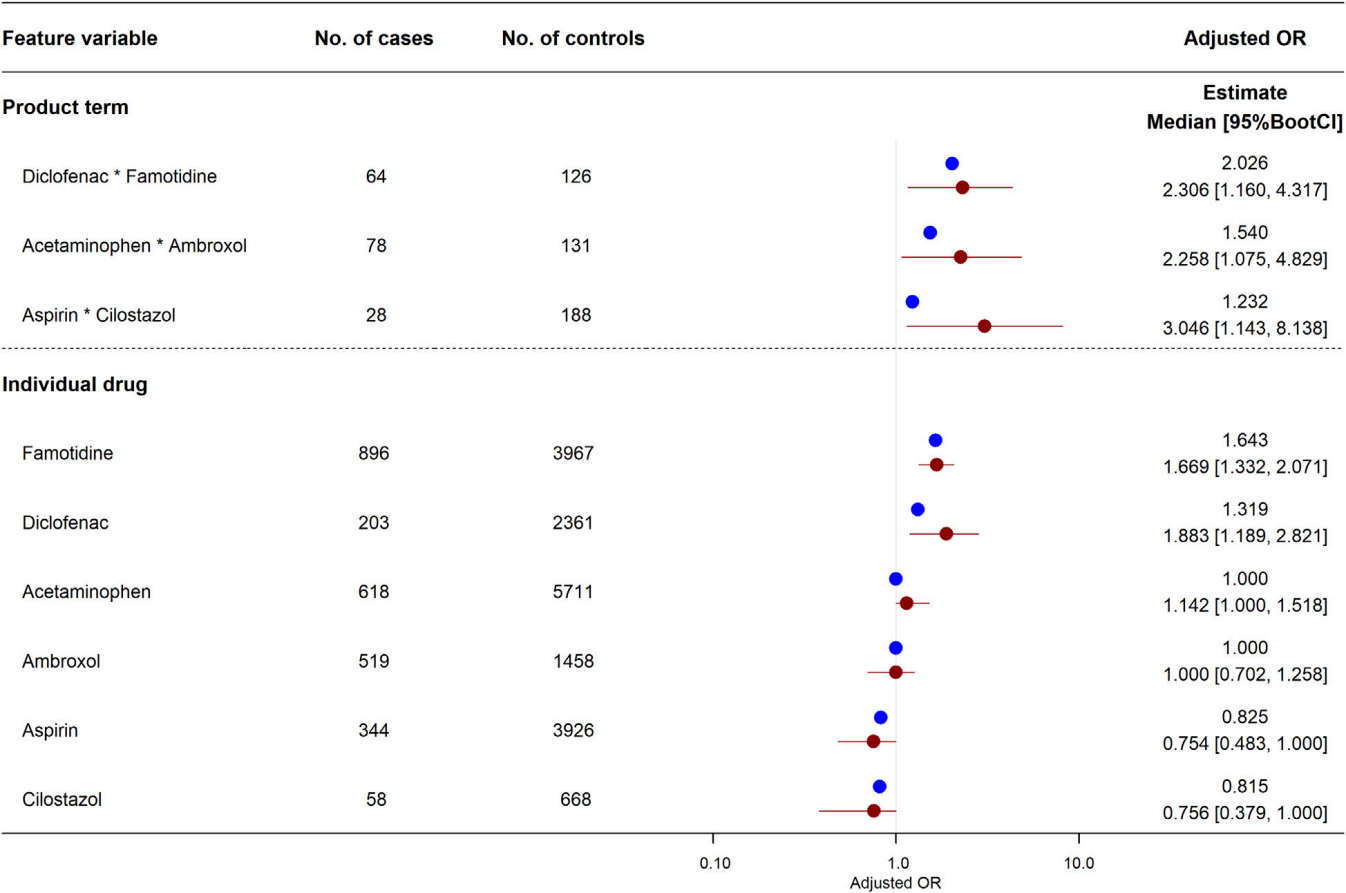
Effects of individual drugs and their product terms on risk of abnormal elevation of ALT in logistic least absolute shrinkage and selection operator (LASSO) regression model. Three product terms had a lower limit of adjusted odds ratio 95%BootCI >1. Blue and red circles represent estimated adjusted odds ratio in original training set and median of adjusted odds ratio in 2,000 bootstrap replicates, respectively. Red horizon indicates adjusted odds ratio 95%BootCI. 95%BootCI, 95% bootstrap percentile confidence interval; OR, odds ratio.

Among the 1,050 combinations, product terms in three combinations of six drugs were significantly associated with increased risk of abnormal elevation of ALT in the logistic LASSO regression equation: diclofenac*famotidine (aOR, 2.026; 95%BootCI, 1.160–4.317), acetaminophen*ambroxol (1.540; 1.075–4.829), and aspirin*cilostazol (1.232; 1.143–8.138). The associations of these three product terms and six individual drugs with abnormal elevation of ALT are shown in Figure 2. For the combination of diclofenac and famotidine, a significantly increased risk of abnormal elevation of ALT was observed for each of diclofenac (aOR, 1.319; 95%BootCI, 1.189–2.821) and famotidine (1.643; 1.332–2.071) as well as the product term. On the other hand, for the remaining two combinations, there was no significant association between the risk of abnormal elevation of ALT and each of the four individual drugs: acetaminophen (aOR, 1.000; 95%BootCI, 1.000–1.518), ambroxol (1.000; 0.702–1.258), aspirin (0.825; 0.483–1.000), and cilostazol (0.815; 0.379–1.000).

**FIGURE 3:**
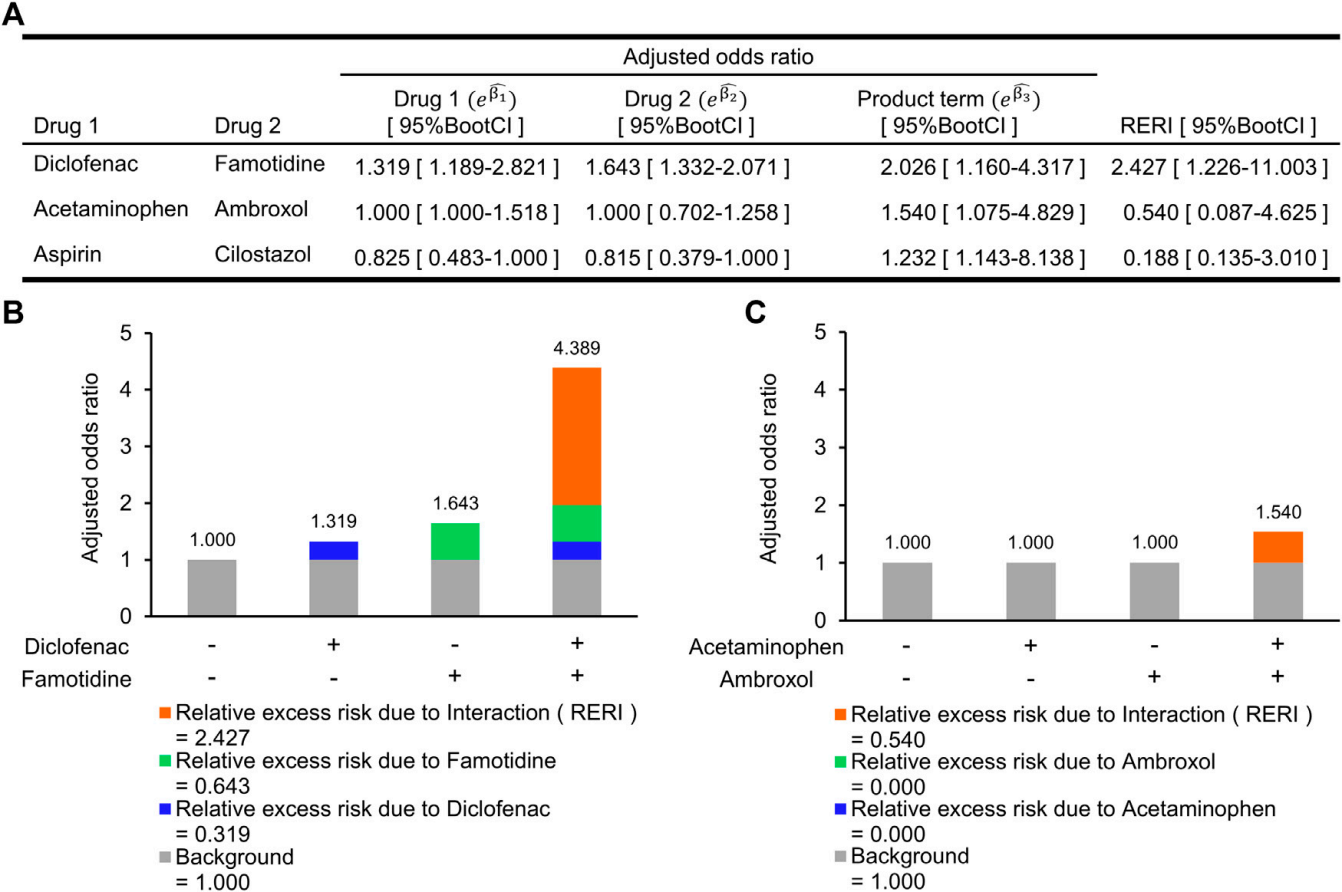
Synergistic interactions on an additive scale between two drugs on increased risk of abnormal elevation of alanine aminotransferase. **(A)** Adjusted odds ratio (aOR) for individual drugs and their interaction term, and a synergistic interaction measure. **(B)** Relative excess risk due to a combination of diclofenac and famotidine. Gray bar indicates background (i.e., non-use of diclofenac and famotidine). Blue and light green bars indicate relative excess risk due to diclofenac ( $e^{\hat{\beta_{1}}}-1$ ) and famotidine ( $e^{\hat{\beta_{2}}}-1$ ), respectively. Orange bar indicates relative excess risk due to interaction. **(C)** Relative excess risk due to a combination of acetaminophen and ambroxol. The aOR for the combination of aspirin and cilostazol ( $e^{\hat{\beta_{1}}+\hat{\beta_{2}}+\hat{\beta_{3}}}$ =0.828) was similar to the individual effects of aspirin (aOR, 0.825) and cilostazol (aOR, 0.815). 95%BootCI indicates 95% bootstrap percentile confidence interval.

The combined effect between these two drugs on the risk of abnormal elevation of ALT is shown in Figure 3. The synergistic interaction on an additive scale between diclofenac and famotidine was the greatest among the three combinations and was statistically significant (RERI, 2.427; 95%BootCI, 1.226–11.003). RERI on an additive scale of 2.427 means that the combined effect of diclofenac and famotidine is 2.427 more than the sum of the individual effects (Figure 3B). Although there was no association of the individual effect of acetaminophen and ambroxol with the risk of abnormal elevation of ALT, the synergistic interaction was statistically significant (RERI, 0.540; 95%BootCI, 0.087–4.625). The synergistic interaction between aspirin and cilostazol was also significant (RERI, 0.188; 95%BootCI, 0.135–3.010). However, aOR for abnormal elevation of ALT in each of aspirin and cilostazol tended to be lower than 1.000, and aOR when these two drugs were concomitantly used ( $e^{\hat{\beta_{1}}+\hat{\beta_{2}}+\hat{\beta_{3}}}$ =0.828) was similar to the individual effect of these drugs such as $e^{\hat{\beta_{1}}}$ and $e^{\hat{\beta_{2}}}$ (Figure 3A). Therefore, the synergistic interaction between the two drugs did not affect the increased risk of abnormal elevation of ALT. RERI and the corresponding 95%BootCI in all 1,050 product terms is shown in Supplementary Table S7.

### Feature Importance for Prediction in Gradient Boosted Decision Tree Algorithm

**FIGURE 4:**
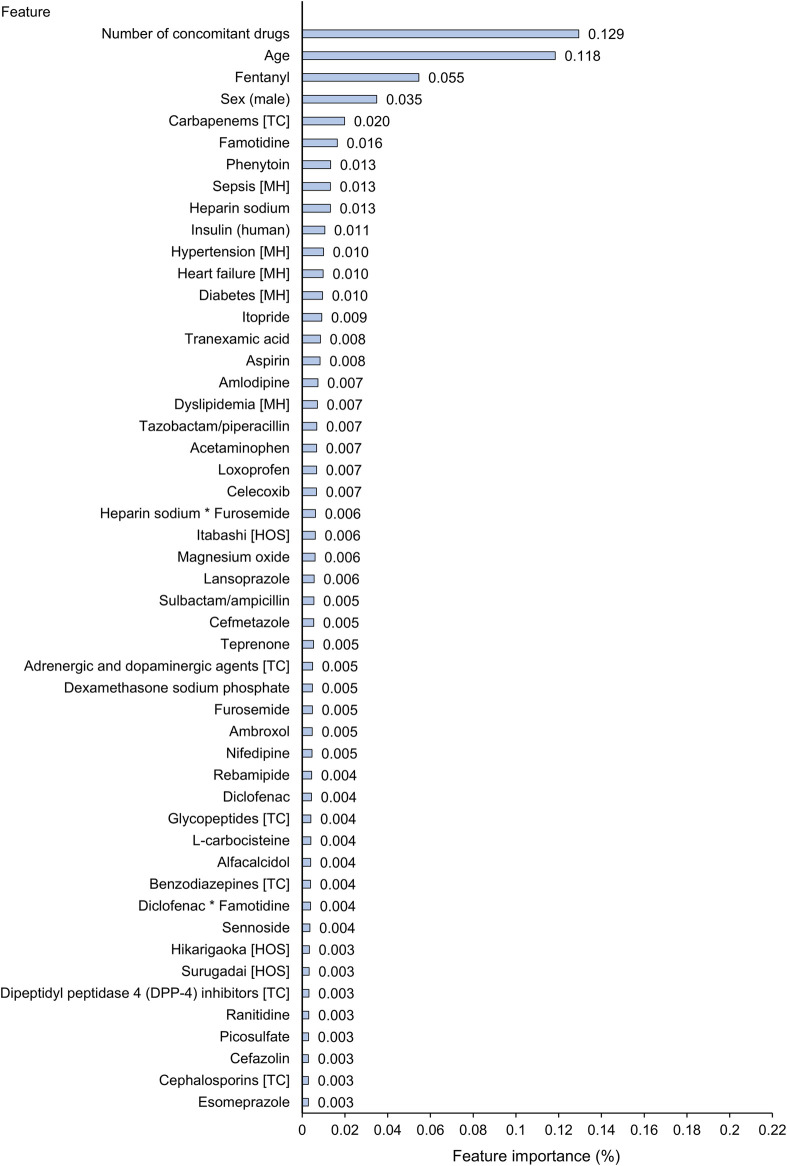
Top 50 important features for prediction of abnormal elevation of alanine aminotransferase in extreme gradient boosting (XGBoost) tree model. The first column includes the top 50 important features of all the features that were actually used in the XGBoost tree model. Feature importance (Gain) score indicates how important each feature was in the construction of boosted decision trees within the XGBoost model. A higher value of this metric when compared to another feature suggests that it is relatively more important for construction of a predictive model. The sum of all importance scores included in a trained XGBoost tree model is 1. The symbol * indicates a product term of two drugs. HOS, hospital; MH, medical history; TC, therapeutic class.

Among all the 1,375 features, the top 50 important features in the trained XGBoost model are shown in Figure 4. The number of concomitant drugs, age, use of fentanyl, sex (male), and carbapenems as a therapeutic class were detected as the most important predictors, with importance scores of 0.129, 0.118, 0.055, 0.035, and 0.020. In the combination of diclofenac and famotidine, the importance scores for the individual effects and their product term were all included in the top 50 features: diclofenac, 0.004; famotidine, 0.016; the product term, 0.004. Meanwhile the product terms of the other two combinations were not included in the top 50 important predictors.

## Discussion

In the present study, machine-learning models were developed to detect the combined effect of two drugs on the risk of abnormal elevation of serum ALT using electronic medical records. While the MLR model had high C statistics (>0.90) in the training data, this model showed all the lowest evaluation metrics in the testing data. The logistic LASSO regression and XGBoost models showed good prediction performance for the true positive patients among the positive predictions in the testing data.

In the logistic LASSO regression model, three combinations had significant synergistic interactions on the increased risk of abnormal elevation of ALT. Especially, the combined effect of diclofenac and famotidine was most strongly associated with the risk. In the XGBoost model, although individual effects and the product term of these drugs were relatively important predictors compared to the other drugs and product terms, these effects were not very significant. According to the Drug Induced Liver Injury Rank (DILIrank) dataset published by the U.S. Food and Drug Administration, diclofenac and famotidine are regarded as DILI concern drugs, and diclofenac is particularly included in the verified Most-DILI-Concern drug category. Drugs in this category have high DILI risk and have been verified as causal drugs for DILI using a standardized clinical causality assessment system such as the Roussel Uclaf Causality Assessment Method (Chen et al., 2016; U.S. Food and Drug Administration, 2020). In Japan, diclofenac is contraindicated in patients with severe hepatic impairment, and was significantly associated with increased risk of abnormal elevation of ALT in the present study. According to the National Database of Health Insurance Claims and Specific Health Checkups of Japan between April 2018 and March 2020, among non-steroidal anti-inflammatory drugs, diclofenac is commonly prescribed to outpatients, following loxoprofen, celecoxib, and acetaminophen (Ministry of Health, Labour and Welfare in Japan, 2021). Famotidine is classified in the verified Less-DILI-Concern drug category. Drugs in this category have been verified as causal drugs for DILI, and DILI is described in warnings and precautions on the package insert. In fact, famotidine was significantly associated with the risk of abnormal elevation of ALT in this study. As mentioned above, the association of diclofenac and famotidine with the risk of abnormal elevation of ALT supports the information from regulatory agencies in the U.S. and Japan, and a synergistic interaction between these drugs was detected in this study. Simultaneous administration of two drugs that are associated with an increased risk of an adverse event further increases the risk of that adverse event (i.e., synergistic interaction) (Cascorbi, 2012). In the present study, diclofenac and famotidine individually increased the risk of abnormal elevation of ALT, and a synergistic interaction between these drugs was detected. Thus, although further study is needed, it may be necessary to pay attention to liver function when diclofenac and famotidine are used together.

Acetaminophen is one of the verified Most-DILI-Concern drugs in the DILIrank, and acetaminophen-induced liver injury is described in the warning section on the package insert in Japan; that is, acetaminophen is a causal drug for DILI. However, there was no association between the individual effect of this drug and the risk of abnormal elevation of ALT in the logistic LASSO regression model. Acetaminophen is a known hepatotoxin causing intrinsic DILI, which is related to increasing dose (Ezhilarasan and Raghunandhakumar, 2021). It is proposed that the safe dose of acetaminophen to avoid liver injury is ≤ 4 g/day for an adult (Jaeschke, 2015), and the maximum approved dose of acetaminophen is 4 g/day in Japan. In addition, it is suggested that the dose of acetaminophen is not a risk factor for acetaminophen-induced liver injury in Japanese patients (Kumagai et al., 2016; Hidaka et al., 2020). For these reasons, administration of acetaminophen within the approved dose range is unlikely to affect the risk of intrinsic DILI in the real world. Ambroxol, an expectorant drug, was not associated with the risk of abnormal elevation of ALT in this study. Many *in vivo* studies have shown that ambroxol decreases serum inflammatory cytokines and liver transaminases in rodent models of liver injury due to its antioxidative and anti-inflammatory properties (Piotrowski et al., 1996; Jiang et al., 2013; Khoury et al., 2020). Meanwhile, few clinical studies have investigated the effect of ambroxol on the risk of DILI. According to the results of clinical trials and postmarketing surveillance described in the package insert, none of the patients experienced abnormal elevation of liver enzymes due to ambroxol. In the present study, although these two drugs did not individually affect the risk of abnormal elevation of ALT (aOR = 1.000, respectively), the combined effect increased the risk due to the existence of a synergistic interaction (aOR = 1.540). Thus, it is probably necessary to monitor patients who start taking acetaminophen and ambroxol, because aOR for the combined use of these drugs was larger than that for the individual drugs.

Although aspirin is one of the verified Less-DILI-Concern drugs, aspirin-induced liver injury in adults is not as well documented as Reye’s syndrome in children. Administration of aspirin to infants with viral infections such as influenza, cold, or chicken pox rarely causes Reye’s syndrome, which is defined as acute noninflammatory encephalopathy with fatty liver failure. Aspirin is an intrinsic hepatotoxin and this hepatotoxicity is supported by pre-clinical studies (Tolman, 1998). However, few clinical studies have evaluated the risk of liver injury due to aspirin in the adult population. Furthermore, in a case report, it was reported that aspirin rechallenge in an adult patient who had experienced Reye’s syndrome during his childhood did not change liver function test values (Magrum and Pickworth, 2020). For these reasons, aspirin is unlikely to increase the risk of DILI in the adult population. Cilostazol is classified into the verified Ambiguous-DILI-Concern drug category. Drugs in this category have a description of DILI in a boxed warning or warnings and precautions on the package insert, but have not been verified as the causal drug for DILI (Chen et al., 2016). Additionally, cilostazol has a protective effect on liver injury induced by ischemia-reperfusion or tioacetamide, which is a hepatotoxin (Joe et al., 2015; Fujii et al., 2017). Therefore, these drugs do not individually affect the risk of abnormal elevation of ALT. A synergistic interaction between these drugs on the risk of abnormal elevation of ALT was detected. However, as mentioned in the Results section, the risk of abnormal elevation of ALT due to concomitant use of these drugs was similar to the individual effect. Thus, concomitant use of aspirin and cilostazol is unlikely to increase the risk of abnormal elevation of ALT.

In conclusion, we developed machine-learning models that predicted an abnormal increase of serum ALT more accurately than did traditional statistical models such as MLR. Furthermore, not only do diclofenac and famotidine individually affect the risk of abnormal elevation of serum ALT, but the combination of these drugs can further increase that risk.

## Data Availability

The raw data supporting the conclusions of this article will be made available by the authors, without undue reservation.

## References

[B1] Akimoto H. Nagashima T. Minagawa K. Hayakawa T. Takahashi Y. Asai S. (2021). Signal Detection of Potential Hepatotoxic Drugs: Case-Control Study Using Both a Spontaneous Reporting System and Electronic Medical Records. Biol. Pharm. Bull. 44, 1514–1523. 10.1248/bpb.b21-00407 34602560

[B2] Björnsson E. Olsson R. (2005). Outcome and Prognostic Markers in Severe Drug-Induced Liver Disease. Hepatology 42, 481–489. 10.1002/hep.20800 16025496

[B3] Cascorbi I. (2012). Drug Interactions-Pprinciples, Examples and Clinical Consequences. Dtsch. Arztebl. Int. 109, 546–556. 10.3238/arztebl.2012.0546 23152742 PMC3444856

[B4] Chalasani N. Bonkovsky H. L. Fontana R. Lee W. Stolz A. Talwalkar J. (2015). Features and Outcomes of 899 Patients with Drug-Induced Liver Injury: The DILIN Prospective Study. Gastroenterology 148, 1340–e7. 10.1053/j.gastro.2015.03.006 25754159 PMC4446235

[B5] Chen M. Suzuki A. Thakkar S. Yu K. Hu C. Tong W. (2016). DILIrank: the Largest Reference Drug List Ranked by the Risk for Developing Drug-Induced Liver Injury in Humans. Drug Discov. Today 21, 648–653. 10.1016/j.drudis.2016.02.015 26948801

[B6] Church R. J. Watkins P. B. (2018). In Silico modeling to Optimize Interpretation of Liver Safety Biomarkers in Clinical Trials. Exp. Biol. Med. (Maywood) 243, 300–307. 10.1177/1535370217740853 29096561 PMC5813867

[B7] Clark J. M. Brancati F. L. Diehl A. M. (2003). The Prevalence and Etiology of Elevated Aminotransferase Levels in the United States. Am. J. Gastroenterol. 98, 960–967. 10.1111/j.1572-0241.2003.07486.x 12809815

[B8] Cruz-Monteagudo M. Cordeiro M. N. Borges F. (2008). Computational Chemistry Approach for the Early Detection of Drug-Induced Idiosyncratic Liver Toxicity. J. Comput. Chem. 29, 533–549. 10.1002/jcc.20812 17705164

[B9] de Abajo F. J. Montero D. Madurga M. García Rodríguez L. A. (2004). Acute and Clinically Relevant Drug-Induced Liver Injury: A Population Based Case-Control Study. Br. J. Clin. Pharmacol. 58, 71–80. 10.1111/j.1365-2125.2004.02133.x 15206996 PMC1884531

[B10] Ekins S. Williams A. J. Xu J. J. (2010). A Predictive Ligand-Based Bayesian Model for Human Drug-Induced Liver Injury. Drug Metab. Dispos. 38, 2302–2308. 10.1124/dmd.110.035113 20843939

[B11] Ezhilarasan D. Raghunandhakumar S. (2021). Boldine Treatment Protects Acetaminophen‐induced Liver Inflammation and Acute Hepatic Necrosis in Mice. J. Biochem. Mol. Toxicol. 35, e22697. 10.1002/jbt.22697 33393705

[B12] Fujii T. Obara H. Matsubara K. Fujimura N. Yagi H. Hibi T. (2017). Oral Administration of Cilostazol Improves Survival Rate after Rat Liver Ischemia/reperfusion Injury. J. Surg. Res. 213, 207–214. 10.1016/j.jss.2017.02.020 28601316

[B13] Hidaka N. Kaji Y. Takatori S. Tanaka A. Matsuoka I. Tanaka M. (2020). Risk Factors for Acetaminophen-Induced Liver Injury: A Single-Center Study from Japan. Clin. Ther. 42, 704–710. 10.1016/j.clinthera.2020.02.003 32145905

[B14] Jaeschke H. (2015). Acetaminophen: Dose-dependent Drug Hepatotoxicity and Acute Liver Failure in Patients. Dig. Dis. 33, 464–471. 10.1159/000374090 26159260 PMC4520394

[B15] Jiang K. Wang X. Mao X. Lao H. Zhang J. Wang G. (2013). Ambroxol Alleviates Hepatic Ischemia Reperfusion Injury by Antioxidant and Antiapoptotic Pathways. Transpl. Proc. 45, 2439–2445. 10.1016/j.transproceed.2013.04.007 23953561

[B16] Joe Y. Zheng M. Kim H. J. Uddin M. J. Kim S. K. Chen Y. (2015). Cilostazol Attenuates Murine Hepatic Ischemia and Reperfusion Injury via Heme Oxygenase-dependent Activation of Mitochondrial Biogenesis. Am. J. Physiol. Gastrointest. Liver Physiol. 309, G21–G29. 10.1152/ajpgi.00307.2014 25951827

[B17] Jung K. Lee J. Gupta V. Cho G. (2019). Comparison of Bootstrap Confidence Interval Methods for GSCA Using a Monte Carlo Simulation. Front. Psychol. 10, 2215. 10.3389/fpsyg.2019.02215 31681066 PMC6797821

[B18] Khoury T. Ishay Y. Rotnemer-Golinkin D. Zolotarovya L. Arkadir D. Zimran A. (2020). A Synergistic Effect of Ambroxol and Beta-Glucosylceramide in Alleviating Immune-Mediated Hepatitis: A Novel Immunomodulatory Non-immunosuppressive Formulation for Treatment of Immune-Mediated Disorders. Biomed. Pharmacother. 132, 110890. 10.1016/j.biopha.2020.110890 33080465

[B19] Kim S. M. Kim Y. Jeong K. Jeong H. Kim J. (2018). Logistic LASSO Regression for the Diagnosis of Breast Cancer Using Clinical Demographic Data and the BI-RADS Lexicon for Ultrasonography. Ultrasonography 37, 36–42. 10.14366/usg.16045 28618771 PMC5769953

[B20] Knol M. J. van der Tweel I. Grobbee D. E. Numans M. E. Geerlings M. I. (2007). Estimating Interaction on an Additive Scale between Continuous Determinants in a Logistic Regression Model. Int. J. Epidemiol. 36, 1111–1118. 10.1093/ije/dym157 17726040

[B21] Kumagai Y. Tanaka R. Song I. Sakamoto Y. (2016). Analysis of Data from Special Drug Use Surveillance on Elevation of Liver Function Tests in Japanese Patients Administered High Dose Acetaminophen. Jpn. J. Clin. Pharmacol. Ther. 47, 31–37. 10.3999/jscpt.47.31

[B22] Lee W. M. (2013). Drug-induced Acute Liver Failure. Clin. Liver Dis. 17, 575–viii. 10.1016/j.cld.2013.07.001 24099019 PMC3838908

[B23] Liu Z. Que S. Xu J. Peng T. (2014). Alanine Aminotransferase-Old Biomarker and New Concept: a Review. Int. J. Med. Sci. 11, 925–935. 10.7150/ijms.8951 25013373 PMC4081315

[B24] Liu Z. Shi Q. Ding D. Kelly R. Fang H. Tong W. (2011). Translating Clinical Findings into Knowledge in Drug Safety Evaluation-Ddrug Induced Liver Injury Prediction System (DILIps). PLoS Comput. Biol. 7, e1002310. 10.1371/journal.pcbi.1002310 22194678 PMC3240589

[B25] Low Y. Uehara T. Minowa Y. Yamada H. Ohno Y. Urushidani T. (2011). Predicting Drug-Induced Hepatotoxicity Using QSAR and Toxicogenomics Approaches. Chem. Res. Toxicol. 24, 1251–1262. 10.1021/tx200148a 21699217 PMC4281093

[B26] Magrum B. G. Pickworth K. K. (2020). Aspirin Rechallenge in an Adult Patient Previously Diagnosed with Reye Syndrome. Am. J. Health Syst. Pharm. 77, 123–127. 10.1093/ajhp/zxz276 31788685

[B27] Meier Y. Cavallaro M. Roos M. Pauli-Magnus C. Folkers G. Meier P. J. (2005). Incidence of Drug-Induced Liver Injury in Medical Inpatients. Eur. J. Clin. Pharmacol. 61, 135–143. 10.1007/s00228-004-0888-z 15726344

[B28] Ministry of Health, Labour and Welfare in Japan (2021). The National Database of Health Insurance Claims and Specific Health Checkups of Japan . https://www.mhlw.go.jp/stf/seisakunitsuite/bunya/0000177182.html (Accessed January 14, 2022).

[B29] Piotrowski W. J. Pietras T. Kurmanowska Z. Nowak D. Marczak J. Marks-Kończalik J. (1996). Effect of Paraquat Intoxication and Ambroxol Treatment on Hydrogen Peroxide Production and Lipid Peroxidation in Selected Organs of Rat. J. Appl. Toxicol. 16, 501–507. 10.1002/(SICI)1099-1263(199611)16:6<501::AID-JAT379>3.0.CO;2-Z 8956096

[B30] Pratt D. S. Kaplan M. M. (2000). Evaluation of Abnormal Liver-Enzyme Results in Asymptomatic Patients. N. Engl. J. Med. 342, 1266–1271. 10.1056/NEJM200004273421707 10781624

[B31] Robles-Diaz M. Lucena M. I. Kaplowitz N. Stephens C. Medina-Cáliz I. González-Jimenez A. (2014). Use of Hy's Law and a New Composite Algorithm to Predict Acute Liver Failure in Patients with Drug-Induced Liver Injury. Gastroenterology 147, 109–e5. 10.1053/j.gastro.2014.03.050 24704526

[B32] Rodgers A. D. Zhu H. Fourches D. Rusyn I. Tropsha A. (2010). Modeling Liver-Related Adverse Effects of Drugs Using Knearest Neighbor Quantitative Structure-Activity Relationship Method. Chem. Res. Toxicol. 23, 724–732. 10.1021/tx900451r 20192250 PMC2965736

[B33] Saito T. Rehmsmeier M. (2015). The Precision-Recall Plot Is More Informative Than the ROC Plot when Evaluating Binary Classifiers on Imbalanced Datasets. PLoS One 10, e0118432. 10.1371/journal.pone.0118432 25738806 PMC4349800

[B34] Senior J. R. (2007). Drug Hepatotoxicity from a Regulatory Perspective. Clin. Liver Dis. 11, 507–vi. 10.1016/j.cld.2007.06.002 17723917

[B35] Sgro C. Clinard F. Ouazir K. Chanay H. Allard C. Guilleminet C. (2002). Incidence of Drug-Induced Hepatic Injuries: a French Population-Based Study. Hepatology 36, 451–455. 10.1053/jhep.2002.34857 12143055

[B36] Shen T. Liu Y. Shang J. Xie Q. Li J. Yan M. (2019). Incidence and Etiology of Drug-Induced Liver Injury in Mainland China. Gastroenterology 156, 2230–e11. 10.1053/j.gastro.2019.02.002 30742832

[B37] Suzuki A. Yuen N. Walsh J. Papay J. Hunt C. M. Diehl A. M. (2009). Co-medications that Modulate Liver Injury and Repair Influence Clinical Outcome of Acetaminophen-Associated Liver Injury. Clin. Gastroenterol. Hepatol. 7, 882–888. 10.1016/j.cgh.2009.03.034 19362607

[B38] Suzuki A. Yuen N. A. Ilic K. Miller R. T. Reese M. J. Brown H. R. (2015). Comedications Alter Drug-Induced Liver Injury Reporting Frequency: Data Mining in the WHO VigiBase^TM^ . Regul. Toxicol. Pharmacol. 72, 481–490. 10.1016/j.yrtph.2015.05.004 25988394 PMC4548888

[B39] Takikawa H. Murata Y. Horiike N. Fukui H. Onji M. (2009). Drug-induced Liver Injury in Japan: An Analysis of 1676 Cases between 1997 and 2006. Hepatol. Res. 39, 427–431. 10.1111/j.1872-034X.2008.00486.x 19207579

[B40] Teschke R. Eickhoff A. Schulze J. (2013). Drug- and Herb-Induced Liver Injury in Clinical and Translational Hepatology: Causality Assessment Methods, Quo Vadis? J. Clin. Transl. Hepatol. 1, 59–74. 10.14218/JCTH.2013.D002X 26357608 PMC4521275

[B41] Tolman K. G. (1998). Hepatotoxicity of Non-narcotic Analgesics. Am. J. Med. 105, 13S–19S. 10.1016/s0002-9343(98)00070-9 9715830

[B42] Uetake H. Sugihara K. Muro K. Sunaya T. Horiuchi-Yamamoto Y. Takikawa H. (2018). Clinical Features of Regorafenib-Induced Liver Injury in Japanese Patients from Postmarketing Experience. Clin. Colorectal Cancer 17, e49–e58. 10.1016/j.clcc.2017.09.004 29074353

[B43] U.S. Food and Drug Administration (2020). Drug Induced Liver Injury Rank (DILIrank) Dataset . https://www.fda.gov/science-research/liver-toxicity-knowledge-base-ltkb/drug-induced-liver-injury-rank-dilirank-dataset (Accessed January 11, 2022).

[B44] Yazici C. Mutlu E. Bonkovsky H. L. Russo M. W. (2015). Risk Factors for Severe or Fatal Drug-Induced Liver Injury from Amoxicillin-Clavulanic Acid. Hepatol. Res. 45, 676–682. 10.1111/hepr.12410 25163514

[B45] Zhu X. W. Sedykh A. Liu S. S. (2014). Hybrid In Silico Models for Drug-Induced Liver Injury Using Chemical Descriptors and In Vitro Cell-Imaging Information. J. Appl. Toxicol. 34, 281–288. 10.1002/jat.2879 23640866

