# Supplementary material for: Detection of Synergistic Interaction on an Additive Scale Between Two Drugs on Abnormal Elevation of Serum Alanine Aminotransferase Using Machine-Learning Algorithms
Source: Front Pharmacol. 2022 Jul 6;13:910205. doi: 10.3389/fphar.2022.910205 (PMC9298751; doi:10.3389/fphar.2022.910205)
Supplement: Supplementary file 1 [file DataSheet1.pdf]

Supplementary Figures

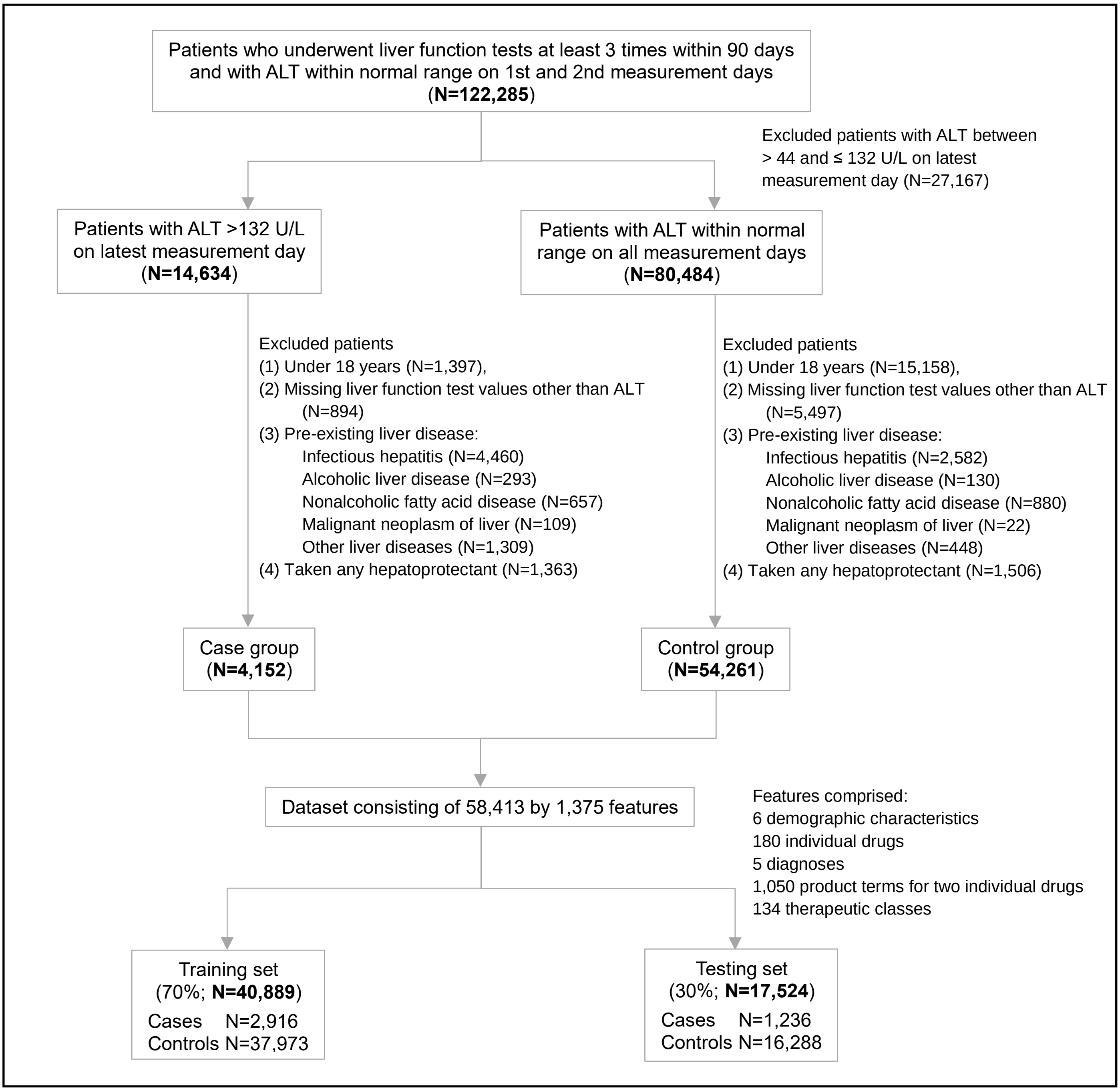

Supplementary Figure S1. Sample size flowchart.

Abbreviations: ALT, alanine aminotransferase; U/L, units per liter.

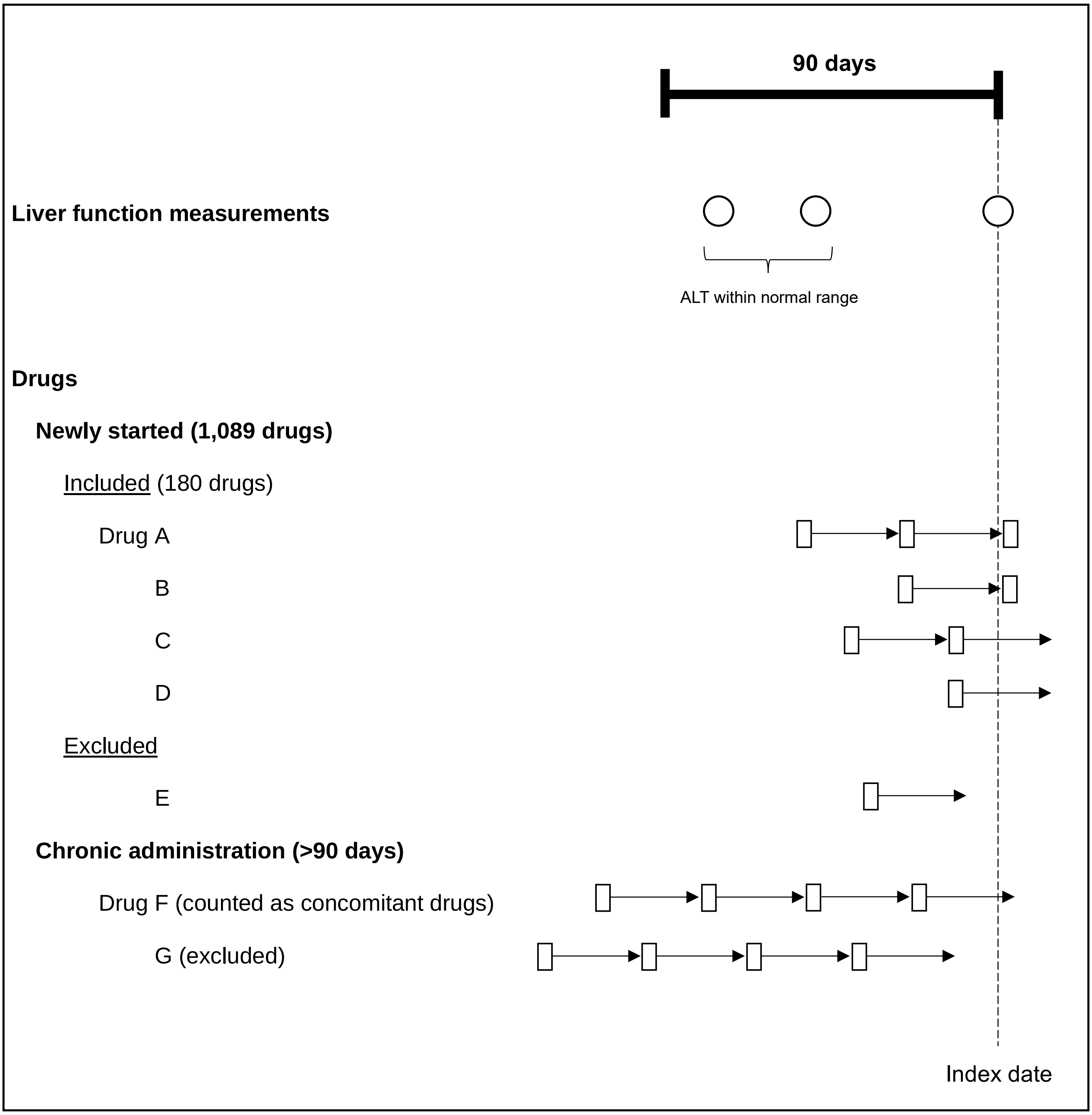

Supplementary Figure S2. Definition of drugs treated as features in this study.

Subjects who showed abnormal elevation of serum ALT on the index date were referred to as cases, and controls were subjects who did not. Opened circles represent dates of liver function measurement. Opened boxes and arrows represent prescription dates and days of supply, respectively. If the number of patients who had been using drugs A and B in combination at least until the index date in all extracted patients (N=58,413) was 100 or more, the following three variables were regarded as features (binary data): use of drug A, use of drug B, and a product term of the two drugs. The number of drugs that continued to be used at least until the index date regardless of the start date was regarded as the number of concomitant drugs (drugs A-D and F). Drugs discontinued before the index date were excluded from analysis (drugs E and G). Abbreviation: ALT, alanine aminotransferase.

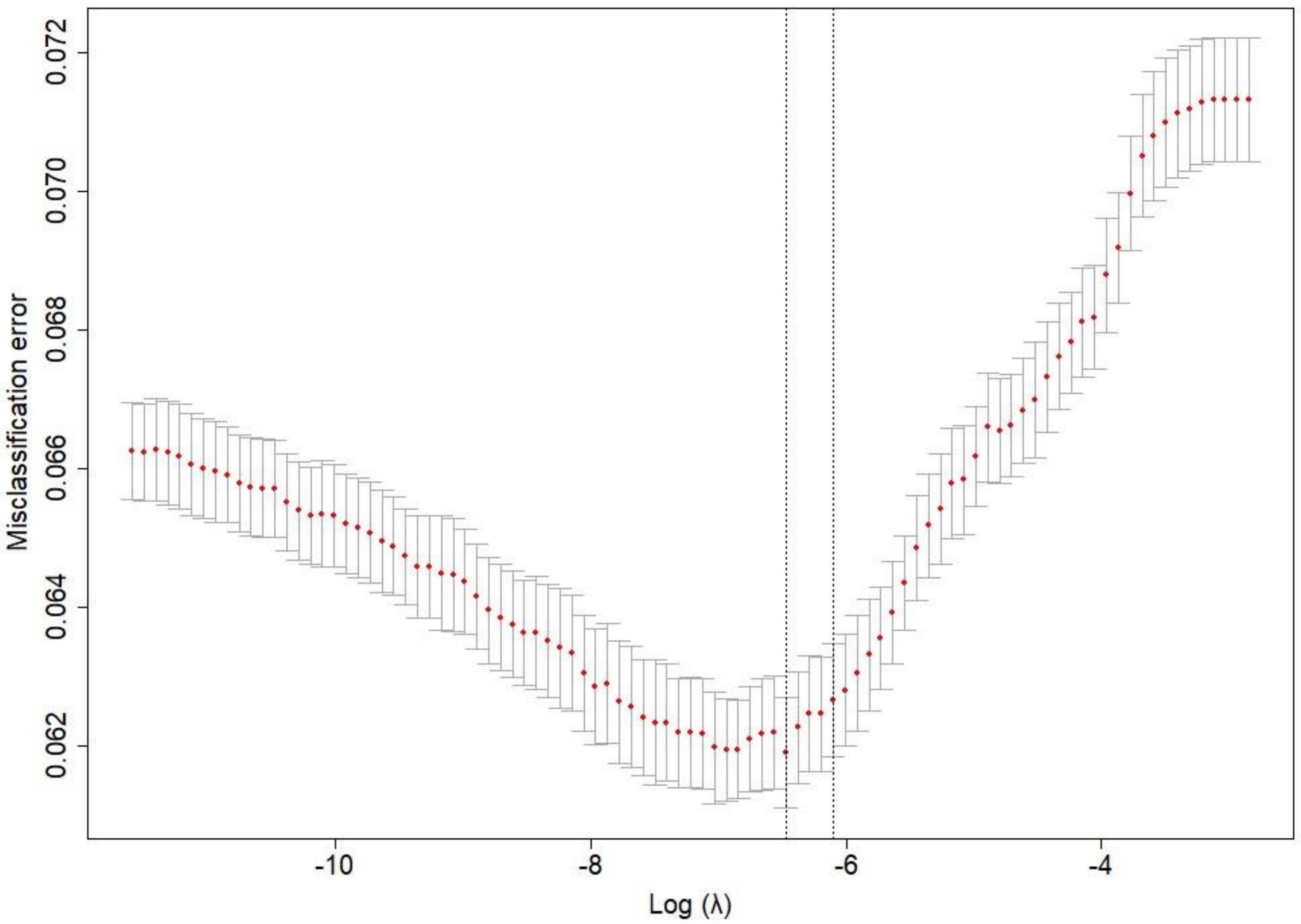

Supplementary Figure S3. Cross validation plot in logistic least absolute shrinkage and selection operator (LASSO) regression model.

Ten-fold cross validation plot for the penalty term in the loss function. Lambda (λ) is the penalty term in the loss function during training; a larger λ results in more coefficients being forced to be zero. For the present hyperparameter tuning in the logistic LASSO regression model, λ values ranged from 0.000009 to 0.058160 with the minimum misclassification error rate achieved at 0.001545 (Log (λ) = -6.473).

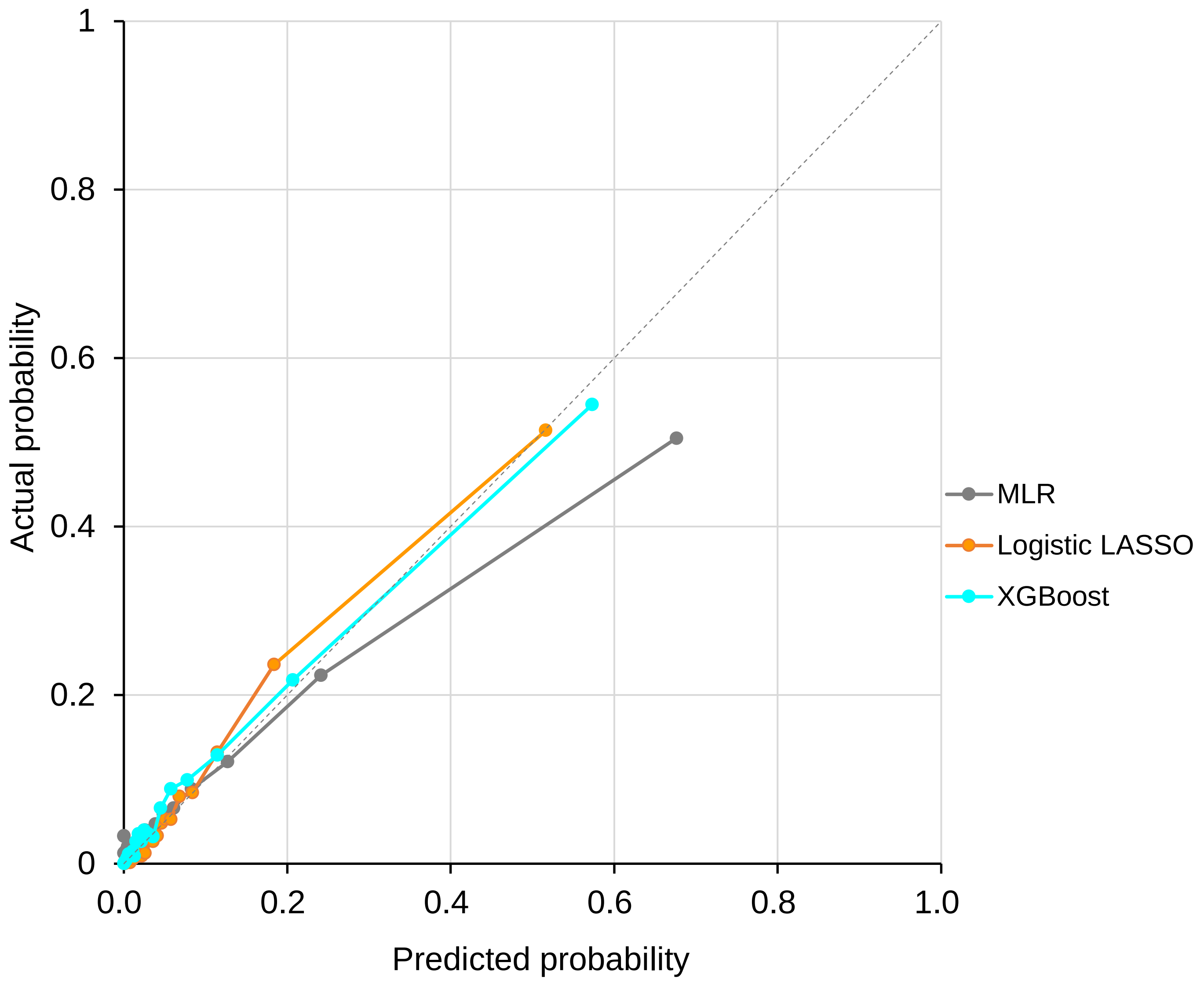

Supplementary Figure S4. Calibration plot for each machine-learning algorithm.

Graphical assessment of calibration is possible with predictions on the x-axis and the outcome on the y-axis. Perfect predictions should be on the 45° line. Patients were divided into 20 groups according to their predicted probability in a machine-learning algorithm, and then the predicted probability was plotted against the actual probability for each group of patients. Abbreviations: LASSO, least absolute shrinkage and selection operator; MLR, multiple logistic regression; XGBoost, extreme gradient boosting.
