# Supplementary material for: Detection of Synergistic Interaction on an Additive Scale Between Two Drugs on Abnormal Elevation of Serum Alanine Aminotransferase Using Machine-Learning Algorithms
Source: Front Pharmacol. 2022 Jul 6;13:910205. doi: 10.3389/fphar.2022.910205 (PMC9298751; doi:10.3389/fphar.2022.910205)
Supplement: Supplementary file 2 [file DataSheet2.docx]

**Supplementary Tables**

**Supplementary Table S1. International Classification of Disease version 10 (ICD-10) codes of excluded pre-existing liver disease.**

| **Category** | **Disease** | **ICD-10 code** |
| --- | --- | --- |
| Infectious hepatitis | Herpes viral hepatitis | B00.8+K77.0 |
|  | Hepatitis A | B15 |
|  | Hepatitis B | B16, B17.0, B18.0, B18.1 |
|  | Hepatitis C | B17.1, B18.2 |
|  | Hepatitis E | B17.2 |
|  | Cytomegalovirus hepatitis | B25.1 |
|  | Amebic liver abscess | A06.4 |
|  | Liver tuberculosis | A18.8+K77.0 |
|  | Syphilis of liver | A52.7+K77.0 |
| Alcoholic liver disease | Alcoholic fatty liver | K70.0 |
|  | Alcoholic hepatitis | K70.1 |
|  | Alcoholic fibrosis and sclerosis of liver | K70.2 |
|  | Alcoholic cirrhosis of liver | K70.3 |
|  | Alcoholic hepatic failure | K70.4 |
|  | Alcoholic liver disease, unspecified | K70.9 |
| Nonalcoholic fatty liver disease | Nonalcoholic steatohepatitis (NASH) | K75.8 |
|  | Nonalcoholic fatty liver disease (NAFLD) | K76.0 |
| Malignant neoplasm of liver and intrahepatic bile ducts | Liver cell carcinoma | C22.0 |
|  | Intrahepatic bile duct carcinoma | C22.1 |
|  | Angiosarcoma of liver | C22.3 |
| Other causes | Primary biliary cirrhosis, Primary biliary cholangitis | K74.3 |
|  | Autoimmune hepatitis | K75.4 |

**Supplementary Table S2. Classification of less frequently used drugs into 134 therapeutic classes.**

| **No.** | **Therapeutic class (Feature)** | **ATC 4^th^ levels** | **Note** |
| --- | --- | --- | --- |
| 1 | Aminosalicylic acids | A07EC | Agents for autoimmune diseases |
| 2 | Antirheumatic drugs | M01CB, M01CC |  |
| 3 | Immunosuppressive drugs | L04AA, L04AD, L04AX |  |
| 4 | Interferons | L03AB |  |
| 5 | Fibrates | C10AB | Agents for dyslipidemia |
| 6 | Polyunsaturated fatty acids | C10AX |  |
| 7 | Statins | C10AA |  |
| 8 | Other antidyslipidemic drugs | C10AC, C10AX, V03AE |  |
| 9 | Active vitamine D3 preparations | A11CC | Agents for osteoporosis |
| 10 | Bisphosphonates | M05BA |  |
| 11 | Other drugs for treatment of osteoporosis | H05AA, H05BA, M05BX |  |
| 12 | Drugs for treatment of neuropathic pain | N02BG, N03AX | Analgesics |
| 13 | Non-steroidal anti-inflammatory drugs (NSAIDs) | M01AB, M01AC M01AE, M01AG, M01AH, N02BA, N02BE |  |
| 14 | Opioids | N01AH, N02A-E, N02AX |  |
| 15 | Selective serotonin 5-HT_1_ receptor agonists | N02CC |  |
| 16 | Amide local anesthetics | N01BB | Anesthetics |
| 17 | General anesthetics | N01AX, N05CM |  |
| 18 | Histamine (H_1_) receptor antagonists | R01AC, R06AA, R06AB, R06AD, R06AE, R06AX | Antiallergic agents |
| 19 | Leukotriene receptor antagonists | R03DC |  |
| 20 | Other anti-allergy drugs | R03BC, R03DX |  |
| 21 | Aminoglycosides | J01GB | Antibiotics |
| 22 | Anti-herpes drugs | J05AB |  |
| 23 | Antiparasitic drugs | P01BA, P01BC, P02BA, P02CC, P02CF |  |
| 24 | Antiretroviral drugs | J05AE, J05AF, J05AJ |  |
| 25 | Antiviral drugs for cytomegalovirus disease | J05AB |  |
| 26 | Azole antifungals | J02AB, J02AC |  |
| 27 | Carbapenems | J01DH |  |
| 28 | Cephalosporins | J01DB-E |  |
| 29 | Fluoroquinolones | J01MA |  |
| 30 | Glucocorticoids | H02AB |  |
| 31 | Glycopeptides | J01XA |  |
| 32 | Influenza antiviral drugs | J05AH |  |
| 33 | Macrolides | J01FA |  |
| 34 | Penicillins | J01CA, J01CB |  |
| 35 | Polyene macrolide antibiotics | G01AA |  |
| 36 | Tetracyclines | J01AA |  |
| 37 | Other antibiotic drugs | J01BA, J01DF, J01DI, J01FA, J01FF, J01M, J01XB, J01XD, J01XX |  |
| 38 | Other antimycotic drugs | D01BA, J02AX, P01CX |  |
| 39 | Dipeptidyl peptidase 4 (DPP-4) inhibitors | A10BH | Antidiabetics |
| 40 | Glinides | A10BX |  |
| 41 | Glucagon-like peptide-1 (GLP-1) analogues | A10BJ |  |
| 42 | Other insulins | A10AB-E |  |
| 43 | Sodium-glucose co-transporter 2 (SGLT2) inhibitors | A10BK |  |
| 44 | Sulfonylureas | A10BB |  |
| 45 | α-glucosidase inhibitors | A10BF |  |
| 46 | Angiotensin receptor blockers (ARBs) | C09CA | Antihypertensive agents |
| 47 | Angiotensin-converting enzyme (ACE) inhibitors | C09AA |  |
| 48 | Antihypertensives for pulmonary arterial hypertension | C02KX |  |
| 49 | Calcium channel blockers | C08CA |  |
| 50 | Diuretics | C03AA, C03BA, C03CA, C03XA |  |
| 51 | Potassium-sparing diuretics | C03DA, C03DB |  |
| 52 | Other vasodilators | C01EB, C02AA-C, C02DB, C04AA, C04AF, C09XA |  |
| 53 | Antiplatelet drugs | B01AC, C01DX | Antithrombotic agents |
| 54 | Heparins | B01AB |  |
| 55 | Novel oral anticoagulants (NOACs) | B01AE, B01AF |  |
| 56 | Prostaglandins | B01AC |  |
| 57 | Other anticoagulant drugs | B01AB, B01AD, B01AX |  |
| 58 | Barbiturates | N03AA, N05CA | Anxiolytic agents |
| 59 | Benzodiazepines | N05BA, N05CD |  |
| 60 | Other anxiolytic drugs | N05CC, N05CH, N05CM |  |
| 61 | Antiarrhythmic drugs | C01BA-D, C01BG, C07AA | Cardiovascular agents |
| 62 | Digitalis glycosides | C01AA |  |
| 63 | Organic nitrates | C01DA |  |
| 64 | Other drugs for treatment of heart failure | C01CE |  |
| 65 | Antiepileptic drugs | N03AB-D, N03AF, N03AG, N03AX | Central nervous system agents |
| 66 | Butyrophenone derivatives | N05AD |  |
| 67 | Centrally acting anticholinergic drugs | N04AA |  |
| 68 | Dopamine receptor agonists | N04BC |  |
| 69 | Drugs for treatment of Alzheimer's disease | N06DA, N06DX |  |
| 70 | Drugs for treatment of dizziness/vertigo | C04AX, N07CA |  |
| 71 | Drugs for treatment of ischemic stroke | N06BX, N07XX |  |

**Supplementary Table S2. Continued.**

| **No.** | **Therapeutic class (Feature)** | **ATC 4^th^ levels** | **Note** |
| --- | --- | --- | --- |
| 72 | Drugs used for attention-deficit/hyperactivity disorder | C02AC, N06BA | Central nervous system agents |
| 73 | Ergot alkaloids | C04AE, G02AB, G02CB |  |
| 74 | Multi-acting receptor-targeted antipsychotics (MARTAs) | N05AH |  |
| 75 | Phenothiazine derivatives | N05AA-C |  |
| 76 | Selective serotonin reuptake inhibitors (SSRIs) | N06AB |  |
| 77 | Serotonin dopamine antagonists (SDAs) | N05AX |  |
| 78 | Serotonin noradrenaline reuptake inhibitors (SNRIs) | N06AX |  |
| 79 | Tetracyclic antidepressants | N06AA, N06AX |  |
| 80 | Tricyclic antidepressants | N06AA |  |
| 81 | Other antipsychotic drugs | N05AE, N05AG, N05AL, N05AN, N05AX |  |
| 82 | Other drugs for treatment of Parkinson's disease | N04BB, N04BD, N04BX |  |
| 83 | Adrenergic and dopaminergic agents | C01CA | Drugs affecting the autonomic nervous system |
| 84 | Beta blocking agents | C07AA, C07AB |  |
| 85 | α/β-Adrenergic receptor blockers | C07AG |  |
| 86 | α-Adrenergic receptor blockers | C02CA |  |
| 87 | α-Adrenergic receptor blockers for treatment of benign prostatic hyperplasia | G04CA |  |
| 88 | Antacids | A02AA-C | Drugs for alimentary tract and metabolic diseases |
| 89 | Cholekinetics | A03AA, A03AB |  |
| 90 | Drugs for constipation | A06AB, A06AD, A06AG, A06AX |  |
| 91 | Enzyme preparations for digestion disorders | A09AA |  |
| 92 | Histamine (H2) receptor antagonists | A02BA |  |
| 93 | Other anti-ulcer drugs | A16AA, A02BB, A02BX |  |
| 94 | Antihyperuricemic drugs | M04AA, M04AB, V03AF | Drugs for urinary tract diseases |
| 95 | Drugs for treatment of chronic kidney disease | V03AE |  |
| 96 | Drugs for urinary frequency and incontinence | G04BD |  |
| 97 | Phosphodiesterase-5 (PDE-5) inhibitors | G04BE |  |
| 98 | 5α-reductase inhibitors | G04CB | Holmone-related drugs |
| 99 | Anterior pituitary lobe hormones and analogues | H01AA, H01AC |  |
| 100 | Anti-androgens | G03DC, L01XX, L02BB, L02BX |  |
| 101 | Drugs used for Cushing's syndrome | H02CA, L01XX, V04CD |  |
| 102 | Estrogens | G03CA |  |
| 103 | Gonadotropin releasing hormone analogues | L02AE |  |
| 104 | Gonadotropins | G03GA |  |
| 105 | Posterior pituitary lobe hormones | H01BA, H01BB |  |
| 106 | Progestogens | G03AC, G03DA, G03DB |  |
| 107 | Selective estrogen receptor modulators (SERMs) | L02BA, G03GB, G03XC |  |
| 108 | Other hormone preparations | H04AA, V04CJ |  |
| 109 | Other sexhormones | G03XA |  |
| 110 | Molecular targeted drugs (Monoclonal antibodies) | C10AX, D11AH, J06BB, L01XC, L04AA-C, M05BX, R03DX | Molecular targeted agents |
| 111 | Molecular targeted drugs (Small molecules) | L01EA-D, L01EG, L01EH, L01EJ, L01EK, L01EX, L01XG, L01XK, L04AA |  |
| 112 | Aminosteroids | M03AC | Muscle relaxants |
| 113 | Centrally acting muscle relaxants | M03BA, M03BX |  |
| 114 | Peripherally acting muscle relaxants | M03AB, M03AX |  |
| 115 | Anabolic androgenic steroids | A14AA | Others |
| 116 | Anticholinesterases | N07AA |  |
| 117 | Antiemetics | A04AD |  |
| 118 | Drugs for neural intractable disease | L03AX, N07AB, N07AX, N07XX |  |
| 119 | Hematopoietic agents | B03XA, L03AA |  |
| 120 | Hemostatic agents | B02BD, B02BX, C05BB |  |
| 121 | Kampo medicines | Not applicable |  |
| 122 | Uterotonics | G02AD |  |
| 123 | Vitamins | A11CA, A11DA, A11GA, A11HA, B02BA, B03BA, B03BB, C04AC, C10AD, D05BB, D10AD |  |
| 124 | Expectorants and mucolytics | R05CA, R05CB | Respiratory agents |
| 125 | Glucocorticoids, inhalants | R03BA |  |
| 126 | Non-selective β-adrenergic receptor stimulators | R03CA, R03CB |  |
| 127 | Opioid alkaloids and derivatives | R05DA |  |
| 128 | Peripherally acting anticholinergic drugs | R03BB |  |
| 129 | Respiratory stimulants | R07AB, V03AB |  |
| 130 | Selective β2 adrenoreceptor agonists | R03AC |  |
| 131 | Xanthine derivatives | R03DA, V04CG |  |
| 132 | Other cough suppresants (not opioids) | R05DB |  |
| 133 | Antithyroid drugs | H03BA, H03BB | Thiroid disease agents |
| 134 | Drugs for treatment of hypothyroidism | H03AA |  |

909 different drugs that were not frequently used in combination with other drugs in eligible patients were classified into 134 therapeutic classes as drugs with the same indications after being classified into the 4^th^ level of the Anatomical Therapeutic Classification (ATC).

**Supplementary Table S3. Optimized booster parameters in extreme gradient boosting (XGBoost) tree model.**

| **Hyperparameters** | **Default** | **Optimized** |
| --- | --- | --- |
| General parameters |  |  |
| ‘booster’ | ‘gbtree’ | ‘gbtree’ |
| Booster parameters |  |  |
| ‘eta’ | 0.3 | 0.1 |
| ‘max_depth’ | 6 | 10 |
| ‘min_child_weight’ | 1 | 1 |
| ‘gamma’ | 0 | 0 |
| ‘colsample_bytree’ | 1 | 1 |
| ‘subsample’ | 1 | 0.8 |
| Learning task parameters |  |  |
| ‘objective’ | ‘binary: logistic’ | ‘binary: logistic’ |
| ‘eval_metric’ | ‘error’ | ‘error’ |
| Evaluation metrics |  |  |
| *In training set* |  |  |
| Misclassification error rate | 0.047 | 0.035 |
| *In testing set* |  |  |
| AUROC [95%CI] | 0.844 [0.832-0.856] | 0.858 [0.847-0.869] |
| AUPR | 0.435 | 0.448 |

Booster parameters:

eta, learning rate;

max_depth, maximum depth of a tree;

min_child_weight, minimum sum of instance weight needed in a child;

gamma, minimum loss reduction required to make a further partition on a leaf node of the tree;

colsample_bytree, subsample ratio of columns when constructing each tree;

subsample, subsample ratio of training instance.

Steps in parameter tuning are as follows:

1) Learning rate (‘eta’, default=0.3) was fixed at 0.1.

2) Ten-fold cross-validation for the training set was performed using the eta and other default parameters to determine the best iterations (‘nrounds’, set at 343).

3) Grid search was performed with fixed learning rate and the best iterations to determine the rest of the hyperparameters minimizing misclassification error rate for the training set.

Search space is as follows:

‘max_depth’: {2, 4, 6, 8, 10}

‘min_child_weight’: {ranged from 1 to 20 by 1}

‘gamma’: {ranged from 0 to 20 by 1}

‘colsample_bytree’: {ranged from 0.5 to 1.0 by 0.1}

‘subsample’: {ranged from 0.5 to 1.0 by 0.1}

Abbreviations: AUPR, area under precision-recall curve; AUROC, area under receiver operating characteristic curve; CI, confidence interval.

**Supplementary Table S4. Comparison of patients’ characteristics of case group between training and testing sets.**

| **Characteristics** | **Training set (N=2,916)** | **Testing set (N=1,236)** | **P value** |
| --- | --- | --- | --- |
| *Demographic information* |  |  |  |
| Age (years), mean (SD) | 63.8 (16.7) | 63.7 (16.3) | 0.789 |
| Male, n (%) | 1,875 (64.3) | 764 (61.8) | 0.137 |
| Number of concomitant drugs, median (IQR) | 7.0 (4.0-10.0) | 7.0 (4.0-10.0) | 0.524 |
| Hospital, n (%) |  |  | 0.717 |
| Itabashi | 2,411 (82.7) | 1,014 (82.0) |  |
| Hikarigaoka | 261 (9.0) | 109 (8.8) |  |
| Surugadai | 244 (8.4) | 113 (9.1) |  |
| *Medical history, n (%)* |  |  |  |
| Hypertension | 834 (28.6) | 369 (29.9) | 0.437 |
| Diabetes | 986 (33.8) | 426 (34.5) | 0.711 |
| Dyslipidemia | 518 (17.8) | 219 (17.7) | 1.000 |
| Heart failure | 603 (20.7) | 268 (21.7) | 0.494 |
| Sepsis | 217 (7.4) | 102 (8.3) | 0.405 |
| *Liver function tests, median (IQR)* |  |  |  |
| ALT (U/L) | 181.0 (149.0-275.0) | 186.0 (150.4-279.0) | 0.333 |
| AST (U/L) | 170.0 (104.0-343.1) | 173.0 (106.8-335.0) | 0.575 |
| TBL (mg/dL) | 0.8 (0.5-1.4) | 0.8 (0.5-1.5) | 0.426 |
| ALP (U/L) | 357.0 (238.0-610.2) | 369.5 (239.0-633.8) | 0.167 |

Unpaired 2-tailed Welch’s t-test or Wilcoxon rank-sum test for continuous data and chi-squared test for categorical data were performed. Abbreviations: ALP, alkaline phosphatase; ALT, alanine aminotransferase; AST, aspartate aminotransferase; IQR, interquartile range; SD, standard deviation; TBL, total bilirubin; U/L, units per liter.

**Supplementary Table S5. Comparison of patients’ characteristics of control group between training and testing sets.**

| **Characteristics** | **Training set (N=37,973)** | **Testing set (N=16,288)** | **P value** |
| --- | --- | --- | --- |
| *Demographic information* |  |  |  |
| Age (years), mean (SD) | 57.0 (19.6) | 26.9 (19.7) | 0.636 |
| Male, n (%) | 15,364 (40.5) | 6,577 (40.4) | 0.868 |
| Number of concomitant drugs, median (IQR) | 4.0 (2.0-6.0) | 4.0 (2.0-6.0) | 0.431 |
| Hospital, n (%) |  |  | 0.751 |
| Itabashi | 29,622 (78.0) | 12,689 (77.9) |  |
| Hikarigaoka | 4,250 (11.2) | 1,857 (11.4) |  |
| Surugadai | 4,101 (10.8) | 1742 (10.7) |  |
| *Medical history, n (%)* |  |  |  |
| Hypertension | 6,622 (17.4) | 2,777 (17.0) | 0.277 |
| Diabetes | 10,586 (27.9) | 4,449 (27.3) | 0.183 |
| Dyslipidemia | 4,840 (12.7) | 2090 (12.8) | 0.795 |
| Heart failure | 3,917 (10.3) | 1,706 (10.5) | 0.589 |
| Sepsis | 423 (1.1) | 193 (1.2) | 0.502 |
| *Liver function tests, median (IQR)* |  |  |  |
| ALT (U/L) | 14.0 (11.0-20.0) | 14.0 (11.0-20.0) | 0.195 |
| AST (U/L) | 19.0 (15.0-23.0) | 19.0 (15.0-23.0) | 0.863 |
| TBL (mg/dL) | 0.5 (0.4-0.7) | 0.5 (0.4-0.7) | 0.655 |
| ALP (U/L) | 209.0 (165.0-264.0) | 209.0 (166.0-265.0) | 0.573 |

Unpaired 2-tailed Welch’s t-test or Wilcoxon rank-sum test for continuous data and chi-squared test for categorical data were performed. Abbreviations: ALP, alkaline phosphatase; ALT, alanine aminotransferase; AST, aspartate aminotransferase; IQR, interquartile range; SD, standard deviation; TBL, total bilirubin; U/L, units per liter.

**Supplementary Table S6. Comparison of top 100 regression coefficients between multiple logistic regression and logistic least absolute shrinkage and selection operator (LASSO) regression models.**

| **MLR** | |  | **Logistic LASSO regression** | |
| --- | --- | --- | --- | --- |
| **Feature** | **Estimated coefficient** |  | **Feature** | **Estimated coefficient** |
| Epirubicin * Ranitidine | 38.086 |  | Carbapenems [TC] | 1.660 |
| Diastase * Pancreatin | 31.068 |  | Cyclophosphamide * Palonosetron | -1.558 |
| Ethambutol * Rifampicin | 30.595 |  | Fentanyl | 1.394 |
| Doxorubicin * Prednisolone | -30.570 |  | Cefazolin * Sennoside | 1.107 |
| Vincristine | 26.218 |  | Phenytoin | 1.046 |
| Cyclophosphamide | -23.161 |  | Methylergometrine | -0.999 |
| Celecoxib * Tramadol | -21.985 |  | Anabolic androgenic steroids [TC] | 0.970 |
| Cyclophosphamide * Ranitidine | -20.218 |  | Drugs for treatment of ischemic stroke [TC] | 0.956 |
| Epirubicin | -17.647 |  | Insulin (human) | 0.921 |
| Benserazide | -17.570 |  | Loxoprofen * Warfarin | 0.915 |
| Antihypertensives for pulmonary arterial hypertension [TC] | -17.229 |  | Sepsis | 0.914 |
| Goserelin * Bicalutamide | 16.785 |  | Insulin (human) * L-carbocisteine | -0.908 |
| Cyclophosphamide * Palonosetron | -16.775 |  | Doxazosin * Furosemide | -0.893 |
| Rifampicin | -16.726 |  | Trichlormethiazide | -0.869 |
| Remifentanil | -16.586 |  | Glycopeptides [TC] | 0.869 |
| Diastase | -16.516 |  | Budesonide | -0.861 |
| Anti-androgens [TC] | -16.469 |  | Polyene macrolide antibiotics [TC] | 0.813 |
| Antiretroviral drugs [TC] | -16.449 |  | Acetaminophen * Rosuvastatin | 0.808 |
| Benserazide * Levodopa | 16.425 |  | Tranexamic acid | -0.807 |
| Goserelin | -16.327 |  | Celecoxib * Sennoside | -0.793 |
| Fentanyl * Remifentanil | 16.220 |  | Dexamethasone sodium phosphate | 0.777 |
| Molecular targeted drugs (Small molecules) [TC] | -16.094 |  | Tazobactam/piperacillin | 0.771 |
| Ethambutol | -15.926 |  | Other sexhormones [TC] | 0.759 |
| Drugs used for Cushing's syndrome [TC] | -15.922 |  | Azelnidipine * Olmesartan | -0.758 |
| Isoniazid * Ethambutol | -15.904 |  | Sex (male) | 0.745 |
| Amoxicillin * Loxoprofen | -15.673 |  | Other hormone preparations [TC] | 0.743 |
| Isoniazid * Rifampicin | 15.619 |  | Fibrates [TC] | -0.741 |
| Glucagon-like peptide-1 (GLP-1) analogues [TC] | -15.500 |  | Itopride | 0.731 |
| Salmeterol * Fluticasone | 15.329 |  | Ergot alkaloids [TC] | -0.721 |
| Interferons [TC] | -15.318 |  | Drugs for treatment of chronic kidney disease [TC] | -0.707 |
| Azelnidipine * Olmesartan | -15.304 |  | Diclofenac * Famotidine | 0.706 |
| Angiotensin receptor blockers (ARBs) [TC] | -15.278 |  | Metoclopramide * Remifentanil | -0.703 |
| Salmeterol | -15.200 |  | Prednisolone * Heparin sodium | 0.702 |
| Antiparasitic drugs [TC] | -15.164 |  | Celecoxib | -0.697 |
| Formoterol | -14.942 |  | Famotidine * Polaprezinc | -0.696 |
| Budesonide * Formoterol | 14.681 |  | Metformin | -0.693 |
| gonadotropin releasing hormone analogues [TC] | -14.621 |  | Heparin sodium * Furosemide | 0.690 |
| Gonadotropins [TC] | -14.472 |  | Alfacalcidol | -0.687 |
| Anterior pituitary lobe hormones and analogues [TC] | -14.438 |  | Dipeptidyl peptidase 4 (DPP-4) inhibitors [TC] | -0.678 |
| Olmesartan * Benidipine | -14.227 |  | Aspirin * Tamsulosin | -0.673 |
| Pancreatin | -14.107 |  | Lornoxicam | -0.658 |
| Aprepitant * Cyclophosphamide | -13.911 |  | Butyrophenone derivatives [TC] | 0.652 |
| Vincristine * Prednisolone | 13.851 |  | Aspirin * Senna | -0.650 |
| Cyclophosphamide * Doxorubicin | 13.819 |  | Drugs for constipation [TC] | -0.643 |
| Prednisolone * Celecoxib | -13.644 |  | Diclofenac * Misoprostol | -0.643 |
| Cefotiam * Teprenone | -12.865 |  | Sulbactam/ampicillin | 0.636 |
| Epirubicin * Cyclophosphamide | 12.292 |  | Cefmetazole | 0.626 |
| Methylergometrine * Magnesium oxide | -12.083 |  | Dexamethasone sodium phosphate * Magnesium oxide | -0.626 |
| Cefaclor * Methylergometrine | -10.537 |  | Drugs for treatment of Alzheimer's disease [TC] | -0.621 |
| Serrapeptase * Methylergometrine | -10.518 |  | Itopride * Polaprezinc | 0.611 |

**Supplementary Table S6. Continued.**

| **MLR** | |  | **Logistic LASSO regression** | |
| --- | --- | --- | --- | --- |
| **Feature** | **Estimated coefficient** |  | **Feature** | **Estimated coefficient** |
| Cefdinir * Methylprednisolone | -7.366 |  | Noradrenalin * Fentanyl | -0.607 |
| Prasugrel | -5.384 |  | Ramosetron * Dexamethasone sodium phosphate | -0.606 |
| Aspirin * Prasugrel | 5.009 |  | Drugs for urinary frequency and incontinence [TC] | -0.604 |
| Isoniazid * Pyrazinamide | -4.763 |  | Kallidinogenase | -0.604 |
| Sitagliptin * Metformin | -4.622 |  | Eperisone | -0.601 |
| Paclitaxel | 4.597 |  | Trimethoprim/sulfamethoxazole | 0.601 |
| Alfacalcidol * Sennoside | -4.322 |  | Nifedipine * Furosemide | -0.595 |
| Hydrocortisone * Rebamipide | -4.250 |  | Diphenhydramine | 0.581 |
| Tranexamic acid * Haloperidol | -3.792 |  | General anesthetics [TC] | 0.580 |
| Celecoxib * Fondaparinux | 3.715 |  | Centrally acting anticholinergic drugs [TC] | -0.570 |
| Paclitaxel * Dexamethasone sodium phosphate | -3.708 |  | Furosemide * Ambroxol | -0.557 |
| Celecoxib * Sennoside | -3.606 |  | Respiratory stimulants [TC] | 0.553 |
| Methylprednisolone * Loxoprofen | 3.571 |  | Loxoprofen * Ranitidine | -0.547 |
| Aspirin * Febuxostat | -3.094 |  | Acetaminophen * Salicylamide | -0.540 |
| Palonosetron * Chlorpheniramine | 3.023 |  | Sitagliptin | -0.528 |
| Other sexhormones [TC] | 3.011 |  | Hydroxyzine * Famotidine | -0.527 |
| Olopatadine * Carbazochrome | 3.001 |  | Sulbactam/ampicillin * Clindamycin | -0.527 |
| Metoclopramide * Buprenorphine | -2.982 |  | Azelnidipine | -0.513 |
| Doxazosin * Furosemide | -2.974 |  | Furosemide * Brotizolam | -0.513 |
| Granisetron * Cyclophosphamide | -2.847 |  | Antihyperuricemic drugs [TC] | -0.512 |
| Clindamycin * Metoclopramide | -2.799 |  | Bicalutamide * Leuprorelin | -0.512 |
| Olopatadine * Teprenone | -2.782 |  | Heparin sodium * Omeprazole | 0.508 |
| Budesonide | -2.727 |  | Telmisartan | -0.505 |
| Rabeprazole * Adenosine triphosphate | -2.703 |  | Amlodipine * Nicorandil | -0.504 |
| Leukotriene receptor antagonists [TC] | -2.687 |  | Teprenone | -0.503 |
| Loxoprofen * Rosuvastatin | 2.678 |  | Cefdinir | -0.500 |
| Pregabalin * Magnesium oxide | 2.622 |  | Senna * Fentanyl | 0.497 |
| Azasetron * Aprepitant | 2.595 |  | Famotidine | 0.497 |
| Carbazochrome * Metoclopramide | -2.582 |  | Histamine (H1) receptor antagonists [TC] | -0.493 |
| Bicalutamide * Leuprorelin | -2.542 |  | Fentanyl * Midazolam | 0.492 |
| Ramosetron * Dexamethasone sodium phosphate | -2.497 |  | Aspirin * Edaravone | -0.490 |
| Carboplatin | -2.490 |  | Cefaclor | -0.485 |
| Carbazochrome * Haloperidol | 2.480 |  | Hydrocortisone * Rebamipide | -0.481 |
| Betamethasone * Chlorpheniramine | -2.478 |  | Celecoxib * Tramadol | -0.474 |
| Carbazochrome * Heparin sodium | 2.464 |  | Aspirin * Sennoside | -0.473 |
| Cefaclor * Serrapeptase | 2.443 |  | Febuxostat | -0.468 |
| Cefmetazole * Heparin sodium | -2.412 |  | Loxoprofen * Brotizolam | 0.468 |
| Acetaminophen * Rosuvastatin | 2.386 |  | Leukotriene receptor antagonists [TC] | -0.468 |
| Trichlormethiazide | -2.373 |  | Aspirin * Diazepam | 0.466 |
| Acetaminophen * Fexofenadine | -2.368 |  | Cyclophosphamide * Dexamethasone sodium phosphate | -0.466 |
| Rosuvastatin * Rebamipide | -2.358 |  | Pranlukast | -0.466 |
| Clindamycin * Haloperidol | 2.347 |  | Tetracyclic antidepressants [TC] | -0.463 |
| Ranitidine * Rabeprazole | -2.346 |  | Hydrocortisone * Heparin sodium | -0.462 |
| Amlodipine * Nicorandil | -2.335 |  | Clopidogrel * Nifedipine | -0.460 |
| Kallidinogenase | -2.326 |  | Levodopa | -0.460 |
| Methylergometrine | -2.311 |  | Rabeprazole * Adenosine triphosphate | -0.456 |
| Lansoprazole * Etizolam | -2.289 |  | Celecoxib * Famotidine | -0.454 |
| Buprenorphine * Pentazocine | -2.251 |  | Etodolac | -0.449 |
| Acetaminophen * Buprenorphine | 2.242 |  | Cephalosporins [TC] | 0.443 |
| Teprenone * Midazolam | -2.235 |  | Cefdinir * Methylprednisolone | -0.436 |

Regression coefficients were sorted by absolute magnitude in descending order. * indicates product term of two drugs. Abbreviations: LASSO, least absolute shrinkage and selection operator; MLR, multiple logistic regression; TC, therapeutic class.

**Supplementary Table S7. Synergistic interaction between two drugs on risk of abnormal elevation of serum alanine amino transferase.**

| Drug 1 | Drug 2 | Adjusted odds ratio | | | | | | | | | | |  | Synergistic interaction | | |
| --- | --- | --- | --- | --- | --- | --- | --- | --- | --- | --- | --- | --- | --- | --- | --- | --- |
|  |  | Drug 1 | | |  | Drug 2 | | |  | Product term (Drug 1*Drug 2) | | |  |  |  |  |
|  |  | Estimate | 95%BootCI | |  | Estimate | 95%BootCI | |  | Estimate | 95%BootCI | |  | RERI | 95%BootCI | |
|  |  |  | Lower | Upper |  |  | Lower | Upper |  |  | Lower | Upper |  |  | Lower | Upper |
| Trimethoprim/sulfamethoxazole | Prednisolone | 1.823 | 1.440 | 5.764 |  | 1.000 | 0.950 | 1.606 |  | 1.000 | 0.380 | 1.726 |  | 0.000 | -3.100 | 1.728 |
| Trimethoprim/sulfamethoxazole | Lansoprazole | 1.823 | 1.440 | 5.764 |  | 1.084 | 1.000 | 1.520 |  | 0.673 | 0.079 | 0.834 |  | -0.577 | -4.716 | -0.262 |
| Azithromycin | Ambroxol | 1.221 | 0.849 | 2.663 |  | 1.000 | 0.702 | 1.258 |  | 1.000 | 0.494 | 2.291 |  | 0.000 | -1.047 | 1.456 |
| Azithromycin | L-carbocisteine | 1.221 | 0.849 | 2.663 |  | 1.000 | 0.595 | 1.000 |  | 1.000 | 0.255 | 1.117 |  | 0.000 | -1.763 | 0.083 |
| Amoxicillin | Clarithromycin | 0.662 | 0.235 | 1.000 |  | 0.698 | 0.344 | 0.977 |  | 1.000 | 0.266 | 2.560 |  | 0.102 | -0.412 | 0.604 |
| Amoxicillin | Acetaminophen | 0.662 | 0.235 | 1.000 |  | 1.000 | 1.000 | 1.518 |  | 1.000 | 0.224 | 3.173 |  | 0.000 | -0.750 | 1.238 |
| Amoxicillin | Loxoprofen | 0.662 | 0.235 | 1.000 |  | 0.824 | 0.611 | 1.000 |  | 0.942 | 0.080 | 1.000 |  | 0.028 | -0.696 | 0.200 |
| Amoxicillin | Tranexamic acid | 0.662 | 0.235 | 1.000 |  | 0.446 | 0.263 | 0.796 |  | 1.000 | 0.238 | 1.336 |  | 0.187 | -0.261 | 0.468 |
| Amoxicillin | L-carbocisteine | 0.662 | 0.235 | 1.000 |  | 1.000 | 0.595 | 1.000 |  | 1.000 | 0.224 | 2.470 |  | 0.000 | -0.577 | 0.694 |
| Amoxicillin | Rebamipide | 0.662 | 0.235 | 1.000 |  | 0.739 | 0.345 | 0.760 |  | 1.000 | 0.173 | 1.000 |  | 0.088 | -0.382 | 0.397 |
| Sulbactam/ampicillin | Clindamycin | 1.889 | 1.467 | 3.798 |  | 1.000 | 0.875 | 2.117 |  | 0.591 | 0.118 | 1.000 |  | -0.773 | -2.875 | 0.300 |
| Sulbactam/ampicillin | Acetaminophen | 1.889 | 1.467 | 3.798 |  | 1.000 | 1.000 | 1.518 |  | 1.000 | 0.391 | 1.348 |  | 0.000 | -1.711 | 1.321 |
| Sulbactam/ampicillin | Loxoprofen | 1.889 | 1.467 | 3.798 |  | 0.824 | 0.611 | 1.000 |  | 1.000 | 0.571 | 2.038 |  | -0.157 | -1.279 | 2.124 |
| Sulbactam/ampicillin | Hydroxyzine | 1.889 | 1.467 | 3.798 |  | 1.000 | 0.545 | 1.272 |  | 1.000 | 0.479 | 4.161 |  | 0.000 | -1.452 | 7.496 |
| Sulbactam/ampicillin | Insulin (human) | 1.889 | 1.467 | 3.798 |  | 2.511 | 1.398 | 3.931 |  | 1.000 | 0.565 | 3.087 |  | 1.343 | -0.515 | 16.019 |
| Sulbactam/ampicillin | Carbazochrome | 1.889 | 1.467 | 3.798 |  | 1.000 | 0.377 | 1.000 |  | 1.000 | 0.251 | 2.012 |  | 0.000 | -2.177 | 1.384 |
| Sulbactam/ampicillin | Tranexamic acid | 1.889 | 1.467 | 3.798 |  | 0.446 | 0.263 | 0.796 |  | 1.000 | 0.381 | 1.981 |  | -0.492 | -1.975 | 0.279 |
| Sulbactam/ampicillin | Heparin sodium | 1.889 | 1.467 | 3.798 |  | 1.000 | 1.000 | 1.740 |  | 0.803 | 0.260 | 1.000 |  | -0.371 | -2.490 | 0.135 |
| Sulbactam/ampicillin | Amlodipine | 1.889 | 1.467 | 3.798 |  | 0.797 | 0.467 | 0.994 |  | 1.523 | 1.000 | 4.986 |  | 0.606 | -0.580 | 6.232 |
| Sulbactam/ampicillin | Ambroxol | 1.889 | 1.467 | 3.798 |  | 1.000 | 0.702 | 1.258 |  | 0.652 | 0.177 | 1.214 |  | -0.657 | -2.440 | 0.569 |
| Sulbactam/ampicillin | L-carbocisteine | 1.889 | 1.467 | 3.798 |  | 1.000 | 0.595 | 1.000 |  | 1.000 | 0.489 | 1.999 |  | 0.000 | -1.550 | 1.972 |
| Sulbactam/ampicillin | Metoclopramide | 1.889 | 1.467 | 3.798 |  | 1.000 | 0.765 | 1.290 |  | 1.000 | 0.309 | 1.730 |  | 0.000 | -1.954 | 1.725 |
| Sulbactam/ampicillin | Mosapride | 1.889 | 1.467 | 3.798 |  | 1.000 | 0.620 | 1.251 |  | 1.000 | 0.296 | 2.148 |  | 0.000 | -1.903 | 2.574 |
| Sulbactam/ampicillin | Famotidine | 1.889 | 1.467 | 3.798 |  | 1.643 | 1.332 | 2.071 |  | 0.820 | 0.453 | 1.269 |  | 0.012 | -1.387 | 2.026 |
| Sulbactam/ampicillin | Lansoprazole | 1.889 | 1.467 | 3.798 |  | 1.084 | 1.000 | 1.520 |  | 1.339 | 0.921 | 3.572 |  | 0.768 | 0.000 | 7.738 |
| Sulbactam/ampicillin | Rebamipide | 1.889 | 1.467 | 3.798 |  | 0.739 | 0.345 | 0.760 |  | 1.000 | 0.473 | 2.678 |  | -0.232 | -1.649 | 1.588 |
| Sulbactam/ampicillin | Magnesium oxide | 1.889 | 1.467 | 3.798 |  | 0.949 | 0.749 | 1.096 |  | 1.000 | 0.298 | 1.602 |  | -0.045 | -2.087 | 1.173 |
| Sulbactam/ampicillin | Haloperidol | 1.889 | 1.467 | 3.798 |  | 1.000 | 0.447 | 1.024 |  | 0.899 | 0.273 | 1.743 |  | -0.191 | -2.061 | 1.494 |
| Sulbactam/ampicillin | Fentanyl | 1.889 | 1.467 | 3.798 |  | 4.031 | 3.726 | 8.961 |  | 0.802 | 0.164 | 1.000 |  | 1.189 | -5.694 | 6.455 |
| Isoniazid | Ethambutol | 1.000 | 1.000 | 4.834 |  | 1.000 | 0.483 | 1.671 |  | 1.000 | 0.103 | 1.000 |  | 0.000 | -3.528 | 0.000 |
| Isoniazid | Pyrazinamide | 1.000 | 1.000 | 4.834 |  | 1.707 | 1.000 | 7.624 |  | 1.000 | 0.558 | 4.581 |  | 0.000 | -0.880 | 13.463 |
| Isoniazid | Rifampicin | 1.000 | 1.000 | 4.834 |  | 1.000 | 0.376 | 1.153 |  | 1.000 | 0.276 | 1.138 |  | 0.000 | -3.278 | 0.273 |
| Ethambutol | Pyrazinamide | 1.000 | 0.483 | 1.671 |  | 1.707 | 1.000 | 7.624 |  | 1.000 | 0.542 | 3.059 |  | 0.000 | -1.632 | 5.385 |
| Ethambutol | Rifampicin | 1.000 | 0.483 | 1.671 |  | 1.000 | 0.376 | 1.153 |  | 1.000 | 0.439 | 1.105 |  | 0.000 | -0.467 | 0.263 |
| Clarithromycin | Acetaminophen | 0.698 | 0.344 | 0.977 |  | 1.000 | 1.000 | 1.518 |  | 1.000 | 0.834 | 3.932 |  | 0.000 | -0.253 | 1.803 |
| Clarithromycin | Loxoprofen | 0.698 | 0.344 | 0.977 |  | 0.824 | 0.611 | 1.000 |  | 1.000 | 0.439 | 4.419 |  | 0.053 | -0.247 | 1.656 |
| Clarithromycin | Tranexamic acid | 0.698 | 0.344 | 0.977 |  | 0.446 | 0.263 | 0.796 |  | 1.000 | 0.158 | 1.988 |  | 0.167 | -0.246 | 0.499 |
| Clarithromycin | Heparin sodium | 0.698 | 0.344 | 0.977 |  | 1.000 | 1.000 | 1.740 |  | 1.000 | 0.694 | 3.773 |  | 0.000 | -0.420 | 1.953 |
| Clarithromycin | Ambroxol | 0.698 | 0.344 | 0.977 |  | 1.000 | 0.702 | 1.258 |  | 1.000 | 0.287 | 1.780 |  | 0.000 | -0.577 | 0.407 |
| Clarithromycin | L-carbocisteine | 0.698 | 0.344 | 0.977 |  | 1.000 | 0.595 | 1.000 |  | 1.000 | 0.517 | 1.584 |  | 0.000 | -0.359 | 0.319 |
| Clarithromycin | Lansoprazole | 0.698 | 0.344 | 0.977 |  | 1.084 | 1.000 | 1.520 |  | 1.000 | 0.310 | 1.632 |  | -0.025 | -0.713 | 0.332 |
| Clarithromycin | Rebamipide | 0.698 | 0.344 | 0.977 |  | 0.739 | 0.345 | 0.760 |  | 1.000 | 0.365 | 1.534 |  | 0.079 | -0.183 | 0.421 |
| Clindamycin | Cefditoren pivoxil | 1.000 | 0.875 | 2.117 |  | 0.882 | 0.410 | 1.046 |  | 1.000 | 0.432 | 3.939 |  | 0.000 | -0.720 | 2.771 |
| Clindamycin | Hydrocortisone | 1.000 | 0.875 | 2.117 |  | 1.089 | 1.000 | 3.856 |  | 1.000 | 0.047 | 1.000 |  | 0.000 | -3.382 | 0.076 |
| Clindamycin | Acetaminophen | 1.000 | 0.875 | 2.117 |  | 1.000 | 1.000 | 1.518 |  | 0.842 | 0.150 | 1.000 |  | -0.158 | -1.553 | 0.091 |
| Clindamycin | Loxoprofen | 1.000 | 0.875 | 2.117 |  | 0.824 | 0.611 | 1.000 |  | 1.000 | 0.365 | 1.358 |  | 0.000 | -0.876 | 0.331 |

**Supplementary Table S7. Continued.**

| Drug 1 | Drug 2 | Adjusted odds ratio | | | | | | | | | | |  | Synergistic interaction | | |
| --- | --- | --- | --- | --- | --- | --- | --- | --- | --- | --- | --- | --- | --- | --- | --- | --- |
|  |  | Drug 1 | | |  | Drug 2 | | |  | Product term (Drug 1*Drug 2) | | |  |  |  |  |
|  |  | Estimate | 95%BootCI | |  | Estimate | 95%BootCI | |  | Estimate | 95%BootCI | |  | RERI | 95%BootCI | |
|  |  |  | Lower | Upper |  |  | Lower | Upper |  |  | Lower | Upper |  |  | Lower | Upper |
| Clindamycin | Tranexamic acid | 1.000 | 0.875 | 2.117 |  | 0.446 | 0.263 | 0.796 |  | 1.000 | 0.981 | 3.212 |  | 0.000 | -0.536 | 1.094 |
| Clindamycin | Heparin sodium | 1.000 | 0.875 | 2.117 |  | 1.000 | 1.000 | 1.740 |  | 1.000 | 0.261 | 1.103 |  | 0.000 | -1.307 | 0.312 |
| Clindamycin | L-carbocisteine | 1.000 | 0.875 | 2.117 |  | 1.000 | 0.595 | 1.000 |  | 1.000 | 0.195 | 1.073 |  | 0.000 | -1.296 | 0.074 |
| Clindamycin | Metoclopramide | 1.000 | 0.875 | 2.117 |  | 1.000 | 0.765 | 1.290 |  | 0.903 | 0.082 | 2.700 |  | -0.097 | -1.318 | 2.368 |
| Clindamycin | Famotidine | 1.000 | 0.875 | 2.117 |  | 1.643 | 1.332 | 2.071 |  | 1.000 | 0.308 | 1.688 |  | 0.000 | -1.285 | 1.641 |
| Clindamycin | Rebamipide | 1.000 | 0.875 | 2.117 |  | 0.739 | 0.345 | 0.760 |  | 1.000 | 0.612 | 2.626 |  | 0.000 | -0.630 | 0.768 |
| Clindamycin | Haloperidol | 1.000 | 0.875 | 2.117 |  | 1.000 | 0.447 | 1.024 |  | 1.000 | 0.141 | 2.106 |  | 0.000 | -1.171 | 0.853 |
| Cefaclor | Acetaminophen | 0.616 | 0.208 | 0.786 |  | 1.000 | 1.000 | 1.518 |  | 1.000 | 0.459 | 4.181 |  | 0.000 | -0.410 | 1.266 |
| Cefaclor | Diclofenac | 0.616 | 0.208 | 0.786 |  | 1.319 | 1.189 | 2.821 |  | 1.000 | 0.324 | 3.429 |  | -0.054 | -1.386 | 1.186 |
| Cefaclor | Serrapeptase | 0.616 | 0.208 | 0.786 |  | 1.000 | 0.339 | 1.000 |  | 1.000 | 1.000 | 9.337 |  | 0.000 | 0.000 | 2.155 |
| Cefaclor | Loxoprofen | 0.616 | 0.208 | 0.786 |  | 0.824 | 0.611 | 1.000 |  | 1.000 | 0.458 | 2.540 |  | 0.068 | -0.213 | 0.590 |
| Cefaclor | Methylergometrine | 0.616 | 0.208 | 0.786 |  | 0.368 | 0.080 | 0.364 |  | 1.000 | 0.688 | 1.000 |  | 0.243 | 0.153 | 0.664 |
| Cefaclor | Heparin sodium | 0.616 | 0.208 | 0.786 |  | 1.000 | 1.000 | 1.740 |  | 1.000 | 1.000 | 6.210 |  | 0.000 | -0.328 | 2.138 |
| Cefaclor | Teprenone | 0.616 | 0.208 | 0.786 |  | 0.605 | 0.480 | 1.000 |  | 1.000 | 0.250 | 1.174 |  | 0.152 | -0.234 | 0.355 |
| Cefaclor | Rebamipide | 0.616 | 0.208 | 0.786 |  | 0.739 | 0.345 | 0.760 |  | 1.000 | 0.103 | 1.000 |  | 0.100 | -0.190 | 0.426 |
| Cefaclor | Magnesium oxide | 0.616 | 0.208 | 0.786 |  | 0.949 | 0.749 | 1.096 |  | 1.000 | 0.364 | 4.394 |  | 0.019 | -0.268 | 1.493 |
| Cefazolin | Cefdinir | 1.000 | 1.000 | 2.319 |  | 0.606 | 0.299 | 0.874 |  | 1.000 | 0.119 | 1.603 |  | 0.000 | -1.251 | 0.300 |
| Cefazolin | Acetaminophen | 1.000 | 1.000 | 2.319 |  | 1.000 | 1.000 | 1.518 |  | 1.000 | 0.438 | 1.357 |  | 0.000 | -1.014 | 0.669 |
| Cefazolin | Diclofenac | 1.000 | 1.000 | 2.319 |  | 1.319 | 1.189 | 2.821 |  | 1.000 | 0.180 | 1.000 |  | 0.000 | -2.314 | 0.376 |
| Cefazolin | Celecoxib | 1.000 | 1.000 | 2.319 |  | 0.498 | 0.232 | 0.796 |  | 1.000 | 0.612 | 2.739 |  | 0.000 | -0.804 | 0.717 |
| Cefazolin | Loxoprofen | 1.000 | 1.000 | 2.319 |  | 0.824 | 0.611 | 1.000 |  | 1.000 | 0.505 | 1.154 |  | 0.000 | -0.873 | 0.126 |
| Cefazolin | Hydroxyzine | 1.000 | 1.000 | 2.319 |  | 1.000 | 0.545 | 1.272 |  | 1.000 | 0.154 | 1.000 |  | 0.000 | -1.651 | 0.000 |
| Cefazolin | Carbazochrome | 1.000 | 1.000 | 2.319 |  | 1.000 | 0.377 | 1.000 |  | 1.000 | 1.000 | 3.670 |  | 0.000 | -0.438 | 2.633 |
| Cefazolin | Tranexamic acid | 1.000 | 1.000 | 2.319 |  | 0.446 | 0.263 | 0.796 |  | 0.711 | 0.137 | 1.000 |  | -0.129 | -1.478 | -0.042 |
| Cefazolin | Heparin sodium | 1.000 | 1.000 | 2.319 |  | 1.000 | 1.000 | 1.740 |  | 0.956 | 0.313 | 1.000 |  | -0.044 | -1.595 | 0.002 |
| Cefazolin | Amlodipine | 1.000 | 1.000 | 2.319 |  | 0.797 | 0.467 | 0.994 |  | 1.000 | 0.555 | 2.538 |  | 0.000 | -0.779 | 1.402 |
| Cefazolin | Metoclopramide | 1.000 | 1.000 | 2.319 |  | 1.000 | 0.765 | 1.290 |  | 1.000 | 0.478 | 1.759 |  | 0.000 | -0.844 | 1.094 |
| Cefazolin | Esomeprazole | 1.000 | 1.000 | 2.319 |  | 0.671 | 0.373 | 0.954 |  | 1.000 | 0.294 | 1.419 |  | 0.000 | -1.190 | 0.151 |
| Cefazolin | Teprenone | 1.000 | 1.000 | 2.319 |  | 0.605 | 0.480 | 1.000 |  | 1.000 | 0.160 | 1.000 |  | 0.000 | -1.470 | 0.000 |
| Cefazolin | Famotidine | 1.000 | 1.000 | 2.319 |  | 1.643 | 1.332 | 2.071 |  | 1.000 | 0.365 | 1.017 |  | 0.000 | -1.385 | 0.520 |
| Cefazolin | Misoprostol | 1.000 | 1.000 | 2.319 |  | 1.000 | 0.330 | 1.000 |  | 1.000 | 1.000 | 6.893 |  | 0.000 | -0.338 | 5.153 |
| Cefazolin | Ranitidine | 1.000 | 1.000 | 2.319 |  | 1.155 | 1.015 | 2.030 |  | 1.000 | 0.813 | 2.421 |  | 0.000 | -0.188 | 3.452 |
| Cefazolin | Lansoprazole | 1.000 | 1.000 | 2.319 |  | 1.084 | 1.000 | 1.520 |  | 1.000 | 0.523 | 3.071 |  | 0.000 | -0.783 | 3.599 |
| Cefazolin | Rebamipide | 1.000 | 1.000 | 2.319 |  | 0.739 | 0.345 | 0.760 |  | 1.000 | 0.344 | 1.031 |  | 0.000 | -1.097 | 0.000 |
| Cefazolin | Magnesium oxide | 1.000 | 1.000 | 2.319 |  | 0.949 | 0.749 | 1.096 |  | 1.000 | 0.454 | 2.701 |  | 0.000 | -0.874 | 2.182 |
| Cefazolin | Sennoside | 1.000 | 1.000 | 2.319 |  | 1.000 | 0.852 | 1.372 |  | 3.025 | 1.000 | 4.607 |  | 2.025 | 0.046 | 5.046 |
| Cefazolin | Haloperidol | 1.000 | 1.000 | 2.319 |  | 1.000 | 0.447 | 1.024 |  | 1.000 | 1.000 | 4.293 |  | 0.000 | -0.345 | 4.670 |
| Cefazolin | Fentanyl | 1.000 | 1.000 | 2.319 |  | 4.031 | 3.726 | 8.961 |  | 0.817 | 0.171 | 1.000 |  | -0.736 | -6.178 | 1.887 |
| Cefazolin | Remifentanil | 1.000 | 1.000 | 2.319 |  | 1.000 | 0.473 | 1.000 |  | 1.000 | 0.287 | 1.366 |  | 0.000 | -1.192 | 0.240 |
| Cefazolin | Midazolam | 1.000 | 1.000 | 2.319 |  | 1.000 | 0.814 | 1.575 |  | 1.000 | 0.988 | 2.675 |  | 0.000 | -0.047 | 2.648 |
| Cefotiam | Loxoprofen | 1.000 | 0.402 | 1.109 |  | 0.824 | 0.611 | 1.000 |  | 1.000 | 0.221 | 1.000 |  | 0.000 | -0.621 | 0.147 |
| Cefotiam | Heparin sodium | 1.000 | 0.402 | 1.109 |  | 1.000 | 1.000 | 1.740 |  | 0.791 | 0.211 | 1.000 |  | -0.209 | -1.149 | 0.000 |
| Cefotiam | Teprenone | 1.000 | 0.402 | 1.109 |  | 0.605 | 0.480 | 1.000 |  | 1.000 | 0.205 | 1.000 |  | 0.000 | -0.622 | 0.182 |
| Cefotiam | Ranitidine | 1.000 | 0.402 | 1.109 |  | 1.155 | 1.015 | 2.030 |  | 1.000 | 0.185 | 1.547 |  | 0.000 | -1.189 | 0.685 |
| Cefotiam | Rebamipide | 1.000 | 0.402 | 1.109 |  | 0.739 | 0.345 | 0.760 |  | 1.000 | 0.613 | 3.504 |  | 0.000 | -0.172 | 0.999 |
| Cefcapene pivoxil | Acetaminophen | 0.888 | 0.449 | 1.000 |  | 1.000 | 1.000 | 1.518 |  | 1.000 | 0.363 | 2.448 |  | 0.000 | -0.646 | 1.097 |

**Supplementary Table S7. Continued.**

| Drug 1 | Drug 2 | Adjusted odds ratio | | | | | | | | | | |  | Synergistic interaction | | |
| --- | --- | --- | --- | --- | --- | --- | --- | --- | --- | --- | --- | --- | --- | --- | --- | --- |
|  |  | Drug 1 | | |  | Drug 2 | | |  | Product term (Drug 1*Drug 2) | | |  |  |  |  |
|  |  | Estimate | 95%BootCI | |  | Estimate | 95%BootCI | |  | Estimate | 95%BootCI | |  | RERI | 95%BootCI | |
|  |  |  | Lower | Upper |  |  | Lower | Upper |  |  | Lower | Upper |  |  | Lower | Upper |
| Cefcapene pivoxil | Loxoprofen | 0.888 | 0.449 | 1.000 |  | 0.824 | 0.611 | 1.000 |  | 1.000 | 0.524 | 1.809 |  | 0.020 | -0.312 | 0.502 |
| Cefcapene pivoxil | Tranexamic acid | 0.888 | 0.449 | 1.000 |  | 0.446 | 0.263 | 0.796 |  | 1.000 | 0.217 | 1.993 |  | 0.062 | -0.302 | 0.479 |
| Cefcapene pivoxil | Heparin sodium | 0.888 | 0.449 | 1.000 |  | 1.000 | 1.000 | 1.740 |  | 0.935 | 0.311 | 1.428 |  | -0.058 | -0.839 | 0.297 |
| Cefcapene pivoxil | Teprenone | 0.888 | 0.449 | 1.000 |  | 0.605 | 0.480 | 1.000 |  | 1.000 | 0.184 | 1.246 |  | 0.044 | -0.489 | 0.244 |
| Cefcapene pivoxil | Rebamipide | 0.888 | 0.449 | 1.000 |  | 0.739 | 0.345 | 0.760 |  | 1.000 | 0.368 | 1.438 |  | 0.029 | -0.215 | 0.336 |
| Cefcapene pivoxil | Magnesium oxide | 0.888 | 0.449 | 1.000 |  | 0.949 | 0.749 | 1.096 |  | 1.000 | 0.382 | 2.062 |  | 0.006 | -0.453 | 0.675 |
| Cefditoren pivoxil | Acetaminophen | 0.882 | 0.410 | 1.046 |  | 1.000 | 1.000 | 1.518 |  | 1.000 | 0.427 | 2.697 |  | 0.000 | -0.632 | 1.284 |
| Cefditoren pivoxil | Loxoprofen | 0.882 | 0.410 | 1.046 |  | 0.824 | 0.611 | 1.000 |  | 1.000 | 0.389 | 1.961 |  | 0.021 | -0.462 | 0.577 |
| Cefditoren pivoxil | Tranexamic acid | 0.882 | 0.410 | 1.046 |  | 0.446 | 0.263 | 0.796 |  | 1.000 | 0.418 | 1.993 |  | 0.065 | -0.269 | 0.461 |
| Cefditoren pivoxil | Heparin sodium | 0.882 | 0.410 | 1.046 |  | 1.000 | 1.000 | 1.740 |  | 1.000 | 0.346 | 2.571 |  | 0.000 | -0.843 | 1.443 |
| Cefditoren pivoxil | L-carbocisteine | 0.882 | 0.410 | 1.046 |  | 1.000 | 0.595 | 1.000 |  | 1.000 | 0.221 | 2.244 |  | 0.000 | -0.624 | 0.708 |
| Cefditoren pivoxil | Rebamipide | 0.882 | 0.410 | 1.046 |  | 0.739 | 0.345 | 0.760 |  | 1.000 | 0.366 | 2.238 |  | 0.031 | -0.308 | 0.565 |
| Cefdinir | Methylprednisolone | 0.606 | 0.299 | 0.874 |  | 1.000 | 0.843 | 2.225 |  | 0.647 | 0.003 | 1.000 |  | -0.214 | -1.712 | -0.078 |
| Cefdinir | Aspirin | 0.606 | 0.299 | 0.874 |  | 0.825 | 0.483 | 1.000 |  | 1.000 | 0.209 | 1.000 |  | 0.069 | -0.417 | 0.253 |
| Cefdinir | Diclofenac | 0.606 | 0.299 | 0.874 |  | 1.319 | 1.189 | 2.821 |  | 1.000 | 0.230 | 2.089 |  | -0.055 | -1.559 | 0.608 |
| Cefdinir | Loxoprofen | 0.606 | 0.299 | 0.874 |  | 0.824 | 0.611 | 1.000 |  | 1.000 | 0.422 | 1.341 |  | 0.069 | -0.285 | 0.259 |
| Cefdinir | Heparin sodium | 0.606 | 0.299 | 0.874 |  | 1.000 | 1.000 | 1.740 |  | 1.000 | 0.093 | 1.000 |  | 0.000 | -1.088 | -0.041 |
| Cefdinir | Amlodipine | 0.606 | 0.299 | 0.874 |  | 0.797 | 0.467 | 0.994 |  | 1.000 | 0.564 | 2.450 |  | 0.080 | -0.079 | 0.622 |
| Cefdinir | Teprenone | 0.606 | 0.299 | 0.874 |  | 0.605 | 0.480 | 1.000 |  | 1.000 | 0.412 | 1.926 |  | 0.156 | -0.199 | 0.475 |
| Cefdinir | Rabeprazole | 0.606 | 0.299 | 0.874 |  | 1.000 | 0.538 | 1.000 |  | 0.977 | 0.203 | 1.012 |  | -0.014 | -0.426 | 0.248 |
| Cefdinir | Rebamipide | 0.606 | 0.299 | 0.874 |  | 0.739 | 0.345 | 0.760 |  | 1.000 | 0.433 | 2.179 |  | 0.103 | -0.027 | 0.541 |
| Ceftriaxone | Acetaminophen | 1.421 | 1.000 | 2.188 |  | 1.000 | 1.000 | 1.518 |  | 1.086 | 0.667 | 2.602 |  | 0.122 | -0.532 | 2.296 |
| Ceftriaxone | Heparin sodium | 1.421 | 1.000 | 2.188 |  | 1.000 | 1.000 | 1.740 |  | 1.000 | 0.659 | 2.302 |  | 0.000 | -0.571 | 2.044 |
| Cefmetazole | Acetaminophen | 1.871 | 1.484 | 3.492 |  | 1.000 | 1.000 | 1.518 |  | 1.000 | 0.658 | 2.504 |  | 0.000 | -0.744 | 4.291 |
| Cefmetazole | Loxoprofen | 1.871 | 1.484 | 3.492 |  | 0.824 | 0.611 | 1.000 |  | 1.000 | 0.197 | 1.346 |  | -0.153 | -2.203 | 0.460 |
| Cefmetazole | Methylergometrine | 1.871 | 1.484 | 3.492 |  | 0.368 | 0.080 | 0.364 |  | 1.000 | 0.284 | 1.783 |  | -0.550 | -2.292 | -0.347 |
| Cefmetazole | Heparin sodium | 1.871 | 1.484 | 3.492 |  | 1.000 | 1.000 | 1.740 |  | 1.000 | 0.239 | 1.000 |  | 0.000 | -2.378 | 0.308 |
| Cefmetazole | Metoclopramide | 1.871 | 1.484 | 3.492 |  | 1.000 | 0.765 | 1.290 |  | 0.901 | 0.179 | 1.000 |  | -0.186 | -2.364 | 0.086 |
| Cefmetazole | Teprenone | 1.871 | 1.484 | 3.492 |  | 0.605 | 0.480 | 1.000 |  | 1.000 | 0.432 | 2.163 |  | -0.344 | -1.562 | 1.625 |
| Cefmetazole | Famotidine | 1.871 | 1.484 | 3.492 |  | 1.643 | 1.332 | 2.071 |  | 1.000 | 0.331 | 1.003 |  | 0.560 | -1.977 | 0.952 |
| Cefmetazole | Magnesium oxide | 1.871 | 1.484 | 3.492 |  | 0.949 | 0.749 | 1.096 |  | 0.802 | 0.184 | 1.000 |  | -0.396 | -2.338 | 0.000 |
| Tazobactam/piperacillin | Acetaminophen | 2.162 | 1.151 | 3.484 |  | 1.000 | 1.000 | 1.518 |  | 1.123 | 0.907 | 4.536 |  | 0.266 | -0.071 | 7.006 |
| Tazobactam/piperacillin | Heparin sodium | 2.162 | 1.151 | 3.484 |  | 1.000 | 1.000 | 1.740 |  | 1.000 | 0.332 | 1.691 |  | 0.000 | -1.810 | 2.031 |
| Flomoxef | Loxoprofen | 1.000 | 0.374 | 2.086 |  | 0.824 | 0.611 | 1.000 |  | 1.000 | 0.103 | 1.978 |  | 0.000 | -0.918 | 1.091 |
| Flomoxef | Heparin sodium | 1.000 | 0.374 | 2.086 |  | 1.000 | 1.000 | 1.740 |  | 0.984 | 0.094 | 1.000 |  | -0.016 | -1.877 | 0.042 |
| Garenoxacin | L-carbocisteine | 1.000 | 0.492 | 1.554 |  | 1.000 | 0.595 | 1.000 |  | 0.996 | 0.043 | 1.000 |  | -0.004 | -1.194 | 0.056 |
| Levofloxacin | Acetaminophen | 0.711 | 0.445 | 0.846 |  | 1.000 | 1.000 | 1.518 |  | 1.000 | 0.538 | 1.468 |  | 0.000 | -0.500 | 0.257 |
| Levofloxacin | Loxoprofen | 0.711 | 0.445 | 0.846 |  | 0.824 | 0.611 | 1.000 |  | 1.000 | 0.412 | 1.515 |  | 0.051 | -0.300 | 0.335 |
| Levofloxacin | Tranexamic acid | 0.711 | 0.445 | 0.846 |  | 0.446 | 0.263 | 0.796 |  | 1.000 | 0.405 | 1.967 |  | 0.160 | -0.098 | 0.478 |
| Levofloxacin | Heparin sodium | 0.711 | 0.445 | 0.846 |  | 1.000 | 1.000 | 1.740 |  | 0.892 | 0.277 | 1.000 |  | -0.077 | -0.904 | -0.009 |
| Levofloxacin | Amlodipine | 0.711 | 0.445 | 0.846 |  | 0.797 | 0.467 | 0.994 |  | 1.000 | 0.345 | 2.225 |  | 0.059 | -0.230 | 0.612 |
| Levofloxacin | L-carbocisteine | 0.711 | 0.445 | 0.846 |  | 1.000 | 0.595 | 1.000 |  | 1.000 | 0.211 | 1.473 |  | 0.000 | -0.472 | 0.312 |
| Levofloxacin | Metoclopramide | 0.711 | 0.445 | 0.846 |  | 1.000 | 0.765 | 1.290 |  | 1.000 | 0.256 | 1.758 |  | 0.000 | -0.537 | 0.426 |
| Levofloxacin | Teprenone | 0.711 | 0.445 | 0.846 |  | 0.605 | 0.480 | 1.000 |  | 1.000 | 0.268 | 1.660 |  | 0.114 | -0.329 | 0.388 |
| Levofloxacin | Famotidine | 0.711 | 0.445 | 0.846 |  | 1.643 | 1.332 | 2.071 |  | 0.960 | 0.428 | 1.330 |  | -0.233 | -0.958 | 0.053 |
| Levofloxacin | Lansoprazole | 0.711 | 0.445 | 0.846 |  | 1.084 | 1.000 | 1.520 |  | 1.542 | 1.000 | 4.168 |  | 0.393 | -0.128 | 2.191 |

**Supplementary Table S7. Continued.**

| Drug 1 | Drug 2 | Adjusted odds ratio | | | | | | | | | | |  | Synergistic interaction | | |
| --- | --- | --- | --- | --- | --- | --- | --- | --- | --- | --- | --- | --- | --- | --- | --- | --- |
|  |  | Drug 1 | | |  | Drug 2 | | |  | Product term (Drug 1*Drug 2) | | |  |  |  |  |
|  |  | Estimate | 95%BootCI | |  | Estimate | 95%BootCI | |  | Estimate | 95%BootCI | |  | RERI | 95%BootCI | |
|  |  |  | Lower | Upper |  |  | Lower | Upper |  |  | Lower | Upper |  |  | Lower | Upper |
| Levofloxacin | Rebamipide | 0.711 | 0.445 | 0.846 |  | 0.739 | 0.345 | 0.760 |  | 1.059 | 1.000 | 4.691 |  | 0.106 | 0.088 | 1.203 |
| Levofloxacin | Magnesium oxide | 0.711 | 0.445 | 0.846 |  | 0.949 | 0.749 | 1.096 |  | 1.000 | 0.423 | 1.397 |  | 0.015 | -0.369 | 0.244 |
| Levofloxacin | Senna | 0.711 | 0.445 | 0.846 |  | 0.956 | 0.531 | 1.007 |  | 1.000 | 0.200 | 2.265 |  | 0.013 | -0.423 | 0.681 |
| Aciclovir | Heparin sodium | 1.000 | 0.485 | 2.334 |  | 1.000 | 1.000 | 1.740 |  | 1.000 | 0.202 | 1.379 |  | 0.000 | -1.840 | 0.330 |
| Azasetron | Aprepitant | 1.000 | 0.484 | 3.604 |  | 1.000 | 0.476 | 1.936 |  | 1.149 | 1.000 | 12.750 |  | 0.149 | -0.038 | 17.140 |
| Azasetron | Dexamethasone sodium phosphate | 1.000 | 0.484 | 3.604 |  | 2.176 | 3.089 | 11.277 |  | 1.000 | 0.157 | 1.000 |  | 0.000 | -7.554 | 1.612 |
| Aprepitant | Carboplatin | 1.000 | 0.476 | 1.936 |  | 1.000 | 0.080 | 1.000 |  | 1.000 | 0.949 | 12.660 |  | 0.000 | -0.302 | 4.269 |
| Aprepitant | Granisetron | 1.000 | 0.476 | 1.936 |  | 1.000 | 0.392 | 2.180 |  | 1.000 | 0.633 | 10.806 |  | 0.000 | -0.479 | 11.386 |
| Aprepitant | Cyclophosphamide | 1.000 | 0.476 | 1.936 |  | 1.000 | 0.391 | 1.000 |  | 0.745 | 0.032 | 1.000 |  | -0.255 | -1.421 | 0.000 |
| Aprepitant | Palonosetron | 1.000 | 0.476 | 1.936 |  | 0.885 | 0.167 | 1.000 |  | 1.000 | 0.426 | 3.621 |  | 0.000 | -0.576 | 2.088 |
| Aprepitant | Dexamethasone sodium phosphate | 1.000 | 0.476 | 1.936 |  | 2.176 | 3.089 | 11.277 |  | 0.938 | 0.050 | 1.000 |  | -0.135 | -9.292 | 0.000 |
| Aprepitant | Acetaminophen | 1.000 | 0.476 | 1.936 |  | 1.000 | 1.000 | 1.518 |  | 1.000 | 0.494 | 3.898 |  | 0.000 | -0.572 | 3.610 |
| Aprepitant | Heparin sodium | 1.000 | 0.476 | 1.936 |  | 1.000 | 1.000 | 1.740 |  | 1.000 | 0.178 | 1.222 |  | 0.000 | -1.320 | 0.395 |
| Aprepitant | Metoclopramide | 1.000 | 0.476 | 1.936 |  | 1.000 | 0.765 | 1.290 |  | 1.000 | 0.330 | 2.781 |  | 0.000 | -0.667 | 2.181 |
| Aprepitant | Ranitidine | 1.000 | 0.476 | 1.936 |  | 1.155 | 1.015 | 2.030 |  | 1.000 | 0.685 | 9.959 |  | 0.000 | -0.515 | 15.561 |
| Aprepitant | Magnesium oxide | 1.000 | 0.476 | 1.936 |  | 0.949 | 0.749 | 1.096 |  | 1.000 | 0.962 | 6.562 |  | 0.000 | -0.099 | 4.979 |
| Epirubicin | Cyclophosphamide | 1.000 | 0.032 | 1.000 |  | 1.000 | 0.391 | 1.000 |  | 0.727 | 0.015 | 1.000 |  | -0.273 | -0.983 | 0.239 |
| Epirubicin | Dexamethasone sodium phosphate | 1.000 | 0.032 | 1.000 |  | 2.176 | 3.089 | 11.277 |  | 1.000 | 0.599 | 1.000 |  | 0.000 | -7.933 | 0.000 |
| Epirubicin | Ranitidine | 1.000 | 0.032 | 1.000 |  | 1.155 | 1.015 | 2.030 |  | 1.000 | 1.000 | 22.250 |  | 0.000 | -0.798 | 28.048 |
| Carboplatin | Paclitaxel | 1.000 | 0.080 | 1.000 |  | 1.000 | 0.970 | 10.773 |  | 1.000 | 0.299 | 3.053 |  | 0.000 | -5.832 | 0.592 |
| Carboplatin | Dexamethasone sodium phosphate | 1.000 | 0.080 | 1.000 |  | 2.176 | 3.089 | 11.277 |  | 0.899 | 0.211 | 1.000 |  | -0.219 | -7.909 | 0.000 |
| Carboplatin | Ranitidine | 1.000 | 0.080 | 1.000 |  | 1.155 | 1.015 | 2.030 |  | 0.819 | 0.143 | 1.041 |  | -0.209 | -1.274 | 0.000 |
| Carboplatin | Magnesium oxide | 1.000 | 0.080 | 1.000 |  | 0.949 | 0.749 | 1.096 |  | 1.000 | 0.313 | 3.596 |  | 0.000 | -0.522 | 0.987 |
| Granisetron | Cyclophosphamide | 1.000 | 0.392 | 2.180 |  | 1.000 | 0.391 | 1.000 |  | 1.000 | 0.209 | 2.326 |  | 0.000 | -1.206 | 1.173 |
| Granisetron | Dexamethasone sodium phosphate | 1.000 | 0.392 | 2.180 |  | 2.176 | 3.089 | 11.277 |  | 0.832 | 0.072 | 1.000 |  | -0.366 | -9.516 | -0.419 |
| Granisetron | Heparin sodium | 1.000 | 0.392 | 2.180 |  | 1.000 | 1.000 | 1.740 |  | 1.194 | 1.000 | 7.386 |  | 0.194 | -0.143 | 9.224 |
| Granisetron | Metoclopramide | 1.000 | 0.392 | 2.180 |  | 1.000 | 0.765 | 1.290 |  | 1.000 | 0.717 | 6.145 |  | 0.000 | -0.306 | 5.587 |
| Granisetron | Magnesium oxide | 1.000 | 0.392 | 2.180 |  | 0.949 | 0.749 | 1.096 |  | 1.000 | 0.202 | 1.938 |  | 0.000 | -1.207 | 0.854 |
| Goserelin | Bicalutamide | 1.000 | 0.153 | 1.424 |  | 1.000 | 0.593 | 2.110 |  | 1.062 | 0.817 | 9.331 |  | 0.062 | -0.444 | 2.026 |
| Cyclophosphamide | Doxorubicin | 1.000 | 0.391 | 1.000 |  | 1.000 | 0.115 | 1.146 |  | 1.000 | 0.440 | 1.000 |  | 0.000 | -0.514 | 0.218 |
| Cyclophosphamide | Palonosetron | 1.000 | 0.391 | 1.000 |  | 0.885 | 0.167 | 1.000 |  | 0.211 | 0.027 | 1.000 |  | -0.699 | -0.943 | 0.207 |
| Cyclophosphamide | Dexamethasone sodium phosphate | 1.000 | 0.391 | 1.000 |  | 2.176 | 3.089 | 11.277 |  | 0.627 | 0.172 | 1.613 |  | -0.811 | -5.763 | 4.737 |
| Cyclophosphamide | Prednisolone | 1.000 | 0.391 | 1.000 |  | 1.000 | 0.950 | 1.606 |  | 1.000 | 0.117 | 1.000 |  | 0.000 | -1.058 | 0.000 |
| Cyclophosphamide | Acetaminophen | 1.000 | 0.391 | 1.000 |  | 1.000 | 1.000 | 1.518 |  | 0.777 | 0.021 | 1.000 |  | -0.223 | -1.333 | 0.000 |
| Cyclophosphamide | Ranitidine | 1.000 | 0.391 | 1.000 |  | 1.155 | 1.015 | 2.030 |  | 0.995 | 0.070 | 1.956 |  | -0.006 | -1.466 | 1.265 |
| Cisplatin | Dexamethasone sodium phosphate | 1.000 | 0.102 | 1.000 |  | 2.176 | 3.089 | 11.277 |  | 1.000 | 0.308 | 4.206 |  | 0.000 | -6.743 | 1.297 |
| Doxorubicin | Vincristine | 1.000 | 0.115 | 1.146 |  | 1.110 | 1.000 | 40.349 |  | 1.000 | 1.000 | 2.243 |  | 0.000 | -23.393 | 1.219 |
| Doxorubicin | Prednisolone | 1.000 | 0.115 | 1.146 |  | 1.000 | 0.950 | 1.606 |  | 1.000 | 0.080 | 1.000 |  | 0.000 | -1.008 | 0.012 |
| Paclitaxel | Dexamethasone sodium phosphate | 1.000 | 0.970 | 10.773 |  | 2.176 | 3.089 | 11.277 |  | 1.000 | 0.086 | 1.003 |  | 0.000 | -9.068 | 9.461 |
| Paclitaxel | Ranitidine | 1.000 | 0.970 | 10.773 |  | 1.155 | 1.015 | 2.030 |  | 1.000 | 0.264 | 2.329 |  | 0.000 | -1.801 | 6.581 |
| Palonosetron | Dexamethasone sodium phosphate | 0.885 | 0.167 | 1.000 |  | 2.176 | 3.089 | 11.277 |  | 1.000 | 0.209 | 1.000 |  | -0.135 | -7.521 | 0.000 |
| Palonosetron | Acetaminophen | 0.885 | 0.167 | 1.000 |  | 1.000 | 1.000 | 1.518 |  | 0.836 | 0.120 | 1.744 |  | -0.145 | -1.043 | 0.522 |
| Palonosetron | Chlorpheniramine | 0.885 | 0.167 | 1.000 |  | 0.920 | 0.571 | 1.688 |  | 1.000 | 0.256 | 6.607 |  | 0.009 | -0.671 | 3.667 |
| Palonosetron | Ranitidine | 0.885 | 0.167 | 1.000 |  | 1.155 | 1.015 | 2.030 |  | 1.000 | 0.375 | 6.330 |  | -0.018 | -0.812 | 5.035 |
| Palonosetron | Magnesium oxide | 0.885 | 0.167 | 1.000 |  | 0.949 | 0.749 | 1.096 |  | 1.000 | 0.314 | 3.735 |  | 0.006 | -0.605 | 1.731 |
| Bicalutamide | Leuprorelin | 1.000 | 0.593 | 2.110 |  | 1.000 | 0.629 | 2.283 |  | 0.599 | 0.036 | 0.673 |  | -0.401 | -2.501 | -0.226 |

**Supplementary Table S7. Continued.**

| Drug 1 | Drug 2 | Adjusted odds ratio | | | | | | | | | | |  | Synergistic interaction | | |
| --- | --- | --- | --- | --- | --- | --- | --- | --- | --- | --- | --- | --- | --- | --- | --- | --- |
|  |  | Drug 1 | | |  | Drug 2 | | |  | Product term (Drug 1*Drug 2) | | |  |  |  |  |
|  |  | Estimate | 95%BootCI | |  | Estimate | 95%BootCI | |  | Estimate | 95%BootCI | |  | RERI | 95%BootCI | |
|  |  |  | Lower | Upper |  |  | Lower | Upper |  |  | Lower | Upper |  |  | Lower | Upper |
| Vincristine | Prednisolone | 1.110 | 1.000 | 40.349 |  | 1.000 | 0.950 | 1.606 |  | 1.000 | 1.000 | 2.254 |  | 0.000 | -0.072 | 10.899 |
| Fluorouracil | Dexamethasone sodium phosphate | 1.000 | 0.726 | 6.641 |  | 2.176 | 3.089 | 11.277 |  | 1.000 | 0.078 | 1.000 |  | 0.000 | -9.190 | 2.483 |
| Methotrexate | Prednisolone | 1.000 | 0.478 | 1.735 |  | 1.000 | 0.950 | 1.606 |  | 1.000 | 0.083 | 1.000 |  | 0.000 | -1.607 | 0.000 |
| Ramosetron | Dexamethasone sodium phosphate | 1.000 | 0.488 | 4.081 |  | 2.176 | 3.089 | 11.277 |  | 0.545 | 0.043 | 1.000 |  | -0.989 | -10.010 | -0.663 |
| Dexamethasone sodium phosphate | Acetaminophen | 2.176 | 3.089 | 11.277 |  | 1.000 | 1.000 | 1.518 |  | 1.000 | 0.507 | 2.494 |  | 0.000 | -2.987 | 14.646 |
| Dexamethasone sodium phosphate | Loxoprofen | 2.176 | 3.089 | 11.277 |  | 0.824 | 0.611 | 1.000 |  | 1.000 | 0.368 | 1.766 |  | -0.207 | -5.425 | 2.962 |
| Dexamethasone sodium phosphate | Chlorpheniramine | 2.176 | 3.089 | 11.277 |  | 0.920 | 0.571 | 1.688 |  | 0.873 | 0.205 | 1.152 |  | -0.347 | -6.607 | 1.994 |
| Dexamethasone sodium phosphate | Adenine | 2.176 | 3.089 | 11.277 |  | 1.000 | 0.216 | 15.902 |  | 0.701 | 0.020 | 1.000 |  | -0.651 | -16.733 | 15.049 |
| Dexamethasone sodium phosphate | Heparin sodium | 2.176 | 3.089 | 11.277 |  | 1.000 | 1.000 | 1.740 |  | 1.009 | 0.741 | 2.498 |  | 0.020 | -0.607 | 14.796 |
| Dexamethasone sodium phosphate | Amlodipine | 2.176 | 3.089 | 11.277 |  | 0.797 | 0.467 | 0.994 |  | 0.890 | 0.147 | 1.000 |  | -0.430 | -8.439 | -0.652 |
| Dexamethasone sodium phosphate | Furosemide | 2.176 | 3.089 | 11.277 |  | 1.204 | 1.079 | 2.334 |  | 1.000 | 0.503 | 2.507 |  | 0.240 | -1.865 | 19.362 |
| Dexamethasone sodium phosphate | Metoclopramide | 2.176 | 3.089 | 11.277 |  | 1.000 | 0.765 | 1.290 |  | 0.784 | 0.314 | 1.308 |  | -0.469 | -5.135 | 2.337 |
| Dexamethasone sodium phosphate | Famotidine | 2.176 | 3.089 | 11.277 |  | 1.643 | 1.332 | 2.071 |  | 1.000 | 0.350 | 1.345 |  | 0.756 | -4.283 | 6.635 |
| Dexamethasone sodium phosphate | Ranitidine | 2.176 | 3.089 | 11.277 |  | 1.155 | 1.015 | 2.030 |  | 1.000 | 0.515 | 2.643 |  | 0.183 | -2.232 | 17.891 |
| Dexamethasone sodium phosphate | Rabeprazole | 2.176 | 3.089 | 11.277 |  | 1.000 | 0.538 | 1.000 |  | 1.000 | 0.251 | 2.155 |  | 0.000 | -6.357 | 4.623 |
| Dexamethasone sodium phosphate | Lansoprazole | 2.176 | 3.089 | 11.277 |  | 1.084 | 1.000 | 1.520 |  | 1.000 | 0.354 | 1.743 |  | 0.098 | -4.629 | 5.842 |
| Dexamethasone sodium phosphate | Rebamipide | 2.176 | 3.089 | 11.277 |  | 0.739 | 0.345 | 0.760 |  | 1.457 | 0.835 | 4.681 |  | 0.429 | -4.633 | 8.404 |
| Dexamethasone sodium phosphate | Magnesium oxide | 2.176 | 3.089 | 11.277 |  | 0.949 | 0.749 | 1.096 |  | 0.535 | 0.115 | 0.863 |  | -1.020 | -8.383 | -0.794 |
| Hydrocortisone | Acetaminophen | 1.089 | 1.000 | 3.856 |  | 1.000 | 1.000 | 1.518 |  | 1.000 | 0.206 | 1.383 |  | 0.000 | -2.357 | 1.009 |
| Hydrocortisone | Loxoprofen | 1.089 | 1.000 | 3.856 |  | 0.824 | 0.611 | 1.000 |  | 1.000 | 0.277 | 3.206 |  | -0.016 | -1.753 | 3.533 |
| Hydrocortisone | Tranexamic acid | 1.089 | 1.000 | 3.856 |  | 0.446 | 0.263 | 0.796 |  | 1.000 | 0.036 | 1.000 |  | -0.049 | -2.755 | -0.070 |
| Hydrocortisone | Heparin sodium | 1.089 | 1.000 | 3.856 |  | 1.000 | 1.000 | 1.740 |  | 0.630 | 0.153 | 1.000 |  | -0.402 | -2.955 | 0.241 |
| Hydrocortisone | L-carbocisteine | 1.089 | 1.000 | 3.856 |  | 1.000 | 0.595 | 1.000 |  | 1.000 | 0.332 | 4.019 |  | 0.000 | -1.742 | 4.524 |
| Hydrocortisone | Metoclopramide | 1.089 | 1.000 | 3.856 |  | 1.000 | 0.765 | 1.290 |  | 0.920 | 0.094 | 1.000 |  | -0.087 | -2.863 | 0.197 |
| Hydrocortisone | Rebamipide | 1.089 | 1.000 | 3.856 |  | 0.739 | 0.345 | 0.760 |  | 0.618 | 0.011 | 1.000 |  | -0.331 | -3.072 | -0.208 |
| Prednisolone | Aspirin | 1.000 | 0.950 | 1.606 |  | 0.825 | 0.483 | 1.000 |  | 1.000 | 0.235 | 2.064 |  | 0.000 | -0.807 | 0.793 |
| Prednisolone | Acetaminophen | 1.000 | 0.950 | 1.606 |  | 1.000 | 1.000 | 1.518 |  | 1.000 | 0.647 | 2.219 |  | 0.000 | -0.450 | 1.602 |
| Prednisolone | Celecoxib | 1.000 | 0.950 | 1.606 |  | 0.498 | 0.232 | 0.796 |  | 0.851 | 0.081 | 1.000 |  | -0.074 | -0.858 | 0.000 |
| Prednisolone | Loxoprofen | 1.000 | 0.950 | 1.606 |  | 0.824 | 0.611 | 1.000 |  | 0.853 | 0.184 | 1.000 |  | -0.121 | -1.008 | 0.000 |
| Prednisolone | Salazosulfapyridine | 1.000 | 0.950 | 1.606 |  | 1.000 | 0.209 | 1.000 |  | 1.000 | 0.143 | 1.136 |  | 0.000 | -0.990 | 0.100 |
| Prednisolone | Chlorpheniramine | 1.000 | 0.950 | 1.606 |  | 0.920 | 0.571 | 1.688 |  | 1.000 | 0.088 | 2.011 |  | 0.000 | -1.275 | 1.186 |
| Prednisolone | Alfacalcidol | 1.000 | 0.950 | 1.606 |  | 0.503 | 0.146 | 0.632 |  | 1.000 | 0.505 | 2.406 |  | 0.000 | -0.475 | 0.374 |
| Prednisolone | Alendronate | 1.000 | 0.950 | 1.606 |  | 1.000 | 0.549 | 1.457 |  | 1.000 | 0.350 | 1.746 |  | 0.000 | -0.855 | 0.710 |
| Prednisolone | Heparin sodium | 1.000 | 0.950 | 1.606 |  | 1.000 | 1.000 | 1.740 |  | 2.017 | 1.000 | 4.359 |  | 1.017 | 0.121 | 5.307 |
| Prednisolone | Amlodipine | 1.000 | 0.950 | 1.606 |  | 0.797 | 0.467 | 0.994 |  | 1.000 | 0.299 | 1.819 |  | 0.000 | -0.675 | 0.559 |
| Prednisolone | Furosemide | 1.000 | 0.950 | 1.606 |  | 1.204 | 1.079 | 2.334 |  | 1.045 | 0.742 | 3.007 |  | 0.054 | -0.412 | 3.623 |
| Prednisolone | L-carbocisteine | 1.000 | 0.950 | 1.606 |  | 1.000 | 0.595 | 1.000 |  | 0.893 | 0.128 | 1.032 |  | -0.107 | -1.122 | 0.003 |
| Prednisolone | Esomeprazole | 1.000 | 0.950 | 1.606 |  | 0.671 | 0.373 | 0.954 |  | 1.000 | 0.761 | 2.757 |  | 0.000 | -0.297 | 0.938 |
| Prednisolone | Omeprazole | 1.000 | 0.950 | 1.606 |  | 1.045 | 0.928 | 1.644 |  | 1.397 | 0.856 | 3.220 |  | 0.415 | -0.146 | 2.665 |
| Prednisolone | Teprenone | 1.000 | 0.950 | 1.606 |  | 0.605 | 0.480 | 1.000 |  | 1.000 | 0.292 | 1.222 |  | 0.000 | -0.761 | 0.136 |
| Prednisolone | Famotidine | 1.000 | 0.950 | 1.606 |  | 1.643 | 1.332 | 2.071 |  | 1.000 | 0.428 | 1.337 |  | 0.000 | -1.070 | 0.639 |
| Prednisolone | Rabeprazole | 1.000 | 0.950 | 1.606 |  | 1.000 | 0.538 | 1.000 |  | 1.000 | 0.775 | 2.220 |  | 0.000 | -0.266 | 1.016 |
| Prednisolone | Lansoprazole | 1.000 | 0.950 | 1.606 |  | 1.084 | 1.000 | 1.520 |  | 1.000 | 0.413 | 1.295 |  | 0.000 | -0.877 | 0.390 |
| Prednisolone | Rebamipide | 1.000 | 0.950 | 1.606 |  | 0.739 | 0.345 | 0.760 |  | 1.000 | 0.770 | 2.928 |  | 0.000 | -0.269 | 1.042 |
| Prednisolone | Magnesium oxide | 1.000 | 0.950 | 1.606 |  | 0.949 | 0.749 | 1.096 |  | 1.000 | 0.650 | 2.671 |  | 0.000 | -0.380 | 1.901 |
| Prednisolone | Sennoside | 1.000 | 0.950 | 1.606 |  | 1.000 | 0.852 | 1.372 |  | 1.210 | 1.000 | 6.501 |  | 0.210 | 0.000 | 6.689 |

**Supplementary Table S7. Continued.**

| Drug 1 | Drug 2 | Adjusted odds ratio | | | | | | | | | | |  | Synergistic interaction | | |
| --- | --- | --- | --- | --- | --- | --- | --- | --- | --- | --- | --- | --- | --- | --- | --- | --- |
|  |  | Drug 1 | | |  | Drug 2 | | |  | Product term (Drug 1*Drug 2) | | |  |  |  |  |
|  |  | Estimate | 95%BootCI | |  | Estimate | 95%BootCI | |  | Estimate | 95%BootCI | |  | RERI | 95%BootCI | |
|  |  |  | Lower | Upper |  |  | Lower | Upper |  |  | Lower | Upper |  |  | Lower | Upper |
| Betamethasone | Chlorpheniramine | 1.000 | 1.000 | 7.722 |  | 0.920 | 0.571 | 1.688 |  | 0.959 | 0.042 | 1.000 |  | -0.038 | -7.193 | 0.000 |
| Methylprednisolone | Loxoprofen | 1.000 | 0.843 | 2.225 |  | 0.824 | 0.611 | 1.000 |  | 1.000 | 1.000 | 19.986 |  | 0.000 | -0.164 | 19.274 |
| Methylprednisolone | Heparin sodium | 1.000 | 0.843 | 2.225 |  | 1.000 | 1.000 | 1.740 |  | 1.011 | 0.563 | 2.557 |  | 0.011 | -0.702 | 2.372 |
| Methylprednisolone | Rabeprazole | 1.000 | 0.843 | 2.225 |  | 1.000 | 0.538 | 1.000 |  | 1.000 | 0.394 | 3.673 |  | 0.000 | -0.767 | 2.311 |
| Methylprednisolone | Rebamipide | 1.000 | 0.843 | 2.225 |  | 0.739 | 0.345 | 0.760 |  | 1.000 | 0.199 | 1.567 |  | 0.000 | -0.952 | 0.290 |
| Aspirin | Acetaminophen | 0.825 | 0.483 | 1.000 |  | 1.000 | 1.000 | 1.518 |  | 1.000 | 0.664 | 2.551 |  | 0.000 | -0.360 | 1.192 |
| Aspirin | Loxoprofen | 0.825 | 0.483 | 1.000 |  | 0.824 | 0.611 | 1.000 |  | 1.000 | 0.949 | 3.013 |  | 0.031 | -0.005 | 1.224 |
| Aspirin | Imidapril | 0.825 | 0.483 | 1.000 |  | 0.747 | 0.245 | 1.000 |  | 1.000 | 0.465 | 2.667 |  | 0.044 | -0.287 | 0.614 |
| Aspirin | Insulin (human) | 0.825 | 0.483 | 1.000 |  | 2.511 | 1.398 | 3.931 |  | 0.979 | 0.550 | 2.792 |  | -0.308 | -1.444 | 2.924 |
| Aspirin | Glimepiride | 0.825 | 0.483 | 1.000 |  | 0.821 | 0.290 | 1.000 |  | 0.915 | 0.133 | 1.000 |  | -0.026 | -0.526 | 0.249 |
| Aspirin | Sitagliptin | 0.825 | 0.483 | 1.000 |  | 0.590 | 0.229 | 1.000 |  | 1.000 | 0.319 | 3.420 |  | 0.072 | -0.250 | 0.992 |
| Aspirin | Voglibose | 0.825 | 0.483 | 1.000 |  | 1.000 | 0.538 | 1.446 |  | 0.745 | 0.117 | 1.000 |  | -0.211 | -0.859 | 0.127 |
| Aspirin | Metformin | 0.825 | 0.483 | 1.000 |  | 0.500 | 0.228 | 0.948 |  | 1.000 | 0.272 | 2.568 |  | 0.087 | -0.246 | 0.631 |
| Aspirin | Losartan | 0.825 | 0.483 | 1.000 |  | 0.776 | 0.341 | 1.000 |  | 1.000 | 0.244 | 1.528 |  | 0.039 | -0.488 | 0.370 |
| Aspirin | Atorvastatin | 0.825 | 0.483 | 1.000 |  | 0.729 | 0.259 | 0.789 |  | 0.926 | 0.323 | 1.000 |  | 0.003 | -0.291 | 0.289 |
| Aspirin | Pitavastatin | 0.825 | 0.483 | 1.000 |  | 0.800 | 0.413 | 1.000 |  | 1.000 | 0.454 | 1.882 |  | 0.035 | -0.347 | 0.471 |
| Aspirin | Pravastatin | 0.825 | 0.483 | 1.000 |  | 0.739 | 0.208 | 0.768 |  | 1.000 | 0.412 | 2.173 |  | 0.046 | -0.190 | 0.476 |
| Aspirin | Rosuvastatin | 0.825 | 0.483 | 1.000 |  | 0.685 | 0.203 | 0.698 |  | 1.000 | 0.490 | 1.350 |  | 0.055 | -0.119 | 0.347 |
| Aspirin | Allopurinol | 0.825 | 0.483 | 1.000 |  | 1.000 | 0.686 | 1.330 |  | 1.000 | 0.420 | 1.646 |  | 0.000 | -0.486 | 0.448 |
| Aspirin | Febuxostat | 0.825 | 0.483 | 1.000 |  | 0.626 | 0.226 | 0.986 |  | 0.685 | 0.066 | 1.000 |  | -0.097 | -0.613 | 0.231 |
| Aspirin | Alfacalcidol | 0.825 | 0.483 | 1.000 |  | 0.503 | 0.146 | 0.632 |  | 1.000 | 0.283 | 1.000 |  | 0.087 | -0.201 | 0.365 |
| Aspirin | Clopidogrel | 0.825 | 0.483 | 1.000 |  | 0.808 | 0.309 | 1.000 |  | 1.000 | 1.000 | 3.017 |  | 0.034 | 0.000 | 0.842 |
| Aspirin | Cilostazol | 0.825 | 0.483 | 1.000 |  | 0.815 | 0.379 | 1.000 |  | 1.232 | 1.143 | 8.138 |  | 0.188 | 0.135 | 3.010 |
| Aspirin | Ticlopidine | 0.825 | 0.483 | 1.000 |  | 1.000 | 0.742 | 2.285 |  | 1.214 | 0.870 | 2.972 |  | 0.177 | -0.444 | 1.392 |
| Aspirin | Prasugrel | 0.825 | 0.483 | 1.000 |  | 1.000 | 0.398 | 1.440 |  | 1.000 | 0.418 | 1.349 |  | 0.000 | -0.462 | 0.281 |
| Aspirin | Heparin sodium | 0.825 | 0.483 | 1.000 |  | 1.000 | 1.000 | 1.740 |  | 1.000 | 0.730 | 1.763 |  | 0.000 | -0.398 | 0.623 |
| Aspirin | Warfarin | 0.825 | 0.483 | 1.000 |  | 0.907 | 0.652 | 1.285 |  | 1.000 | 0.475 | 1.506 |  | 0.016 | -0.424 | 0.325 |
| Aspirin | Amlodipine | 0.825 | 0.483 | 1.000 |  | 0.797 | 0.467 | 0.994 |  | 1.000 | 0.643 | 1.594 |  | 0.035 | -0.184 | 0.379 |
| Aspirin | Enalapril | 0.825 | 0.483 | 1.000 |  | 0.812 | 0.317 | 1.000 |  | 0.937 | 0.202 | 1.149 |  | -0.009 | -0.589 | 0.255 |
| Aspirin | Eplerenone | 0.825 | 0.483 | 1.000 |  | 1.000 | 0.400 | 1.338 |  | 1.000 | 0.177 | 1.452 |  | 0.000 | -0.677 | 0.335 |
| Aspirin | Olmesartan | 0.825 | 0.483 | 1.000 |  | 1.000 | 0.339 | 1.000 |  | 1.000 | 0.383 | 1.254 |  | 0.000 | -0.364 | 0.295 |
| Aspirin | Carvedilol | 0.825 | 0.483 | 1.000 |  | 1.000 | 0.765 | 1.536 |  | 1.000 | 0.805 | 1.741 |  | 0.000 | -0.215 | 0.519 |
| Aspirin | Candesartan | 0.825 | 0.483 | 1.000 |  | 0.854 | 0.400 | 1.000 |  | 0.764 | 0.219 | 1.000 |  | -0.140 | -0.568 | 0.173 |
| Aspirin | Diltiazem | 0.825 | 0.483 | 1.000 |  | 1.000 | 0.384 | 1.137 |  | 1.000 | 0.208 | 1.602 |  | 0.000 | -0.604 | 0.378 |
| Aspirin | Spironolactone | 0.825 | 0.483 | 1.000 |  | 1.000 | 0.452 | 1.310 |  | 1.000 | 0.852 | 2.595 |  | 0.000 | -0.119 | 1.039 |
| Aspirin | Telmisartan | 0.825 | 0.483 | 1.000 |  | 0.603 | 0.225 | 0.872 |  | 1.000 | 0.434 | 2.240 |  | 0.069 | -0.214 | 0.523 |
| Aspirin | Doxazosin | 0.825 | 0.483 | 1.000 |  | 1.000 | 0.505 | 1.310 |  | 1.000 | 0.200 | 2.497 |  | 0.000 | -0.599 | 1.023 |
| Aspirin | Nitroglycerin | 0.825 | 0.483 | 1.000 |  | 0.975 | 0.335 | 1.142 |  | 0.952 | 0.428 | 2.297 |  | -0.035 | -0.412 | 0.612 |
| Aspirin | Nifedipine | 0.825 | 0.483 | 1.000 |  | 1.000 | 0.710 | 1.340 |  | 1.000 | 0.473 | 1.484 |  | 0.000 | -0.459 | 0.344 |
| Aspirin | Bisoprolol | 0.825 | 0.483 | 1.000 |  | 1.000 | 0.621 | 1.236 |  | 1.000 | 0.437 | 1.371 |  | 0.000 | -0.443 | 0.284 |
| Aspirin | Furosemide | 0.825 | 0.483 | 1.000 |  | 1.204 | 1.079 | 2.334 |  | 0.965 | 0.498 | 1.144 |  | -0.071 | -0.949 | 0.039 |
| Aspirin | Benidipine | 0.825 | 0.483 | 1.000 |  | 0.714 | 0.353 | 1.000 |  | 0.661 | 0.165 | 1.000 |  | -0.150 | -0.544 | 0.210 |
| Aspirin | Perindopril | 0.825 | 0.483 | 1.000 |  | 0.914 | 0.299 | 1.021 |  | 1.000 | 0.530 | 3.058 |  | 0.015 | -0.290 | 0.786 |
| Aspirin | Nicorandil | 0.825 | 0.483 | 1.000 |  | 1.000 | 0.861 | 2.193 |  | 0.853 | 0.442 | 1.046 |  | -0.121 | -0.919 | 0.059 |
| Aspirin | Isosorbide mononitrate | 0.825 | 0.483 | 1.000 |  | 1.000 | 0.491 | 1.492 |  | 1.000 | 0.743 | 2.829 |  | 0.000 | -0.265 | 1.042 |
| Aspirin | Azosemide | 0.825 | 0.483 | 1.000 |  | 1.000 | 0.436 | 1.093 |  | 1.000 | 0.565 | 3.212 |  | 0.000 | -0.276 | 1.095 |

**Supplementary Table S7. Continued.**

| Drug 1 | Drug 2 | Adjusted odds ratio | | | | | | | | | | |  | Synergistic interaction | | |
| --- | --- | --- | --- | --- | --- | --- | --- | --- | --- | --- | --- | --- | --- | --- | --- | --- |
|  |  | Drug 1 | | |  | Drug 2 | | |  | Product term (Drug 1*Drug 2) | | |  |  |  |  |
|  |  | Estimate | 95%BootCI | |  | Estimate | 95%BootCI | |  | Estimate | 95%BootCI | |  | RERI | 95%BootCI | |
|  |  |  | Lower | Upper |  |  | Lower | Upper |  |  | Lower | Upper |  |  | Lower | Upper |
| Aspirin | Ambroxol | 0.825 | 0.483 | 1.000 |  | 1.000 | 0.702 | 1.258 |  | 1.024 | 1.000 | 4.521 |  | 0.020 | -0.036 | 2.527 |
| Aspirin | L-carbocisteine | 0.825 | 0.483 | 1.000 |  | 1.000 | 0.595 | 1.000 |  | 1.000 | 0.568 | 2.209 |  | 0.000 | -0.271 | 0.768 |
| Aspirin | Metoclopramide | 0.825 | 0.483 | 1.000 |  | 1.000 | 0.765 | 1.290 |  | 1.000 | 0.646 | 3.440 |  | 0.000 | -0.268 | 1.752 |
| Aspirin | Mosapride | 0.825 | 0.483 | 1.000 |  | 1.000 | 0.620 | 1.251 |  | 1.068 | 0.608 | 2.741 |  | 0.056 | -0.302 | 1.136 |
| Aspirin | Esomeprazole | 0.825 | 0.483 | 1.000 |  | 0.671 | 0.373 | 0.954 |  | 1.000 | 0.437 | 1.175 |  | 0.057 | -0.292 | 0.272 |
| Aspirin | Omeprazole | 0.825 | 0.483 | 1.000 |  | 1.045 | 0.928 | 1.644 |  | 1.000 | 0.444 | 1.155 |  | -0.008 | -0.701 | 0.096 |
| Aspirin | Teprenone | 0.825 | 0.483 | 1.000 |  | 0.605 | 0.480 | 1.000 |  | 1.000 | 0.408 | 2.244 |  | 0.069 | -0.313 | 0.658 |
| Aspirin | Famotidine | 0.825 | 0.483 | 1.000 |  | 1.643 | 1.332 | 2.071 |  | 1.000 | 0.517 | 1.247 |  | -0.112 | -0.871 | 0.093 |
| Aspirin | Vonoprazan | 0.825 | 0.483 | 1.000 |  | 1.000 | 0.392 | 1.000 |  | 1.000 | 0.564 | 2.693 |  | 0.000 | -0.254 | 0.926 |
| Aspirin | Ranitidine | 0.825 | 0.483 | 1.000 |  | 1.155 | 1.015 | 2.030 |  | 0.872 | 0.099 | 1.000 |  | -0.149 | -1.413 | -0.003 |
| Aspirin | Rabeprazole | 0.825 | 0.483 | 1.000 |  | 1.000 | 0.538 | 1.000 |  | 1.000 | 0.896 | 2.189 |  | 0.000 | -0.029 | 0.626 |
| Aspirin | Lansoprazole | 0.825 | 0.483 | 1.000 |  | 1.084 | 1.000 | 1.520 |  | 1.000 | 0.746 | 1.579 |  | -0.015 | -0.334 | 0.422 |
| Aspirin | Rebamipide | 0.825 | 0.483 | 1.000 |  | 0.739 | 0.345 | 0.760 |  | 1.000 | 0.425 | 2.045 |  | 0.045 | -0.180 | 0.504 |
| Aspirin | Magnesium oxide | 0.825 | 0.483 | 1.000 |  | 0.949 | 0.749 | 1.096 |  | 1.000 | 0.581 | 1.925 |  | 0.009 | -0.333 | 0.590 |
| Aspirin | Senna | 0.825 | 0.483 | 1.000 |  | 0.956 | 0.531 | 1.007 |  | 0.522 | 0.236 | 1.223 |  | -0.370 | -0.567 | 0.193 |
| Aspirin | Sennoside | 0.825 | 0.483 | 1.000 |  | 1.000 | 0.852 | 1.372 |  | 0.623 | 0.151 | 1.000 |  | -0.311 | -0.814 | 0.000 |
| Aspirin | Etizolam | 0.825 | 0.483 | 1.000 |  | 0.805 | 0.270 | 0.865 |  | 1.000 | 0.340 | 1.944 |  | 0.034 | -0.264 | 0.460 |
| Aspirin | Diazepam | 0.825 | 0.483 | 1.000 |  | 1.000 | 0.539 | 1.445 |  | 1.594 | 1.000 | 5.149 |  | 0.490 | -0.027 | 2.658 |
| Aspirin | Zolpidem | 0.825 | 0.483 | 1.000 |  | 1.000 | 0.767 | 1.233 |  | 1.000 | 0.348 | 1.476 |  | 0.000 | -0.518 | 0.333 |
| Aspirin | Brotizolam | 0.825 | 0.483 | 1.000 |  | 1.000 | 0.621 | 1.195 |  | 1.000 | 0.207 | 1.000 |  | 0.000 | -0.654 | 0.092 |
| Aspirin | Edaravone | 0.825 | 0.483 | 1.000 |  | 1.000 | 0.616 | 2.595 |  | 0.612 | 0.067 | 1.000 |  | -0.320 | -1.765 | 0.046 |
| Aspirin | Polystyrene sulfonate | 0.825 | 0.483 | 1.000 |  | 1.000 | 0.612 | 1.479 |  | 1.000 | 0.230 | 1.606 |  | 0.000 | -0.706 | 0.414 |
| Aspirin | Tamsulosin | 0.825 | 0.483 | 1.000 |  | 0.711 | 0.383 | 0.923 |  | 0.510 | 0.068 | 1.000 |  | -0.237 | -0.561 | 0.200 |
| Acetaminophen | Salicylamide | 1.000 | 1.000 | 1.518 |  | 0.910 | 0.165 | 1.000 |  | 0.583 | 0.141 | 1.000 |  | -0.380 | -1.125 | 0.000 |
| Acetaminophen | Diclofenac | 1.000 | 1.000 | 1.518 |  | 1.319 | 1.189 | 2.821 |  | 1.078 | 0.804 | 2.603 |  | 0.088 | -0.318 | 3.632 |
| Acetaminophen | Celecoxib | 1.000 | 1.000 | 1.518 |  | 0.498 | 0.232 | 0.796 |  | 1.000 | 0.627 | 2.473 |  | 0.000 | -0.386 | 0.565 |
| Acetaminophen | Tramadol | 1.000 | 1.000 | 1.518 |  | 1.000 | 0.331 | 1.287 |  | 0.705 | 0.171 | 1.000 |  | -0.295 | -1.226 | -0.016 |
| Acetaminophen | Flurbiprofen | 1.000 | 1.000 | 1.518 |  | 1.000 | 0.509 | 1.123 |  | 1.000 | 0.706 | 2.898 |  | 0.000 | -0.328 | 1.877 |
| Acetaminophen | Pregabalin | 1.000 | 1.000 | 1.518 |  | 1.000 | 0.183 | 0.970 |  | 1.000 | 0.772 | 4.069 |  | 0.000 | -0.302 | 0.959 |
| Acetaminophen | Loxoprofen | 1.000 | 1.000 | 1.518 |  | 0.824 | 0.611 | 1.000 |  | 1.000 | 0.882 | 2.097 |  | 0.000 | -0.168 | 1.076 |
| Acetaminophen | Chlorpheniramine | 1.000 | 1.000 | 1.518 |  | 0.920 | 0.571 | 1.688 |  | 1.000 | 0.412 | 2.276 |  | 0.000 | -0.738 | 1.602 |
| Acetaminophen | Olopatadine | 1.000 | 1.000 | 1.518 |  | 1.000 | 0.575 | 1.169 |  | 1.000 | 0.773 | 3.809 |  | 0.000 | -0.266 | 2.909 |
| Acetaminophen | Hydroxyzine | 1.000 | 1.000 | 1.518 |  | 1.000 | 0.545 | 1.272 |  | 1.000 | 0.553 | 1.305 |  | 0.000 | -0.531 | 0.331 |
| Acetaminophen | Fexofenadine | 1.000 | 1.000 | 1.518 |  | 1.000 | 0.598 | 1.549 |  | 1.000 | 0.108 | 1.000 |  | 0.000 | -1.303 | 0.000 |
| Acetaminophen | Montelukast | 1.000 | 1.000 | 1.518 |  | 0.723 | 0.161 | 1.000 |  | 1.000 | 0.303 | 4.908 |  | 0.000 | -0.581 | 1.793 |
| Acetaminophen | Insulin (human) | 1.000 | 1.000 | 1.518 |  | 2.511 | 1.398 | 3.931 |  | 1.000 | 0.437 | 1.847 |  | 0.000 | -1.558 | 2.695 |
| Acetaminophen | Atorvastatin | 1.000 | 1.000 | 1.518 |  | 0.729 | 0.259 | 0.789 |  | 1.000 | 0.159 | 1.000 |  | 0.000 | -0.758 | 0.000 |
| Acetaminophen | Pitavastatin | 1.000 | 1.000 | 1.518 |  | 0.800 | 0.413 | 1.000 |  | 1.000 | 0.327 | 2.542 |  | 0.000 | -0.688 | 1.084 |
| Acetaminophen | Rosuvastatin | 1.000 | 1.000 | 1.518 |  | 0.685 | 0.203 | 0.698 |  | 2.244 | 1.000 | 6.903 |  | 0.852 | -0.122 | 2.373 |
| Acetaminophen | Allopurinol | 1.000 | 1.000 | 1.518 |  | 1.000 | 0.686 | 1.330 |  | 1.000 | 0.452 | 2.698 |  | 0.000 | -0.664 | 1.946 |
| Acetaminophen | Febuxostat | 1.000 | 1.000 | 1.518 |  | 0.626 | 0.226 | 0.986 |  | 1.000 | 0.330 | 2.419 |  | 0.000 | -0.631 | 0.586 |
| Acetaminophen | Alfacalcidol | 1.000 | 1.000 | 1.518 |  | 0.503 | 0.146 | 0.632 |  | 1.000 | 0.534 | 3.025 |  | 0.000 | -0.406 | 0.625 |
| Acetaminophen | Carbazochrome | 1.000 | 1.000 | 1.518 |  | 1.000 | 0.377 | 1.000 |  | 1.000 | 1.000 | 3.375 |  | 0.000 | -0.197 | 1.962 |
| Acetaminophen | Tranexamic acid | 1.000 | 1.000 | 1.518 |  | 0.446 | 0.263 | 0.796 |  | 1.000 | 0.701 | 1.691 |  | 0.000 | -0.330 | 0.281 |
| Acetaminophen | Edoxaban | 1.000 | 1.000 | 1.518 |  | 1.000 | 0.457 | 1.126 |  | 1.000 | 0.536 | 3.863 |  | 0.000 | -0.522 | 2.618 |
| Acetaminophen | Enoxaparin | 1.000 | 1.000 | 1.518 |  | 1.148 | 1.000 | 6.144 |  | 1.000 | 0.149 | 1.497 |  | 0.000 | -4.346 | 1.079 |

**Supplementary Table S7. Continued.**

| Drug 1 | Drug 2 | Adjusted odds ratio | | | | | | | | | | |  | Synergistic interaction | | |
| --- | --- | --- | --- | --- | --- | --- | --- | --- | --- | --- | --- | --- | --- | --- | --- | --- |
|  |  | Drug 1 | | |  | Drug 2 | | |  | Product term (Drug 1*Drug 2) | | |  |  |  |  |
|  |  | Estimate | 95%BootCI | |  | Estimate | 95%BootCI | |  | Estimate | 95%BootCI | |  | RERI | 95%BootCI | |
|  |  |  | Lower | Upper |  |  | Lower | Upper |  |  | Lower | Upper |  |  | Lower | Upper |
| Acetaminophen | Heparin sodium | 1.000 | 1.000 | 1.518 |  | 1.000 | 1.000 | 1.740 |  | 0.958 | 0.423 | 1.000 |  | -0.042 | -0.963 | 0.040 |
| Acetaminophen | Warfarin | 1.000 | 1.000 | 1.518 |  | 0.907 | 0.652 | 1.285 |  | 0.794 | 0.292 | 1.622 |  | -0.186 | -0.895 | 0.680 |
| Acetaminophen | Amlodipine | 1.000 | 1.000 | 1.518 |  | 0.797 | 0.467 | 0.994 |  | 1.000 | 0.472 | 1.222 |  | 0.000 | -0.543 | 0.132 |
| Acetaminophen | Olmesartan | 1.000 | 1.000 | 1.518 |  | 1.000 | 0.339 | 1.000 |  | 1.000 | 0.361 | 1.607 |  | 0.000 | -0.627 | 0.340 |
| Acetaminophen | Carvedilol | 1.000 | 1.000 | 1.518 |  | 1.000 | 0.765 | 1.536 |  | 1.000 | 0.552 | 2.907 |  | 0.000 | -0.546 | 2.350 |
| Acetaminophen | Candesartan | 1.000 | 1.000 | 1.518 |  | 0.854 | 0.400 | 1.000 |  | 1.000 | 0.600 | 3.187 |  | 0.000 | -0.426 | 1.644 |
| Acetaminophen | Telmisartan | 1.000 | 1.000 | 1.518 |  | 0.603 | 0.225 | 0.872 |  | 1.000 | 0.219 | 1.860 |  | 0.000 | -0.662 | 0.380 |
| Acetaminophen | Nicardipine | 1.000 | 1.000 | 1.518 |  | 1.000 | 0.643 | 2.532 |  | 1.000 | 0.298 | 3.019 |  | 0.000 | -1.466 | 2.318 |
| Acetaminophen | Nifedipine | 1.000 | 1.000 | 1.518 |  | 1.000 | 0.710 | 1.340 |  | 1.000 | 0.826 | 3.017 |  | 0.000 | -0.200 | 2.322 |
| Acetaminophen | Bisoprolol | 1.000 | 1.000 | 1.518 |  | 1.000 | 0.621 | 1.236 |  | 1.000 | 0.409 | 1.832 |  | 0.000 | -0.709 | 0.901 |
| Acetaminophen | Furosemide | 1.000 | 1.000 | 1.518 |  | 1.204 | 1.079 | 2.334 |  | 0.941 | 0.217 | 1.000 |  | -0.070 | -1.626 | 0.048 |
| Acetaminophen | Nicorandil | 1.000 | 1.000 | 1.518 |  | 1.000 | 0.861 | 2.193 |  | 1.499 | 1.000 | 11.798 |  | 0.499 | 0.000 | 16.115 |
| Acetaminophen | Ambroxol | 1.000 | 1.000 | 1.518 |  | 1.000 | 0.702 | 1.258 |  | 1.540 | 1.075 | 4.829 |  | 0.540 | 0.087 | 4.625 |
| Acetaminophen | L-carbocisteine | 1.000 | 1.000 | 1.518 |  | 1.000 | 0.595 | 1.000 |  | 1.000 | 0.635 | 1.605 |  | 0.000 | -0.418 | 0.556 |
| Acetaminophen | Dextromethorphan | 1.000 | 1.000 | 1.518 |  | 1.000 | 0.549 | 1.173 |  | 1.000 | 0.192 | 1.034 |  | 0.000 | -1.027 | 0.019 |
| Acetaminophen | Domperidone | 1.000 | 1.000 | 1.518 |  | 1.000 | 0.781 | 2.053 |  | 0.726 | 0.168 | 1.115 |  | -0.274 | -1.430 | 0.251 |
| Acetaminophen | Metoclopramide | 1.000 | 1.000 | 1.518 |  | 1.000 | 0.765 | 1.290 |  | 0.693 | 0.406 | 1.000 |  | -0.307 | -0.807 | 0.000 |
| Acetaminophen | Mosapride | 1.000 | 1.000 | 1.518 |  | 1.000 | 0.620 | 1.251 |  | 1.000 | 0.759 | 2.649 |  | 0.000 | -0.271 | 1.700 |
| Acetaminophen | Esomeprazole | 1.000 | 1.000 | 1.518 |  | 0.671 | 0.373 | 0.954 |  | 1.000 | 0.327 | 1.272 |  | 0.000 | -0.656 | 0.114 |
| Acetaminophen | Omeprazole | 1.000 | 1.000 | 1.518 |  | 1.045 | 0.928 | 1.644 |  | 1.000 | 0.649 | 2.805 |  | 0.000 | -0.449 | 2.337 |
| Acetaminophen | Teprenone | 1.000 | 1.000 | 1.518 |  | 0.605 | 0.480 | 1.000 |  | 1.000 | 0.826 | 2.620 |  | 0.000 | -0.237 | 1.232 |
| Acetaminophen | Famotidine | 1.000 | 1.000 | 1.518 |  | 1.643 | 1.332 | 2.071 |  | 1.176 | 1.000 | 2.333 |  | 0.290 | 0.050 | 2.541 |
| Acetaminophen | Vonoprazan | 1.000 | 1.000 | 1.518 |  | 1.000 | 0.392 | 1.000 |  | 1.119 | 1.000 | 6.166 |  | 0.119 | 0.000 | 3.598 |
| Acetaminophen | Ranitidine | 1.000 | 1.000 | 1.518 |  | 1.155 | 1.015 | 2.030 |  | 1.000 | 0.416 | 2.220 |  | 0.000 | -0.960 | 2.214 |
| Acetaminophen | Rabeprazole | 1.000 | 1.000 | 1.518 |  | 1.000 | 0.538 | 1.000 |  | 1.000 | 0.660 | 2.099 |  | 0.000 | -0.381 | 0.949 |
| Acetaminophen | Lansoprazole | 1.000 | 1.000 | 1.518 |  | 1.084 | 1.000 | 1.520 |  | 1.000 | 0.756 | 1.834 |  | 0.000 | -0.308 | 1.161 |
| Acetaminophen | Rebamipide | 1.000 | 1.000 | 1.518 |  | 0.739 | 0.345 | 0.760 |  | 1.000 | 1.000 | 2.353 |  | 0.000 | -0.191 | 0.724 |
| Acetaminophen | Magnesium oxide | 1.000 | 1.000 | 1.518 |  | 0.949 | 0.749 | 1.096 |  | 1.000 | 0.467 | 1.017 |  | 0.000 | -0.668 | 0.019 |
| Acetaminophen | Senna | 1.000 | 1.000 | 1.518 |  | 0.956 | 0.531 | 1.007 |  | 1.000 | 0.484 | 2.420 |  | 0.000 | -0.541 | 1.242 |
| Acetaminophen | Sennoside | 1.000 | 1.000 | 1.518 |  | 1.000 | 0.852 | 1.372 |  | 1.000 | 0.727 | 2.553 |  | 0.000 | -0.308 | 2.009 |
| Acetaminophen | Picosulfate | 1.000 | 1.000 | 1.518 |  | 1.072 | 1.000 | 2.023 |  | 1.000 | 0.420 | 1.699 |  | 0.000 | -0.894 | 1.193 |
| Acetaminophen | Haloperidol | 1.000 | 1.000 | 1.518 |  | 1.000 | 0.447 | 1.024 |  | 0.887 | 0.227 | 1.000 |  | -0.113 | -0.939 | 0.000 |
| Acetaminophen | Eszopiclone | 1.000 | 1.000 | 1.518 |  | 1.000 | 0.505 | 1.822 |  | 1.000 | 0.125 | 1.000 |  | 0.000 | -1.539 | 0.004 |
| Acetaminophen | Etizolam | 1.000 | 1.000 | 1.518 |  | 0.805 | 0.270 | 0.865 |  | 1.000 | 0.475 | 2.779 |  | 0.000 | -0.459 | 0.917 |
| Acetaminophen | Zolpidem | 1.000 | 1.000 | 1.518 |  | 1.000 | 0.767 | 1.233 |  | 1.000 | 0.601 | 1.775 |  | 0.000 | -0.491 | 0.883 |
| Acetaminophen | Brotizolam | 1.000 | 1.000 | 1.518 |  | 1.000 | 0.621 | 1.195 |  | 1.000 | 0.341 | 1.944 |  | 0.000 | -0.763 | 0.981 |
| Acetaminophen | Fentanyl | 1.000 | 1.000 | 1.518 |  | 4.031 | 3.726 | 8.961 |  | 0.748 | 0.169 | 1.000 |  | -1.014 | -6.193 | 0.435 |
| Acetaminophen | Buprenorphine | 1.000 | 1.000 | 1.518 |  | 1.000 | 0.998 | 7.643 |  | 1.000 | 1.000 | 8.334 |  | 0.000 | -0.051 | 17.551 |
| Acetaminophen | Pentazocine | 1.000 | 1.000 | 1.518 |  | 1.000 | 0.564 | 1.922 |  | 1.000 | 0.818 | 3.982 |  | 0.000 | -0.212 | 3.325 |
| Acetaminophen | Remifentanil | 1.000 | 1.000 | 1.518 |  | 1.000 | 0.473 | 1.000 |  | 0.885 | 0.251 | 1.255 |  | -0.115 | -0.891 | 0.209 |
| Acetaminophen | Midazolam | 1.000 | 1.000 | 1.518 |  | 1.000 | 0.814 | 1.575 |  | 1.000 | 0.410 | 2.558 |  | 0.000 | -0.718 | 2.076 |
| Acetaminophen | Daikenchuto | 1.000 | 1.000 | 1.518 |  | 1.000 | 0.925 | 1.936 |  | 1.000 | 0.276 | 1.147 |  | 0.000 | -1.170 | 0.240 |
| Etodolac | Heparin sodium | 0.638 | 0.336 | 1.000 |  | 1.000 | 1.000 | 1.740 |  | 1.000 | 0.254 | 2.080 |  | 0.000 | -0.890 | 0.678 |
| Etodolac | Irsogladine | 0.638 | 0.336 | 1.000 |  | 0.912 | 0.342 | 1.000 |  | 1.000 | 0.191 | 1.000 |  | 0.032 | -0.638 | 0.265 |
| Diclofenac | Celecoxib | 1.319 | 1.189 | 2.821 |  | 0.498 | 0.232 | 0.796 |  | 1.000 | 0.143 | 1.533 |  | -0.070 | -1.694 | 0.000 |
| Diclofenac | Loxoprofen | 1.319 | 1.189 | 2.821 |  | 0.824 | 0.611 | 1.000 |  | 1.000 | 0.381 | 1.263 |  | -0.025 | -1.381 | 0.269 |

**Supplementary Table S7. Continued.**

| Drug 1 | Drug 2 | Adjusted odds ratio | | | | | | | | | | |  | Synergistic interaction | | |
| --- | --- | --- | --- | --- | --- | --- | --- | --- | --- | --- | --- | --- | --- | --- | --- | --- |
|  |  | Drug 1 | | |  | Drug 2 | | |  | Product term (Drug 1*Drug 2) | | |  |  |  |  |
|  |  | Estimate | 95%BootCI | |  | Estimate | 95%BootCI | |  | Estimate | 95%BootCI | |  | RERI | 95%BootCI | |
|  |  |  | Lower | Upper |  |  | Lower | Upper |  |  | Lower | Upper |  |  | Lower | Upper |
| Diclofenac | Methylergometrine | 1.319 | 1.189 | 2.821 |  | 0.368 | 0.080 | 0.364 |  | 1.000 | 0.593 | 1.000 |  | -0.089 | -1.565 | -0.142 |
| Diclofenac | Tranexamic acid | 1.319 | 1.189 | 2.821 |  | 0.446 | 0.263 | 0.796 |  | 1.000 | 0.806 | 4.696 |  | -0.078 | -0.976 | 2.923 |
| Diclofenac | Heparin sodium | 1.319 | 1.189 | 2.821 |  | 1.000 | 1.000 | 1.740 |  | 1.000 | 0.404 | 2.008 |  | 0.000 | -1.294 | 3.013 |
| Diclofenac | Amlodipine | 1.319 | 1.189 | 2.821 |  | 0.797 | 0.467 | 0.994 |  | 1.206 | 0.748 | 3.548 |  | 0.158 | -0.781 | 2.686 |
| Diclofenac | Nifedipine | 1.319 | 1.189 | 2.821 |  | 1.000 | 0.710 | 1.340 |  | 1.000 | 0.452 | 3.454 |  | 0.000 | -1.145 | 4.839 |
| Diclofenac | Metoclopramide | 1.319 | 1.189 | 2.821 |  | 1.000 | 0.765 | 1.290 |  | 1.000 | 0.545 | 2.932 |  | 0.000 | -0.817 | 3.972 |
| Diclofenac | Teprenone | 1.319 | 1.189 | 2.821 |  | 0.605 | 0.480 | 1.000 |  | 1.000 | 0.191 | 0.916 |  | -0.056 | -1.958 | -0.228 |
| Diclofenac | Famotidine | 1.319 | 1.189 | 2.821 |  | 1.643 | 1.332 | 2.071 |  | 2.026 | 1.160 | 4.317 |  | 2.013 | 1.226 | 11.003 |
| Diclofenac | Misoprostol | 1.319 | 1.189 | 2.821 |  | 1.000 | 0.330 | 1.000 |  | 0.526 | 0.035 | 1.000 |  | -0.541 | -2.287 | -0.264 |
| Diclofenac | Lansoprazole | 1.319 | 1.189 | 2.821 |  | 1.084 | 1.000 | 1.520 |  | 1.000 | 0.326 | 1.985 |  | 0.012 | -1.511 | 2.597 |
| Diclofenac | Rebamipide | 1.319 | 1.189 | 2.821 |  | 0.739 | 0.345 | 0.760 |  | 1.000 | 0.496 | 1.683 |  | -0.037 | -1.280 | 0.247 |
| Diclofenac | Magnesium oxide | 1.319 | 1.189 | 2.821 |  | 0.949 | 0.749 | 1.096 |  | 1.000 | 0.347 | 1.206 |  | -0.007 | -1.481 | 0.287 |
| Diclofenac | Fentanyl | 1.319 | 1.189 | 2.821 |  | 4.031 | 3.726 | 8.961 |  | 1.000 | 0.206 | 1.085 |  | 0.426 | -4.958 | 8.038 |
| Serrapeptase | Loxoprofen | 1.000 | 0.339 | 1.000 |  | 0.824 | 0.611 | 1.000 |  | 1.000 | 0.124 | 1.000 |  | 0.000 | -0.714 | 0.179 |
| Serrapeptase | Methylergometrine | 1.000 | 0.339 | 1.000 |  | 0.368 | 0.080 | 0.364 |  | 1.000 | 0.648 | 2.201 |  | 0.000 | -0.035 | 0.588 |
| Serrapeptase | Teprenone | 1.000 | 0.339 | 1.000 |  | 0.605 | 0.480 | 1.000 |  | 1.000 | 0.509 | 3.174 |  | 0.000 | -0.295 | 0.941 |
| Celecoxib | Tramadol | 0.498 | 0.232 | 0.796 |  | 1.000 | 0.331 | 1.287 |  | 0.623 | 0.062 | 1.000 |  | -0.188 | -0.650 | 0.372 |
| Celecoxib | Pregabalin | 0.498 | 0.232 | 0.796 |  | 1.000 | 0.183 | 0.970 |  | 1.000 | 0.370 | 2.691 |  | 0.000 | -0.068 | 0.647 |
| Celecoxib | Loxoprofen | 0.498 | 0.232 | 0.796 |  | 0.824 | 0.611 | 1.000 |  | 1.000 | 0.980 | 6.302 |  | 0.088 | 0.000 | 2.221 |
| Celecoxib | Hydroxyzine | 0.498 | 0.232 | 0.796 |  | 1.000 | 0.545 | 1.272 |  | 1.000 | 0.170 | 1.023 |  | 0.000 | -0.498 | 0.250 |
| Celecoxib | Edoxaban | 0.498 | 0.232 | 0.796 |  | 1.000 | 0.457 | 1.126 |  | 1.000 | 0.693 | 2.821 |  | 0.000 | -0.137 | 0.718 |
| Celecoxib | Enoxaparin | 0.498 | 0.232 | 0.796 |  | 1.148 | 1.000 | 6.144 |  | 1.000 | 1.000 | 7.841 |  | -0.074 | -2.451 | 4.371 |
| Celecoxib | Fondaparinux | 0.498 | 0.232 | 0.796 |  | 1.000 | 0.445 | 2.724 |  | 1.000 | 1.000 | 10.250 |  | 0.000 | -0.663 | 3.054 |
| Celecoxib | Heparin sodium | 0.498 | 0.232 | 0.796 |  | 1.000 | 1.000 | 1.740 |  | 1.000 | 0.587 | 2.891 |  | 0.000 | -0.509 | 1.058 |
| Celecoxib | Amlodipine | 0.498 | 0.232 | 0.796 |  | 0.797 | 0.467 | 0.994 |  | 1.000 | 1.000 | 6.282 |  | 0.102 | 0.024 | 1.737 |
| Celecoxib | Metoclopramide | 0.498 | 0.232 | 0.796 |  | 1.000 | 0.765 | 1.290 |  | 1.000 | 0.484 | 1.931 |  | 0.000 | -0.318 | 0.335 |
| Celecoxib | Irsogladine | 0.498 | 0.232 | 0.796 |  | 0.912 | 0.342 | 1.000 |  | 1.000 | 0.235 | 1.019 |  | 0.044 | -0.348 | 0.389 |
| Celecoxib | Esomeprazole | 0.498 | 0.232 | 0.796 |  | 0.671 | 0.373 | 0.954 |  | 1.000 | 0.249 | 1.312 |  | 0.165 | -0.220 | 0.423 |
| Celecoxib | Teprenone | 0.498 | 0.232 | 0.796 |  | 0.605 | 0.480 | 1.000 |  | 1.000 | 0.336 | 1.609 |  | 0.198 | -0.244 | 0.390 |
| Celecoxib | Famotidine | 0.498 | 0.232 | 0.796 |  | 1.643 | 1.332 | 2.071 |  | 0.635 | 0.079 | 1.000 |  | -0.621 | -1.339 | -0.280 |
| Celecoxib | Misoprostol | 0.498 | 0.232 | 0.796 |  | 1.000 | 0.330 | 1.000 |  | 1.000 | 0.498 | 1.000 |  | 0.000 | -0.256 | 0.369 |
| Celecoxib | Rabeprazole | 0.498 | 0.232 | 0.796 |  | 1.000 | 0.538 | 1.000 |  | 1.000 | 0.323 | 2.976 |  | 0.000 | -0.234 | 0.804 |
| Celecoxib | Lansoprazole | 0.498 | 0.232 | 0.796 |  | 1.084 | 1.000 | 1.520 |  | 0.980 | 0.079 | 1.000 |  | -0.053 | -0.893 | 0.000 |
| Celecoxib | Rebamipide | 0.498 | 0.232 | 0.796 |  | 0.739 | 0.345 | 0.760 |  | 1.000 | 0.576 | 2.372 |  | 0.131 | 0.028 | 0.580 |
| Celecoxib | Magnesium oxide | 0.498 | 0.232 | 0.796 |  | 0.949 | 0.749 | 1.096 |  | 1.000 | 0.196 | 1.000 |  | 0.025 | -0.481 | 0.121 |
| Celecoxib | Sennoside | 0.498 | 0.232 | 0.796 |  | 1.000 | 0.852 | 1.372 |  | 0.452 | 0.034 | 1.000 |  | -0.273 | -0.794 | 0.000 |
| Celecoxib | Zolpidem | 0.498 | 0.232 | 0.796 |  | 1.000 | 0.767 | 1.233 |  | 1.000 | 0.134 | 1.000 |  | 0.000 | -0.563 | 0.104 |
| Celecoxib | Brotizolam | 0.498 | 0.232 | 0.796 |  | 1.000 | 0.621 | 1.195 |  | 1.000 | 0.182 | 1.343 |  | 0.000 | -0.452 | 0.248 |
| Tramadol | Loxoprofen | 1.000 | 0.331 | 1.287 |  | 0.824 | 0.611 | 1.000 |  | 1.000 | 0.134 | 1.005 |  | 0.000 | -0.808 | 0.157 |
| Tramadol | Metoclopramide | 1.000 | 0.331 | 1.287 |  | 1.000 | 0.765 | 1.290 |  | 1.000 | 0.342 | 2.399 |  | 0.000 | -0.647 | 1.230 |
| Tramadol | Rebamipide | 1.000 | 0.331 | 1.287 |  | 0.739 | 0.345 | 0.760 |  | 1.000 | 0.790 | 6.447 |  | 0.000 | -0.098 | 2.586 |
| Flurbiprofen | Loxoprofen | 1.000 | 0.509 | 1.123 |  | 0.824 | 0.611 | 1.000 |  | 1.000 | 0.279 | 2.391 |  | 0.000 | -0.601 | 0.913 |
| Flurbiprofen | Hydroxyzine | 1.000 | 0.509 | 1.123 |  | 1.000 | 0.545 | 1.272 |  | 1.000 | 0.652 | 2.135 |  | 0.000 | -0.310 | 0.888 |
| Flurbiprofen | Heparin sodium | 1.000 | 0.509 | 1.123 |  | 1.000 | 1.000 | 1.740 |  | 1.000 | 0.388 | 1.911 |  | 0.000 | -0.762 | 1.095 |
| Flurbiprofen | Metoclopramide | 1.000 | 0.509 | 1.123 |  | 1.000 | 0.765 | 1.290 |  | 1.000 | 0.403 | 1.056 |  | 0.000 | -0.612 | 0.070 |
| Flurbiprofen | Rebamipide | 1.000 | 0.509 | 1.123 |  | 0.739 | 0.345 | 0.760 |  | 1.000 | 0.227 | 2.925 |  | 0.000 | -0.402 | 0.832 |

**Supplementary Table S7. Continued.**

| Drug 1 | Drug 2 | Adjusted odds ratio | | | | | | | | | | |  | Synergistic interaction | | |
| --- | --- | --- | --- | --- | --- | --- | --- | --- | --- | --- | --- | --- | --- | --- | --- | --- |
|  |  | Drug 1 | | |  | Drug 2 | | |  | Product term (Drug 1*Drug 2) | | |  |  |  |  |
|  |  | Estimate | 95%BootCI | |  | Estimate | 95%BootCI | |  | Estimate | 95%BootCI | |  | RERI | 95%BootCI | |
|  |  |  | Lower | Upper |  |  | Lower | Upper |  |  | Lower | Upper |  |  | Lower | Upper |
| Flurbiprofen | Haloperidol | 1.000 | 0.509 | 1.123 |  | 1.000 | 0.447 | 1.024 |  | 1.349 | 1.000 | 6.671 |  | 0.349 | 0.000 | 4.303 |
| Pregabalin | Loxoprofen | 1.000 | 0.183 | 0.970 |  | 0.824 | 0.611 | 1.000 |  | 1.000 | 0.571 | 5.123 |  | 0.000 | -0.112 | 1.246 |
| Pregabalin | Rebamipide | 1.000 | 0.183 | 0.970 |  | 0.739 | 0.345 | 0.760 |  | 1.000 | 0.159 | 1.340 |  | 0.000 | -0.218 | 0.486 |
| Pregabalin | Magnesium oxide | 1.000 | 0.183 | 0.970 |  | 0.949 | 0.749 | 1.096 |  | 1.195 | 1.000 | 10.263 |  | 0.185 | 0.000 | 3.290 |
| Loxoprofen | Chlorpheniramine | 0.824 | 0.611 | 1.000 |  | 0.920 | 0.571 | 1.688 |  | 1.000 | 0.610 | 3.523 |  | 0.014 | -0.337 | 1.900 |
| Loxoprofen | Olopatadine | 0.824 | 0.611 | 1.000 |  | 1.000 | 0.575 | 1.169 |  | 1.000 | 0.600 | 2.250 |  | 0.000 | -0.335 | 0.872 |
| Loxoprofen | Hydroxyzine | 0.824 | 0.611 | 1.000 |  | 1.000 | 0.545 | 1.272 |  | 1.000 | 0.205 | 1.000 |  | 0.000 | -0.665 | 0.108 |
| Loxoprofen | Atorvastatin | 0.824 | 0.611 | 1.000 |  | 0.729 | 0.259 | 0.789 |  | 1.000 | 0.293 | 3.104 |  | 0.048 | -0.264 | 0.876 |
| Loxoprofen | Rosuvastatin | 0.824 | 0.611 | 1.000 |  | 0.685 | 0.203 | 0.698 |  | 1.238 | 1.000 | 10.778 |  | 0.190 | 0.030 | 3.192 |
| Loxoprofen | Allopurinol | 0.824 | 0.611 | 1.000 |  | 1.000 | 0.686 | 1.330 |  | 1.000 | 0.480 | 2.738 |  | 0.000 | -0.425 | 1.315 |
| Loxoprofen | Alfacalcidol | 0.824 | 0.611 | 1.000 |  | 0.503 | 0.146 | 0.632 |  | 1.000 | 0.344 | 2.852 |  | 0.088 | -0.157 | 0.561 |
| Loxoprofen | Carbazochrome | 0.824 | 0.611 | 1.000 |  | 1.000 | 0.377 | 1.000 |  | 1.000 | 0.449 | 1.682 |  | 0.000 | -0.335 | 0.458 |
| Loxoprofen | Tranexamic acid | 0.824 | 0.611 | 1.000 |  | 0.446 | 0.263 | 0.796 |  | 1.000 | 0.874 | 1.972 |  | 0.098 | 0.000 | 0.475 |
| Loxoprofen | Heparin sodium | 0.824 | 0.611 | 1.000 |  | 1.000 | 1.000 | 1.740 |  | 0.837 | 0.329 | 1.000 |  | -0.134 | -0.931 | -0.035 |
| Loxoprofen | Warfarin | 0.824 | 0.611 | 1.000 |  | 0.907 | 0.652 | 1.285 |  | 2.496 | 1.000 | 5.695 |  | 1.134 | 0.000 | 3.619 |
| Loxoprofen | Amlodipine | 0.824 | 0.611 | 1.000 |  | 0.797 | 0.467 | 0.994 |  | 1.000 | 0.799 | 2.308 |  | 0.036 | -0.090 | 0.760 |
| Loxoprofen | Olmesartan | 0.824 | 0.611 | 1.000 |  | 1.000 | 0.339 | 1.000 |  | 1.300 | 0.899 | 5.435 |  | 0.247 | -0.001 | 2.177 |
| Loxoprofen | Candesartan | 0.824 | 0.611 | 1.000 |  | 0.854 | 0.400 | 1.000 |  | 1.000 | 0.535 | 3.306 |  | 0.026 | -0.243 | 1.342 |
| Loxoprofen | Nifedipine | 0.824 | 0.611 | 1.000 |  | 1.000 | 0.710 | 1.340 |  | 1.000 | 0.280 | 1.581 |  | 0.000 | -0.642 | 0.498 |
| Loxoprofen | Bisoprolol | 0.824 | 0.611 | 1.000 |  | 1.000 | 0.621 | 1.236 |  | 1.000 | 0.178 | 1.447 |  | 0.000 | -0.735 | 0.329 |
| Loxoprofen | Furosemide | 0.824 | 0.611 | 1.000 |  | 1.204 | 1.079 | 2.334 |  | 1.021 | 0.865 | 3.441 |  | -0.015 | -0.340 | 3.202 |
| Loxoprofen | Ambroxol | 0.824 | 0.611 | 1.000 |  | 1.000 | 0.702 | 1.258 |  | 1.000 | 0.437 | 1.395 |  | 0.000 | -0.513 | 0.314 |
| Loxoprofen | L-carbocisteine | 0.824 | 0.611 | 1.000 |  | 1.000 | 0.595 | 1.000 |  | 0.945 | 0.635 | 1.350 |  | -0.045 | -0.288 | 0.274 |
| Loxoprofen | Dextromethorphan | 0.824 | 0.611 | 1.000 |  | 1.000 | 0.549 | 1.173 |  | 1.000 | 0.250 | 2.838 |  | 0.000 | -0.586 | 1.259 |
| Loxoprofen | Metoclopramide | 0.824 | 0.611 | 1.000 |  | 1.000 | 0.765 | 1.290 |  | 0.765 | 0.281 | 1.000 |  | -0.194 | -0.654 | 0.025 |
| Loxoprofen | Esomeprazole | 0.824 | 0.611 | 1.000 |  | 0.671 | 0.373 | 0.954 |  | 1.000 | 0.533 | 2.181 |  | 0.058 | -0.238 | 0.579 |
| Loxoprofen | Omeprazole | 0.824 | 0.611 | 1.000 |  | 1.045 | 0.928 | 1.644 |  | 1.000 | 0.403 | 1.811 |  | -0.008 | -0.684 | 0.692 |
| Loxoprofen | Teprenone | 0.824 | 0.611 | 1.000 |  | 0.605 | 0.480 | 1.000 |  | 1.000 | 0.449 | 1.000 |  | 0.070 | -0.438 | 0.123 |
| Loxoprofen | Famotidine | 0.824 | 0.611 | 1.000 |  | 1.643 | 1.332 | 2.071 |  | 1.000 | 0.673 | 1.545 |  | -0.113 | -0.620 | 0.593 |
| Loxoprofen | Vonoprazan | 0.824 | 0.611 | 1.000 |  | 1.000 | 0.392 | 1.000 |  | 1.000 | 0.271 | 1.208 |  | 0.000 | -0.510 | 0.191 |
| Loxoprofen | Misoprostol | 0.824 | 0.611 | 1.000 |  | 1.000 | 0.330 | 1.000 |  | 1.000 | 0.161 | 1.000 |  | 0.000 | -0.699 | 0.149 |
| Loxoprofen | Ranitidine | 0.824 | 0.611 | 1.000 |  | 1.155 | 1.015 | 2.030 |  | 0.579 | 0.151 | 0.820 |  | -0.428 | -1.418 | -0.251 |
| Loxoprofen | Rabeprazole | 0.824 | 0.611 | 1.000 |  | 1.000 | 0.538 | 1.000 |  | 1.000 | 0.723 | 2.095 |  | 0.000 | -0.153 | 0.696 |
| Loxoprofen | Lansoprazole | 0.824 | 0.611 | 1.000 |  | 1.084 | 1.000 | 1.520 |  | 1.000 | 0.404 | 1.132 |  | -0.015 | -0.723 | 0.096 |
| Loxoprofen | Rebamipide | 0.824 | 0.611 | 1.000 |  | 0.739 | 0.345 | 0.760 |  | 1.000 | 0.679 | 1.157 |  | 0.046 | -0.162 | 0.207 |
| Loxoprofen | Magnesium oxide | 0.824 | 0.611 | 1.000 |  | 0.949 | 0.749 | 1.096 |  | 1.000 | 0.646 | 1.602 |  | 0.009 | -0.306 | 0.461 |
| Loxoprofen | Senna | 0.824 | 0.611 | 1.000 |  | 0.956 | 0.531 | 1.007 |  | 1.000 | 0.678 | 2.787 |  | 0.008 | -0.236 | 1.202 |
| Loxoprofen | Sennoside | 0.824 | 0.611 | 1.000 |  | 1.000 | 0.852 | 1.372 |  | 1.000 | 0.385 | 1.459 |  | 0.000 | -0.604 | 0.381 |
| Loxoprofen | Picosulfate | 0.824 | 0.611 | 1.000 |  | 1.072 | 1.000 | 2.023 |  | 1.000 | 0.397 | 1.913 |  | -0.013 | -0.860 | 0.853 |
| Loxoprofen | Haloperidol | 0.824 | 0.611 | 1.000 |  | 1.000 | 0.447 | 1.024 |  | 1.000 | 0.327 | 1.336 |  | 0.000 | -0.508 | 0.264 |
| Loxoprofen | Etizolam | 0.824 | 0.611 | 1.000 |  | 0.805 | 0.270 | 0.865 |  | 1.000 | 0.498 | 2.932 |  | 0.034 | -0.196 | 0.847 |
| Loxoprofen | Zopiclone | 0.824 | 0.611 | 1.000 |  | 1.000 | 0.681 | 1.535 |  | 1.000 | 0.879 | 5.499 |  | 0.000 | -0.114 | 3.459 |
| Loxoprofen | Zolpidem | 0.824 | 0.611 | 1.000 |  | 1.000 | 0.767 | 1.233 |  | 1.000 | 0.599 | 2.102 |  | 0.000 | -0.348 | 0.901 |
| Loxoprofen | Brotizolam | 0.824 | 0.611 | 1.000 |  | 1.000 | 0.621 | 1.195 |  | 1.597 | 1.000 | 5.505 |  | 0.492 | 0.000 | 3.617 |
| Loxoprofen | Eperisone | 0.824 | 0.611 | 1.000 |  | 0.548 | 0.076 | 0.529 |  | 1.000 | 0.635 | 3.562 |  | 0.080 | -0.022 | 0.518 |
| Loxoprofen | Fentanyl | 0.824 | 0.611 | 1.000 |  | 4.031 | 3.726 | 8.961 |  | 1.000 | 0.459 | 1.481 |  | -0.534 | -4.299 | 1.268 |

**Supplementary Table S7. Continued.**

| Drug 1 | Drug 2 | Adjusted odds ratio | | | | | | | | | | |  | Synergistic interaction | | |
| --- | --- | --- | --- | --- | --- | --- | --- | --- | --- | --- | --- | --- | --- | --- | --- | --- |
|  |  | Drug 1 | | |  | Drug 2 | | |  | Product term (Drug 1*Drug 2) | | |  |  |  |  |
|  |  | Estimate | 95%BootCI | |  | Estimate | 95%BootCI | |  | Estimate | 95%BootCI | |  | RERI | 95%BootCI | |
|  |  |  | Lower | Upper |  |  | Lower | Upper |  |  | Lower | Upper |  |  | Lower | Upper |
| Loxoprofen | Remifentanil | 0.824 | 0.611 | 1.000 |  | 1.000 | 0.473 | 1.000 |  | 1.000 | 0.465 | 2.009 |  | 0.000 | -0.396 | 0.697 |
| Loxoprofen | Midazolam | 0.824 | 0.611 | 1.000 |  | 1.000 | 0.814 | 1.575 |  | 0.981 | 0.304 | 1.000 |  | -0.016 | -0.712 | 0.018 |
| Loxoprofen | Daikenchuto | 0.824 | 0.611 | 1.000 |  | 1.000 | 0.925 | 1.936 |  | 1.000 | 0.540 | 4.187 |  | 0.000 | -0.551 | 3.212 |
| Lornoxicam | Heparin sodium | 0.518 | 0.151 | 0.868 |  | 1.000 | 1.000 | 1.740 |  | 1.000 | 0.647 | 4.410 |  | 0.000 | -0.488 | 1.102 |
| Lornoxicam | Ambroxol | 0.518 | 0.151 | 0.868 |  | 1.000 | 0.702 | 1.258 |  | 1.000 | 0.426 | 4.123 |  | 0.000 | -0.323 | 0.814 |
| Lornoxicam | Teprenone | 0.518 | 0.151 | 0.868 |  | 0.605 | 0.480 | 1.000 |  | 1.000 | 0.239 | 1.000 |  | 0.191 | -0.381 | 0.336 |
| Lornoxicam | Rebamipide | 0.518 | 0.151 | 0.868 |  | 0.739 | 0.345 | 0.760 |  | 1.000 | 0.482 | 2.854 |  | 0.126 | -0.010 | 0.616 |
| Chlorpheniramine | Olopatadine | 0.920 | 0.571 | 1.688 |  | 1.000 | 0.575 | 1.169 |  | 1.000 | 0.551 | 2.938 |  | 0.000 | -0.501 | 1.400 |
| Chlorpheniramine | Heparin sodium | 0.920 | 0.571 | 1.688 |  | 1.000 | 1.000 | 1.740 |  | 0.704 | 0.223 | 1.099 |  | -0.272 | -1.228 | 0.195 |
| Chlorpheniramine | Ranitidine | 0.920 | 0.571 | 1.688 |  | 1.155 | 1.015 | 2.030 |  | 0.887 | 0.165 | 1.449 |  | -0.133 | -1.525 | 0.667 |
| Chlorpheniramine | Magnesium oxide | 0.920 | 0.571 | 1.688 |  | 0.949 | 0.749 | 1.096 |  | 1.000 | 0.148 | 1.539 |  | 0.004 | -0.970 | 0.518 |
| Olopatadine | Carbazochrome | 1.000 | 0.575 | 1.169 |  | 1.000 | 0.377 | 1.000 |  | 1.000 | 0.966 | 3.397 |  | 0.000 | -0.100 | 1.568 |
| Olopatadine | Tranexamic acid | 1.000 | 0.575 | 1.169 |  | 0.446 | 0.263 | 0.796 |  | 1.000 | 0.417 | 1.511 |  | 0.000 | -0.308 | 0.309 |
| Olopatadine | Heparin sodium | 1.000 | 0.575 | 1.169 |  | 1.000 | 1.000 | 1.740 |  | 0.937 | 0.327 | 1.373 |  | -0.063 | -0.932 | 0.426 |
| Olopatadine | L-carbocisteine | 1.000 | 0.575 | 1.169 |  | 1.000 | 0.595 | 1.000 |  | 0.906 | 0.137 | 1.000 |  | -0.094 | -0.821 | 0.070 |
| Olopatadine | Metoclopramide | 1.000 | 0.575 | 1.169 |  | 1.000 | 0.765 | 1.290 |  | 1.000 | 0.245 | 1.000 |  | 0.000 | -0.744 | 0.043 |
| Olopatadine | Teprenone | 1.000 | 0.575 | 1.169 |  | 0.605 | 0.480 | 1.000 |  | 1.000 | 0.287 | 2.420 |  | 0.000 | -0.529 | 0.882 |
| Olopatadine | Rebamipide | 1.000 | 0.575 | 1.169 |  | 0.739 | 0.345 | 0.760 |  | 1.000 | 0.248 | 1.535 |  | 0.000 | -0.409 | 0.314 |
| Diphenhydramine | Diazepam | 1.788 | 1.000 | 6.845 |  | 1.000 | 0.539 | 1.445 |  | 1.000 | 0.291 | 2.161 |  | 0.000 | -4.589 | 1.385 |
| Hydroxyzine | Insulin (human) | 1.000 | 0.545 | 1.272 |  | 2.511 | 1.398 | 3.931 |  | 1.000 | 0.480 | 4.180 |  | 0.000 | -1.345 | 7.363 |
| Hydroxyzine | Tranexamic acid | 1.000 | 0.545 | 1.272 |  | 0.446 | 0.263 | 0.796 |  | 1.000 | 0.737 | 6.185 |  | 0.000 | -0.168 | 2.371 |
| Hydroxyzine | Heparin sodium | 1.000 | 0.545 | 1.272 |  | 1.000 | 1.000 | 1.740 |  | 0.883 | 0.382 | 1.096 |  | -0.117 | -0.895 | 0.143 |
| Hydroxyzine | Amlodipine | 1.000 | 0.545 | 1.272 |  | 0.797 | 0.467 | 0.994 |  | 1.000 | 0.582 | 2.920 |  | 0.000 | -0.236 | 1.212 |
| Hydroxyzine | Metoclopramide | 1.000 | 0.545 | 1.272 |  | 1.000 | 0.765 | 1.290 |  | 0.853 | 0.292 | 1.000 |  | -0.147 | -0.818 | 0.000 |
| Hydroxyzine | Famotidine | 1.000 | 0.545 | 1.272 |  | 1.643 | 1.332 | 2.071 |  | 0.590 | 0.141 | 0.848 |  | -0.673 | -1.626 | -0.301 |
| Hydroxyzine | Lansoprazole | 1.000 | 0.545 | 1.272 |  | 1.084 | 1.000 | 1.520 |  | 1.000 | 0.294 | 1.989 |  | 0.000 | -0.890 | 0.952 |
| Hydroxyzine | Rebamipide | 1.000 | 0.545 | 1.272 |  | 0.739 | 0.345 | 0.760 |  | 1.000 | 0.761 | 3.228 |  | 0.000 | -0.178 | 1.043 |
| Hydroxyzine | Magnesium oxide | 1.000 | 0.545 | 1.272 |  | 0.949 | 0.749 | 1.096 |  | 1.000 | 0.356 | 2.458 |  | 0.000 | -0.613 | 1.205 |
| Hydroxyzine | Haloperidol | 1.000 | 0.545 | 1.272 |  | 1.000 | 0.447 | 1.024 |  | 1.000 | 1.000 | 4.445 |  | 0.000 | 0.000 | 2.236 |
| Hydroxyzine | Zolpidem | 1.000 | 0.545 | 1.272 |  | 1.000 | 0.767 | 1.233 |  | 1.000 | 0.195 | 1.411 |  | 0.000 | -0.814 | 0.347 |
| Hydroxyzine | Buprenorphine | 1.000 | 0.545 | 1.272 |  | 1.000 | 0.998 | 7.643 |  | 1.000 | 0.038 | 1.000 |  | 0.000 | -4.703 | 0.139 |
| Hydroxyzine | Pentazocine | 1.000 | 0.545 | 1.272 |  | 1.000 | 0.564 | 1.922 |  | 1.000 | 1.000 | 4.300 |  | 0.000 | -0.031 | 2.692 |
| Fexofenadine | Heparin sodium | 1.000 | 0.598 | 1.549 |  | 1.000 | 1.000 | 1.740 |  | 1.000 | 0.370 | 3.348 |  | 0.000 | -0.812 | 2.850 |
| Pranlukast | L-carbocisteine | 0.628 | 0.216 | 1.000 |  | 1.000 | 0.595 | 1.000 |  | 1.000 | 0.166 | 1.280 |  | 0.000 | -0.709 | 0.271 |
| Montelukast | Theophylline | 0.723 | 0.161 | 1.000 |  | 0.904 | 0.340 | 1.070 |  | 1.000 | 0.349 | 4.967 |  | 0.026 | -0.404 | 1.006 |
| Montelukast | L-carbocisteine | 0.723 | 0.161 | 1.000 |  | 1.000 | 0.595 | 1.000 |  | 0.993 | 0.109 | 1.289 |  | -0.005 | -0.670 | 0.275 |
| Imidapril | Carvedilol | 0.747 | 0.245 | 1.000 |  | 1.000 | 0.765 | 1.536 |  | 1.000 | 0.353 | 2.221 |  | 0.000 | -0.615 | 0.555 |
| Imidapril | Furosemide | 0.747 | 0.245 | 1.000 |  | 1.204 | 1.079 | 2.334 |  | 1.000 | 0.308 | 2.175 |  | -0.052 | -1.150 | 0.655 |
| Insulin (human) | Heparin sodium | 2.511 | 1.398 | 3.931 |  | 1.000 | 1.000 | 1.740 |  | 0.912 | 0.306 | 1.022 |  | -0.222 | -2.211 | 0.797 |
| Insulin (human) | Amlodipine | 2.511 | 1.398 | 3.931 |  | 0.797 | 0.467 | 0.994 |  | 0.961 | 0.464 | 2.187 |  | -0.385 | -1.817 | 1.513 |
| Insulin (human) | Furosemide | 2.511 | 1.398 | 3.931 |  | 1.204 | 1.079 | 2.334 |  | 1.000 | 0.540 | 2.479 |  | 0.308 | -0.980 | 7.109 |
| Insulin (human) | L-carbocisteine | 2.511 | 1.398 | 3.931 |  | 1.000 | 0.595 | 1.000 |  | 0.403 | 0.128 | 0.936 |  | -1.498 | -2.879 | -0.245 |
| Insulin (human) | Metoclopramide | 2.511 | 1.398 | 3.931 |  | 1.000 | 0.765 | 1.290 |  | 1.151 | 1.000 | 5.546 |  | 0.380 | -0.147 | 10.719 |
| Insulin (human) | Famotidine | 2.511 | 1.398 | 3.931 |  | 1.643 | 1.332 | 2.071 |  | 0.934 | 0.337 | 1.156 |  | 0.700 | -2.088 | 1.574 |
| Insulin (human) | Lansoprazole | 2.511 | 1.398 | 3.931 |  | 1.084 | 1.000 | 1.520 |  | 0.803 | 0.308 | 1.320 |  | -0.408 | -2.113 | 1.164 |
| Insulin (human) | Haloperidol | 2.511 | 1.398 | 3.931 |  | 1.000 | 0.447 | 1.024 |  | 1.000 | 0.269 | 2.044 |  | 0.000 | -2.076 | 1.953 |

**Supplementary Table S7. Continued.**

| Drug 1 | Drug 2 | Adjusted odds ratio | | | | | | | | | | |  | Synergistic interaction | | |
| --- | --- | --- | --- | --- | --- | --- | --- | --- | --- | --- | --- | --- | --- | --- | --- | --- |
|  |  | Drug 1 | | |  | Drug 2 | | |  | Product term (Drug 1*Drug 2) | | |  |  |  |  |
|  |  | Estimate | 95%BootCI | |  | Estimate | 95%BootCI | |  | Estimate | 95%BootCI | |  | RERI | 95%BootCI | |
|  |  |  | Lower | Upper |  |  | Lower | Upper |  |  | Lower | Upper |  |  | Lower | Upper |
| Insulin (human) | Fentanyl | 2.511 | 1.398 | 3.931 |  | 4.031 | 3.726 | 8.961 |  | 0.862 | 0.452 | 2.410 |  | 3.185 | -0.839 | 34.133 |
| Glimepiride | Sitagliptin | 0.821 | 0.290 | 1.000 |  | 0.590 | 0.229 | 1.000 |  | 1.000 | 0.621 | 7.165 |  | 0.074 | -0.070 | 1.418 |
| Glimepiride | Pioglitazone | 0.821 | 0.290 | 1.000 |  | 0.914 | 0.127 | 0.989 |  | 1.000 | 1.000 | 14.973 |  | 0.015 | 0.002 | 2.150 |
| Glimepiride | Voglibose | 0.821 | 0.290 | 1.000 |  | 1.000 | 0.538 | 1.446 |  | 1.000 | 0.228 | 2.078 |  | 0.000 | -0.618 | 0.541 |
| Glimepiride | Metformin | 0.821 | 0.290 | 1.000 |  | 0.500 | 0.228 | 0.948 |  | 1.000 | 0.126 | 1.123 |  | 0.090 | -0.383 | 0.406 |
| Glimepiride | Amlodipine | 0.821 | 0.290 | 1.000 |  | 0.797 | 0.467 | 0.994 |  | 1.000 | 0.285 | 1.959 |  | 0.036 | -0.346 | 0.488 |
| Sitagliptin | Metformin | 0.590 | 0.229 | 1.000 |  | 0.500 | 0.228 | 0.948 |  | 0.890 | 0.030 | 1.000 |  | 0.173 | -0.450 | 0.454 |
| Sitagliptin | Amlodipine | 0.590 | 0.229 | 1.000 |  | 0.797 | 0.467 | 0.994 |  | 1.000 | 0.197 | 2.502 |  | 0.083 | -0.340 | 0.595 |
| Voglibose | Amlodipine | 1.000 | 0.538 | 1.446 |  | 0.797 | 0.467 | 0.994 |  | 1.000 | 0.479 | 3.687 |  | 0.000 | -0.409 | 1.523 |
| Metformin | Amlodipine | 0.500 | 0.228 | 0.948 |  | 0.797 | 0.467 | 0.994 |  | 1.000 | 0.145 | 1.000 |  | 0.102 | -0.402 | 0.337 |
| Losartan | Amlodipine | 0.776 | 0.341 | 1.000 |  | 0.797 | 0.467 | 0.994 |  | 0.706 | 0.088 | 1.000 |  | -0.136 | -0.641 | 0.207 |
| Losartan | Furosemide | 0.776 | 0.341 | 1.000 |  | 1.204 | 1.079 | 2.334 |  | 1.000 | 0.104 | 1.000 |  | -0.046 | -1.679 | -0.095 |
| Atorvastatin | Allopurinol | 0.729 | 0.259 | 0.789 |  | 1.000 | 0.686 | 1.330 |  | 1.000 | 0.265 | 2.277 |  | 0.000 | -0.411 | 0.535 |
| Atorvastatin | Alfacalcidol | 0.729 | 0.259 | 0.789 |  | 0.503 | 0.146 | 0.632 |  | 1.000 | 0.221 | 5.674 |  | 0.135 | 0.082 | 1.061 |
| Atorvastatin | Clopidogrel | 0.729 | 0.259 | 0.789 |  | 0.808 | 0.309 | 1.000 |  | 1.000 | 0.976 | 5.918 |  | 0.052 | 0.000 | 1.434 |
| Atorvastatin | Heparin sodium | 0.729 | 0.259 | 0.789 |  | 1.000 | 1.000 | 1.740 |  | 1.000 | 1.000 | 4.633 |  | 0.000 | -0.260 | 1.785 |
| Atorvastatin | Amlodipine | 0.729 | 0.259 | 0.789 |  | 0.797 | 0.467 | 0.994 |  | 1.000 | 0.466 | 1.724 |  | 0.055 | -0.158 | 0.423 |
| Atorvastatin | Olmesartan | 0.729 | 0.259 | 0.789 |  | 1.000 | 0.339 | 1.000 |  | 1.000 | 0.574 | 3.783 |  | 0.000 | -0.028 | 0.930 |
| Atorvastatin | Carvedilol | 0.729 | 0.259 | 0.789 |  | 1.000 | 0.765 | 1.536 |  | 1.000 | 0.649 | 3.292 |  | 0.000 | -0.296 | 1.036 |
| Atorvastatin | Candesartan | 0.729 | 0.259 | 0.789 |  | 0.854 | 0.400 | 1.000 |  | 1.000 | 0.128 | 1.000 |  | 0.040 | -0.396 | 0.337 |
| Atorvastatin | Nifedipine | 0.729 | 0.259 | 0.789 |  | 1.000 | 0.710 | 1.340 |  | 1.000 | 0.543 | 2.898 |  | 0.000 | -0.280 | 0.750 |
| Atorvastatin | Furosemide | 0.729 | 0.259 | 0.789 |  | 1.204 | 1.079 | 2.334 |  | 1.000 | 0.293 | 1.219 |  | -0.055 | -1.174 | -0.034 |
| Atorvastatin | Nicorandil | 0.729 | 0.259 | 0.789 |  | 1.000 | 0.861 | 2.193 |  | 1.000 | 0.196 | 1.383 |  | 0.000 | -1.091 | 0.168 |
| Atorvastatin | Famotidine | 0.729 | 0.259 | 0.789 |  | 1.643 | 1.332 | 2.071 |  | 1.000 | 0.511 | 3.385 |  | -0.175 | -0.820 | 1.289 |
| Atorvastatin | Rabeprazole | 0.729 | 0.259 | 0.789 |  | 1.000 | 0.538 | 1.000 |  | 1.000 | 0.334 | 1.686 |  | 0.000 | -0.244 | 0.385 |
| Atorvastatin | Lansoprazole | 0.729 | 0.259 | 0.789 |  | 1.084 | 1.000 | 1.520 |  | 1.000 | 0.438 | 2.037 |  | -0.023 | -0.510 | 0.448 |
| Atorvastatin | Rebamipide | 0.729 | 0.259 | 0.789 |  | 0.739 | 0.345 | 0.760 |  | 1.000 | 0.167 | 1.425 |  | 0.071 | -0.146 | 0.450 |
| Atorvastatin | Magnesium oxide | 0.729 | 0.259 | 0.789 |  | 0.949 | 0.749 | 1.096 |  | 1.000 | 0.489 | 2.665 |  | 0.014 | -0.211 | 0.719 |
| Atorvastatin | Zolpidem | 0.729 | 0.259 | 0.789 |  | 1.000 | 0.767 | 1.233 |  | 1.000 | 0.182 | 1.612 |  | 0.000 | -0.461 | 0.286 |
| Tocopheryl nicotinate | Amlodipine | 0.848 | 0.186 | 0.817 |  | 0.797 | 0.467 | 0.994 |  | 1.000 | 1.000 | 12.140 |  | 0.031 | 0.043 | 2.485 |
| Pitavastatin | Clopidogrel | 0.800 | 0.413 | 1.000 |  | 0.808 | 0.309 | 1.000 |  | 1.000 | 0.254 | 2.349 |  | 0.038 | -0.468 | 0.635 |
| Pitavastatin | Heparin sodium | 0.800 | 0.413 | 1.000 |  | 1.000 | 1.000 | 1.740 |  | 1.000 | 0.287 | 2.153 |  | 0.000 | -0.863 | 0.990 |
| Pitavastatin | Amlodipine | 0.800 | 0.413 | 1.000 |  | 0.797 | 0.467 | 0.994 |  | 1.000 | 0.433 | 2.496 |  | 0.041 | -0.299 | 0.747 |
| Pitavastatin | Olmesartan | 0.800 | 0.413 | 1.000 |  | 1.000 | 0.339 | 1.000 |  | 1.000 | 0.152 | 1.772 |  | 0.000 | -0.529 | 0.439 |
| Pitavastatin | Furosemide | 0.800 | 0.413 | 1.000 |  | 1.204 | 1.079 | 2.334 |  | 1.000 | 0.301 | 3.351 |  | -0.041 | -1.087 | 2.550 |
| Pitavastatin | Rabeprazole | 0.800 | 0.413 | 1.000 |  | 1.000 | 0.538 | 1.000 |  | 1.000 | 0.610 | 3.322 |  | 0.000 | -0.205 | 1.341 |
| Pitavastatin | Lansoprazole | 0.800 | 0.413 | 1.000 |  | 1.084 | 1.000 | 1.520 |  | 1.000 | 0.159 | 1.323 |  | -0.017 | -0.886 | 0.220 |
| Pitavastatin | Magnesium oxide | 0.800 | 0.413 | 1.000 |  | 0.949 | 0.749 | 1.096 |  | 1.000 | 0.187 | 1.914 |  | 0.010 | -0.684 | 0.632 |
| Pravastatin | Heparin sodium | 0.739 | 0.208 | 0.768 |  | 1.000 | 1.000 | 1.740 |  | 1.000 | 0.730 | 4.413 |  | 0.000 | -0.416 | 1.523 |
| Pravastatin | Amlodipine | 0.739 | 0.208 | 0.768 |  | 0.797 | 0.467 | 0.994 |  | 1.000 | 0.807 | 3.904 |  | 0.053 | 0.000 | 0.813 |
| Pravastatin | Magnesium oxide | 0.739 | 0.208 | 0.768 |  | 0.949 | 0.749 | 1.096 |  | 0.848 | 0.052 | 1.000 |  | -0.094 | -0.599 | 0.094 |
| Rosuvastatin | Febuxostat | 0.685 | 0.203 | 0.698 |  | 0.626 | 0.226 | 0.986 |  | 1.000 | 0.761 | 4.999 |  | 0.118 | 0.036 | 0.949 |
| Rosuvastatin | Clopidogrel | 0.685 | 0.203 | 0.698 |  | 0.808 | 0.309 | 1.000 |  | 1.000 | 0.755 | 2.752 |  | 0.061 | 0.000 | 0.646 |
| Rosuvastatin | Heparin sodium | 0.685 | 0.203 | 0.698 |  | 1.000 | 1.000 | 1.740 |  | 1.029 | 0.964 | 3.808 |  | 0.020 | -0.381 | 1.107 |
| Rosuvastatin | Warfarin | 0.685 | 0.203 | 0.698 |  | 0.907 | 0.652 | 1.285 |  | 1.000 | 0.816 | 5.702 |  | 0.029 | -0.115 | 1.794 |
| Rosuvastatin | Amlodipine | 0.685 | 0.203 | 0.698 |  | 0.797 | 0.467 | 0.994 |  | 1.000 | 0.362 | 1.643 |  | 0.064 | -0.143 | 0.395 |

**Supplementary Table S7. Continued.**

| Drug 1 | Drug 2 | Adjusted odds ratio | | | | | | | | | | |  | Synergistic interaction | | |
| --- | --- | --- | --- | --- | --- | --- | --- | --- | --- | --- | --- | --- | --- | --- | --- | --- |
|  |  | Drug 1 | | |  | Drug 2 | | |  | Product term (Drug 1*Drug 2) | | |  |  |  |  |
|  |  | Estimate | 95%BootCI | |  | Estimate | 95%BootCI | |  | Estimate | 95%BootCI | |  | RERI | 95%BootCI | |
|  |  |  | Lower | Upper |  |  | Lower | Upper |  |  | Lower | Upper |  |  | Lower | Upper |
| Rosuvastatin | Olmesartan | 0.685 | 0.203 | 0.698 |  | 1.000 | 0.339 | 1.000 |  | 1.000 | 0.330 | 2.036 |  | 0.000 | -0.150 | 0.521 |
| Rosuvastatin | Carvedilol | 0.685 | 0.203 | 0.698 |  | 1.000 | 0.765 | 1.536 |  | 1.000 | 0.710 | 2.594 |  | 0.000 | -0.301 | 0.554 |
| Rosuvastatin | Telmisartan | 0.685 | 0.203 | 0.698 |  | 0.603 | 0.225 | 0.872 |  | 1.000 | 0.317 | 1.655 |  | 0.125 | -0.024 | 0.562 |
| Rosuvastatin | Nifedipine | 0.685 | 0.203 | 0.698 |  | 1.000 | 0.710 | 1.340 |  | 0.898 | 0.155 | 1.107 |  | -0.070 | -0.484 | 0.173 |
| Rosuvastatin | Bisoprolol | 0.685 | 0.203 | 0.698 |  | 1.000 | 0.621 | 1.236 |  | 1.170 | 1.000 | 4.749 |  | 0.117 | -0.040 | 1.235 |
| Rosuvastatin | Furosemide | 0.685 | 0.203 | 0.698 |  | 1.204 | 1.079 | 2.334 |  | 1.000 | 0.613 | 2.817 |  | -0.064 | -0.893 | 0.755 |
| Rosuvastatin | Nicorandil | 0.685 | 0.203 | 0.698 |  | 1.000 | 0.861 | 2.193 |  | 0.990 | 0.095 | 1.000 |  | -0.007 | -1.214 | 0.014 |
| Rosuvastatin | Metoclopramide | 0.685 | 0.203 | 0.698 |  | 1.000 | 0.765 | 1.290 |  | 1.000 | 0.319 | 2.546 |  | 0.000 | -0.322 | 0.674 |
| Rosuvastatin | Esomeprazole | 0.685 | 0.203 | 0.698 |  | 0.671 | 0.373 | 0.954 |  | 1.000 | 0.799 | 3.762 |  | 0.104 | 0.016 | 0.799 |
| Rosuvastatin | Vonoprazan | 0.685 | 0.203 | 0.698 |  | 1.000 | 0.392 | 1.000 |  | 1.000 | 0.489 | 4.205 |  | 0.000 | -0.103 | 0.939 |
| Rosuvastatin | Rabeprazole | 0.685 | 0.203 | 0.698 |  | 1.000 | 0.538 | 1.000 |  | 1.000 | 0.589 | 2.531 |  | 0.000 | -0.076 | 0.588 |
| Rosuvastatin | Lansoprazole | 0.685 | 0.203 | 0.698 |  | 1.084 | 1.000 | 1.520 |  | 1.000 | 0.378 | 1.714 |  | -0.026 | -0.508 | 0.241 |
| Rosuvastatin | Rebamipide | 0.685 | 0.203 | 0.698 |  | 0.739 | 0.345 | 0.760 |  | 1.000 | 0.080 | 1.000 |  | 0.082 | -0.163 | 0.421 |
| Rosuvastatin | Magnesium oxide | 0.685 | 0.203 | 0.698 |  | 0.949 | 0.749 | 1.096 |  | 1.000 | 0.595 | 4.395 |  | 0.016 | -0.151 | 1.191 |
| Allopurinol | Heparin sodium | 1.000 | 0.686 | 1.330 |  | 1.000 | 1.000 | 1.740 |  | 1.000 | 0.507 | 2.292 |  | 0.000 | -0.697 | 1.627 |
| Allopurinol | Warfarin | 1.000 | 0.686 | 1.330 |  | 0.907 | 0.652 | 1.285 |  | 1.000 | 0.320 | 1.972 |  | 0.000 | -0.700 | 0.822 |
| Allopurinol | Amlodipine | 1.000 | 0.686 | 1.330 |  | 0.797 | 0.467 | 0.994 |  | 1.000 | 0.226 | 1.000 |  | 0.000 | -0.661 | 0.076 |
| Allopurinol | Olmesartan | 1.000 | 0.686 | 1.330 |  | 1.000 | 0.339 | 1.000 |  | 1.000 | 0.735 | 4.636 |  | 0.000 | -0.213 | 1.997 |
| Allopurinol | Carvedilol | 1.000 | 0.686 | 1.330 |  | 1.000 | 0.765 | 1.536 |  | 0.985 | 0.220 | 1.213 |  | -0.015 | -0.942 | 0.221 |
| Allopurinol | Candesartan | 1.000 | 0.686 | 1.330 |  | 0.854 | 0.400 | 1.000 |  | 0.720 | 0.176 | 2.029 |  | -0.239 | -0.666 | 0.750 |
| Allopurinol | Nifedipine | 1.000 | 0.686 | 1.330 |  | 1.000 | 0.710 | 1.340 |  | 0.943 | 0.125 | 1.004 |  | -0.057 | -0.965 | 0.038 |
| Allopurinol | Furosemide | 1.000 | 0.686 | 1.330 |  | 1.204 | 1.079 | 2.334 |  | 0.702 | 0.218 | 1.000 |  | -0.359 | -1.495 | 0.000 |
| Allopurinol | Famotidine | 1.000 | 0.686 | 1.330 |  | 1.643 | 1.332 | 2.071 |  | 1.000 | 0.272 | 1.573 |  | 0.000 | -1.226 | 0.798 |
| Allopurinol | Rabeprazole | 1.000 | 0.686 | 1.330 |  | 1.000 | 0.538 | 1.000 |  | 0.825 | 0.159 | 1.111 |  | -0.175 | -0.734 | 0.136 |
| Allopurinol | Lansoprazole | 1.000 | 0.686 | 1.330 |  | 1.084 | 1.000 | 1.520 |  | 1.000 | 0.597 | 2.432 |  | 0.000 | -0.483 | 1.572 |
| Allopurinol | Rebamipide | 1.000 | 0.686 | 1.330 |  | 0.739 | 0.345 | 0.760 |  | 1.000 | 0.957 | 5.482 |  | 0.000 | -0.095 | 2.120 |
| Allopurinol | Sennoside | 1.000 | 0.686 | 1.330 |  | 1.000 | 0.852 | 1.372 |  | 1.000 | 0.271 | 2.620 |  | 0.000 | -0.796 | 1.645 |
| Febuxostat | Heparin sodium | 0.626 | 0.226 | 0.986 |  | 1.000 | 1.000 | 1.740 |  | 1.000 | 0.779 | 5.298 |  | 0.000 | -0.391 | 2.006 |
| Febuxostat | Amlodipine | 0.626 | 0.226 | 0.986 |  | 0.797 | 0.467 | 0.994 |  | 1.000 | 0.264 | 1.226 |  | 0.076 | -0.372 | 0.351 |
| Febuxostat | Olmesartan | 0.626 | 0.226 | 0.986 |  | 1.000 | 0.339 | 1.000 |  | 1.000 | 0.134 | 2.105 |  | 0.000 | -0.336 | 0.562 |
| Febuxostat | Carvedilol | 0.626 | 0.226 | 0.986 |  | 1.000 | 0.765 | 1.536 |  | 1.000 | 0.541 | 3.788 |  | 0.000 | -0.351 | 1.379 |
| Febuxostat | Nifedipine | 0.626 | 0.226 | 0.986 |  | 1.000 | 0.710 | 1.340 |  | 1.000 | 0.178 | 1.787 |  | 0.000 | -0.542 | 0.383 |
| Febuxostat | Furosemide | 0.626 | 0.226 | 0.986 |  | 1.204 | 1.079 | 2.334 |  | 0.924 | 0.115 | 1.000 |  | -0.134 | -1.435 | -0.042 |
| Febuxostat | Esomeprazole | 0.626 | 0.226 | 0.986 |  | 0.671 | 0.373 | 0.954 |  | 1.000 | 0.294 | 2.424 |  | 0.123 | -0.222 | 0.602 |
| Methylergometrine | Teprenone | 0.368 | 0.080 | 0.364 |  | 0.605 | 0.480 | 1.000 |  | 1.000 | 0.455 | 1.000 |  | 0.250 | -0.014 | 0.417 |
| Methylergometrine | Magnesium oxide | 0.368 | 0.080 | 0.364 |  | 0.949 | 0.749 | 1.096 |  | 1.000 | 0.505 | 1.000 |  | 0.032 | -0.137 | 0.200 |
| Ritodrine | Teprenone | 1.000 | 0.334 | 1.000 |  | 0.605 | 0.480 | 1.000 |  | 1.000 | 0.259 | 1.647 |  | 0.000 | -0.516 | 0.415 |
| Ritodrine | Magnesium oxide | 1.000 | 0.334 | 1.000 |  | 0.949 | 0.749 | 1.096 |  | 1.000 | 0.210 | 1.046 |  | 0.000 | -0.720 | 0.104 |
| Tolvaptan | Furosemide | 1.000 | 1.000 | 8.068 |  | 1.204 | 1.079 | 2.334 |  | 1.000 | 0.102 | 1.000 |  | 0.000 | -6.176 | 1.421 |
| Alfacalcidol | Alendronate | 0.503 | 0.146 | 0.632 |  | 1.000 | 0.549 | 1.457 |  | 1.000 | 0.257 | 1.734 |  | 0.000 | -0.485 | 0.338 |
| Alfacalcidol | Heparin sodium | 0.503 | 0.146 | 0.632 |  | 1.000 | 1.000 | 1.740 |  | 1.000 | 0.586 | 3.385 |  | 0.000 | -0.530 | 0.688 |
| Alfacalcidol | Amlodipine | 0.503 | 0.146 | 0.632 |  | 0.797 | 0.467 | 0.994 |  | 1.000 | 0.433 | 1.912 |  | 0.101 | -0.058 | 0.433 |
| Alfacalcidol | Nifedipine | 0.503 | 0.146 | 0.632 |  | 1.000 | 0.710 | 1.340 |  | 1.000 | 0.203 | 3.309 |  | 0.000 | -0.375 | 0.688 |
| Alfacalcidol | Furosemide | 0.503 | 0.146 | 0.632 |  | 1.204 | 1.079 | 2.334 |  | 1.000 | 0.141 | 1.054 |  | -0.101 | -1.333 | -0.099 |
| Alfacalcidol | Esomeprazole | 0.503 | 0.146 | 0.632 |  | 0.671 | 0.373 | 0.954 |  | 1.000 | 0.077 | 1.000 |  | 0.163 | -0.240 | 0.432 |
| Alfacalcidol | Rabeprazole | 0.503 | 0.146 | 0.632 |  | 1.000 | 0.538 | 1.000 |  | 1.000 | 0.793 | 3.973 |  | 0.000 | 0.000 | 0.849 |

**Supplementary Table S7. Continued.**

| Drug 1 | Drug 2 | Adjusted odds ratio | | | | | | | | | | |  | Synergistic interaction | | |
| --- | --- | --- | --- | --- | --- | --- | --- | --- | --- | --- | --- | --- | --- | --- | --- | --- |
|  |  | Drug 1 | | |  | Drug 2 | | |  | Product term (Drug 1*Drug 2) | | |  |  |  |  |
|  |  | Estimate | 95%BootCI | |  | Estimate | 95%BootCI | |  | Estimate | 95%BootCI | |  | RERI | 95%BootCI | |
|  |  |  | Lower | Upper |  |  | Lower | Upper |  |  | Lower | Upper |  |  | Lower | Upper |
| Alfacalcidol | Lansoprazole | 0.503 | 0.146 | 0.632 |  | 1.084 | 1.000 | 1.520 |  | 1.000 | 0.318 | 1.776 |  | -0.042 | -0.551 | 0.146 |
| Alfacalcidol | Rebamipide | 0.503 | 0.146 | 0.632 |  | 0.739 | 0.345 | 0.760 |  | 1.000 | 0.302 | 2.394 |  | 0.129 | 0.055 | 0.574 |
| Alfacalcidol | Magnesium oxide | 0.503 | 0.146 | 0.632 |  | 0.949 | 0.749 | 1.096 |  | 0.855 | 0.071 | 1.000 |  | -0.044 | -0.470 | 0.126 |
| Alfacalcidol | Sennoside | 0.503 | 0.146 | 0.632 |  | 1.000 | 0.852 | 1.372 |  | 0.819 | 0.232 | 1.802 |  | -0.091 | -0.432 | 0.231 |
| Carbazochrome | Tranexamic acid | 1.000 | 0.377 | 1.000 |  | 0.446 | 0.263 | 0.796 |  | 1.000 | 0.463 | 1.008 |  | 0.000 | -0.288 | 0.327 |
| Carbazochrome | Heparin sodium | 1.000 | 0.377 | 1.000 |  | 1.000 | 1.000 | 1.740 |  | 1.000 | 1.000 | 6.298 |  | 0.000 | -0.140 | 4.346 |
| Carbazochrome | Amlodipine | 1.000 | 0.377 | 1.000 |  | 0.797 | 0.467 | 0.994 |  | 1.000 | 0.139 | 1.000 |  | 0.000 | -0.571 | 0.207 |
| Carbazochrome | L-carbocisteine | 1.000 | 0.377 | 1.000 |  | 1.000 | 0.595 | 1.000 |  | 1.000 | 0.412 | 1.651 |  | 0.000 | -0.389 | 0.423 |
| Carbazochrome | Metoclopramide | 1.000 | 0.377 | 1.000 |  | 1.000 | 0.765 | 1.290 |  | 1.000 | 0.192 | 1.000 |  | 0.000 | -0.635 | 0.088 |
| Carbazochrome | Famotidine | 1.000 | 0.377 | 1.000 |  | 1.643 | 1.332 | 2.071 |  | 1.000 | 0.786 | 3.025 |  | 0.000 | -0.523 | 2.055 |
| Carbazochrome | Rebamipide | 1.000 | 0.377 | 1.000 |  | 0.739 | 0.345 | 0.760 |  | 1.000 | 0.474 | 1.708 |  | 0.000 | -0.167 | 0.435 |
| Carbazochrome | Magnesium oxide | 1.000 | 0.377 | 1.000 |  | 0.949 | 0.749 | 1.096 |  | 1.000 | 0.165 | 1.273 |  | 0.000 | -0.692 | 0.188 |
| Carbazochrome | Haloperidol | 1.000 | 0.377 | 1.000 |  | 1.000 | 0.447 | 1.024 |  | 1.000 | 0.327 | 1.000 |  | 0.000 | -0.522 | 0.230 |
| Tranexamic acid | Heparin sodium | 0.446 | 0.263 | 0.796 |  | 1.000 | 1.000 | 1.740 |  | 0.905 | 0.197 | 1.000 |  | -0.042 | -0.979 | -0.036 |
| Tranexamic acid | Amlodipine | 0.446 | 0.263 | 0.796 |  | 0.797 | 0.467 | 0.994 |  | 1.000 | 0.409 | 2.924 |  | 0.113 | -0.125 | 0.734 |
| Tranexamic acid | L-carbocisteine | 0.446 | 0.263 | 0.796 |  | 1.000 | 0.595 | 1.000 |  | 1.000 | 0.430 | 1.299 |  | 0.000 | -0.276 | 0.259 |
| Tranexamic acid | Metoclopramide | 0.446 | 0.263 | 0.796 |  | 1.000 | 0.765 | 1.290 |  | 1.000 | 0.668 | 1.757 |  | 0.000 | -0.226 | 0.335 |
| Tranexamic acid | Teprenone | 0.446 | 0.263 | 0.796 |  | 0.605 | 0.480 | 1.000 |  | 1.000 | 0.476 | 2.510 |  | 0.219 | -0.105 | 0.630 |
| Tranexamic acid | Famotidine | 0.446 | 0.263 | 0.796 |  | 1.643 | 1.332 | 2.071 |  | 0.921 | 0.325 | 1.000 |  | -0.414 | -1.047 | -0.151 |
| Tranexamic acid | Ranitidine | 0.446 | 0.263 | 0.796 |  | 1.155 | 1.015 | 2.030 |  | 1.000 | 0.950 | 3.613 |  | -0.086 | -0.501 | 1.401 |
| Tranexamic acid | Rabeprazole | 0.446 | 0.263 | 0.796 |  | 1.000 | 0.538 | 1.000 |  | 1.000 | 0.169 | 1.980 |  | 0.000 | -0.339 | 0.467 |
| Tranexamic acid | Lansoprazole | 0.446 | 0.263 | 0.796 |  | 1.084 | 1.000 | 1.520 |  | 1.000 | 0.151 | 1.418 |  | -0.046 | -0.705 | 0.143 |
| Tranexamic acid | Rebamipide | 0.446 | 0.263 | 0.796 |  | 0.739 | 0.345 | 0.760 |  | 1.000 | 0.808 | 2.243 |  | 0.144 | 0.075 | 0.566 |
| Tranexamic acid | Magnesium oxide | 0.446 | 0.263 | 0.796 |  | 0.949 | 0.749 | 1.096 |  | 1.000 | 0.318 | 3.980 |  | 0.028 | -0.306 | 1.362 |
| Tranexamic acid | Haloperidol | 0.446 | 0.263 | 0.796 |  | 1.000 | 0.447 | 1.024 |  | 1.000 | 0.257 | 1.000 |  | 0.000 | -0.383 | 0.307 |
| Tranexamic acid | Midazolam | 0.446 | 0.263 | 0.796 |  | 1.000 | 0.814 | 1.575 |  | 1.000 | 0.161 | 1.000 |  | 0.000 | -0.776 | 0.071 |
| Edoxaban | Amlodipine | 1.000 | 0.457 | 1.126 |  | 0.797 | 0.467 | 0.994 |  | 1.000 | 0.100 | 1.000 |  | 0.000 | -0.707 | 0.174 |
| Edoxaban | Esomeprazole | 1.000 | 0.457 | 1.126 |  | 0.671 | 0.373 | 0.954 |  | 1.000 | 0.326 | 3.596 |  | 0.000 | -0.395 | 1.189 |
| Edoxaban | Misoprostol | 1.000 | 0.457 | 1.126 |  | 1.000 | 0.330 | 1.000 |  | 1.000 | 0.847 | 2.617 |  | 0.000 | -0.126 | 0.826 |
| Edoxaban | Rebamipide | 1.000 | 0.457 | 1.126 |  | 0.739 | 0.345 | 0.760 |  | 1.000 | 0.266 | 2.760 |  | 0.000 | -0.357 | 0.822 |
| Enoxaparin | Misoprostol | 1.148 | 1.000 | 6.144 |  | 1.000 | 0.330 | 1.000 |  | 1.000 | 0.646 | 5.558 |  | 0.000 | -2.343 | 4.880 |
| Clopidogrel | Heparin sodium | 0.808 | 0.309 | 1.000 |  | 1.000 | 1.000 | 1.740 |  | 1.000 | 0.477 | 1.656 |  | 0.000 | -0.684 | 0.452 |
| Clopidogrel | Amlodipine | 0.808 | 0.309 | 1.000 |  | 0.797 | 0.467 | 0.994 |  | 1.000 | 0.224 | 1.209 |  | 0.039 | -0.451 | 0.280 |
| Clopidogrel | Olmesartan | 0.808 | 0.309 | 1.000 |  | 1.000 | 0.339 | 1.000 |  | 1.000 | 0.239 | 1.705 |  | 0.000 | -0.379 | 0.459 |
| Clopidogrel | Carvedilol | 0.808 | 0.309 | 1.000 |  | 1.000 | 0.765 | 1.536 |  | 1.000 | 0.581 | 2.100 |  | 0.000 | -0.389 | 0.677 |
| Clopidogrel | Nifedipine | 0.808 | 0.309 | 1.000 |  | 1.000 | 0.710 | 1.340 |  | 0.631 | 0.081 | 1.000 |  | -0.298 | -0.831 | 0.036 |
| Clopidogrel | Bisoprolol | 0.808 | 0.309 | 1.000 |  | 1.000 | 0.621 | 1.236 |  | 1.000 | 0.395 | 1.880 |  | 0.000 | -0.453 | 0.535 |
| Clopidogrel | Furosemide | 0.808 | 0.309 | 1.000 |  | 1.204 | 1.079 | 2.334 |  | 0.936 | 0.396 | 1.647 |  | -0.102 | -1.024 | 0.394 |
| Clopidogrel | Nicorandil | 0.808 | 0.309 | 1.000 |  | 1.000 | 0.861 | 2.193 |  | 0.976 | 0.511 | 2.342 |  | -0.019 | -0.663 | 0.877 |
| Clopidogrel | Esomeprazole | 0.808 | 0.309 | 1.000 |  | 0.671 | 0.373 | 0.954 |  | 1.000 | 0.525 | 3.143 |  | 0.063 | -0.170 | 0.816 |
| Clopidogrel | Rabeprazole | 0.808 | 0.309 | 1.000 |  | 1.000 | 0.538 | 1.000 |  | 1.000 | 0.400 | 1.289 |  | 0.000 | -0.377 | 0.275 |
| Clopidogrel | Lansoprazole | 0.808 | 0.309 | 1.000 |  | 1.084 | 1.000 | 1.520 |  | 1.000 | 0.816 | 2.626 |  | -0.016 | -0.249 | 0.860 |
| Cilostazol | Heparin sodium | 0.815 | 0.379 | 1.000 |  | 1.000 | 1.000 | 1.740 |  | 1.000 | 0.186 | 1.014 |  | 0.000 | -1.071 | 0.000 |
| Cilostazol | Amlodipine | 0.815 | 0.379 | 1.000 |  | 0.797 | 0.467 | 0.994 |  | 0.941 | 0.122 | 1.000 |  | -0.001 | -0.633 | 0.215 |
| Cilostazol | Lansoprazole | 0.815 | 0.379 | 1.000 |  | 1.084 | 1.000 | 1.520 |  | 1.000 | 0.697 | 2.761 |  | -0.015 | -0.371 | 1.164 |
| Ticlopidine | Nicorandil | 1.000 | 0.742 | 2.285 |  | 1.000 | 0.861 | 2.193 |  | 1.000 | 0.232 | 1.449 |  | 0.000 | -1.558 | 0.599 |

**Supplementary Table S7. Continued.**

| Drug 1 | Drug 2 | Adjusted odds ratio | | | | | | | | | | |  | Synergistic interaction | | |
| --- | --- | --- | --- | --- | --- | --- | --- | --- | --- | --- | --- | --- | --- | --- | --- | --- |
|  |  | Drug 1 | | |  | Drug 2 | | |  | Product term (Drug 1*Drug 2) | | |  |  |  |  |
|  |  | Estimate | 95%BootCI | |  | Estimate | 95%BootCI | |  | Estimate | 95%BootCI | |  | RERI | 95%BootCI | |
|  |  |  | Lower | Upper |  |  | Lower | Upper |  |  | Lower | Upper |  |  | Lower | Upper |
| Fondaparinux | Misoprostol | 1.000 | 0.445 | 2.724 |  | 1.000 | 0.330 | 1.000 |  | 1.000 | 0.246 | 1.391 |  | 0.000 | -1.644 | 0.327 |
| Heparin sodium | Warfarin | 1.000 | 1.000 | 1.740 |  | 0.907 | 0.652 | 1.285 |  | 1.467 | 1.000 | 3.590 |  | 0.423 | -0.014 | 3.089 |
| Heparin sodium | Amlodipine | 1.000 | 1.000 | 1.740 |  | 0.797 | 0.467 | 0.994 |  | 1.000 | 0.656 | 1.593 |  | 0.000 | -0.475 | 0.411 |
| Heparin sodium | Olmesartan | 1.000 | 1.000 | 1.740 |  | 1.000 | 0.339 | 1.000 |  | 1.000 | 0.419 | 1.913 |  | 0.000 | -0.692 | 0.581 |
| Heparin sodium | Kallidinogenase | 1.000 | 1.000 | 1.740 |  | 0.547 | 0.141 | 0.964 |  | 1.000 | 0.772 | 4.896 |  | 0.000 | -0.479 | 1.234 |
| Heparin sodium | Carvedilol | 1.000 | 1.000 | 1.740 |  | 1.000 | 0.765 | 1.536 |  | 1.001 | 0.638 | 2.455 |  | 0.001 | -0.522 | 2.110 |
| Heparin sodium | Candesartan | 1.000 | 1.000 | 1.740 |  | 0.854 | 0.400 | 1.000 |  | 1.000 | 0.377 | 1.724 |  | 0.000 | -0.810 | 0.582 |
| Heparin sodium | Spironolactone | 1.000 | 1.000 | 1.740 |  | 1.000 | 0.452 | 1.310 |  | 1.286 | 1.000 | 5.306 |  | 0.286 | -0.095 | 5.128 |
| Heparin sodium | Telmisartan | 1.000 | 1.000 | 1.740 |  | 0.603 | 0.225 | 0.872 |  | 1.000 | 0.388 | 3.028 |  | 0.000 | -0.636 | 1.234 |
| Heparin sodium | Nifedipine | 1.000 | 1.000 | 1.740 |  | 1.000 | 0.710 | 1.340 |  | 1.000 | 0.513 | 1.729 |  | 0.000 | -0.701 | 0.873 |
| Heparin sodium | Bisoprolol | 1.000 | 1.000 | 1.740 |  | 1.000 | 0.621 | 1.236 |  | 1.000 | 0.672 | 2.237 |  | 0.000 | -0.437 | 1.512 |
| Heparin sodium | Furosemide | 1.000 | 1.000 | 1.740 |  | 1.204 | 1.079 | 2.334 |  | 1.993 | 1.000 | 2.834 |  | 1.195 | 0.208 | 4.441 |
| Heparin sodium | Nicorandil | 1.000 | 1.000 | 1.740 |  | 1.000 | 0.861 | 2.193 |  | 1.000 | 0.291 | 1.241 |  | 0.000 | -1.310 | 0.379 |
| Heparin sodium | Ambroxol | 1.000 | 1.000 | 1.740 |  | 1.000 | 0.702 | 1.258 |  | 1.000 | 0.258 | 1.227 |  | 0.000 | -1.071 | 0.283 |
| Heparin sodium | L-carbocisteine | 1.000 | 1.000 | 1.740 |  | 1.000 | 0.595 | 1.000 |  | 1.000 | 0.689 | 1.750 |  | 0.000 | -0.430 | 0.871 |
| Heparin sodium | Domperidone | 1.000 | 1.000 | 1.740 |  | 1.000 | 0.781 | 2.053 |  | 1.000 | 0.277 | 1.998 |  | 0.000 | -1.221 | 1.585 |
| Heparin sodium | Metoclopramide | 1.000 | 1.000 | 1.740 |  | 1.000 | 0.765 | 1.290 |  | 0.807 | 0.440 | 1.000 |  | -0.193 | -0.781 | 0.068 |
| Heparin sodium | Mosapride | 1.000 | 1.000 | 1.740 |  | 1.000 | 0.620 | 1.251 |  | 1.000 | 0.359 | 1.839 |  | 0.000 | -0.857 | 0.960 |
| Heparin sodium | Irsogladine | 1.000 | 1.000 | 1.740 |  | 0.912 | 0.342 | 1.000 |  | 1.000 | 0.402 | 1.938 |  | 0.000 | -0.721 | 0.618 |
| Heparin sodium | Esomeprazole | 1.000 | 1.000 | 1.740 |  | 0.671 | 0.373 | 0.954 |  | 1.000 | 0.421 | 1.505 |  | 0.000 | -0.696 | 0.268 |
| Heparin sodium | Omeprazole | 1.000 | 1.000 | 1.740 |  | 1.045 | 0.928 | 1.644 |  | 1.663 | 1.000 | 3.662 |  | 0.692 | 0.004 | 4.011 |
| Heparin sodium | Teprenone | 1.000 | 1.000 | 1.740 |  | 0.605 | 0.480 | 1.000 |  | 0.768 | 0.454 | 1.249 |  | -0.140 | -0.681 | 0.165 |
| Heparin sodium | Famotidine | 1.000 | 1.000 | 1.740 |  | 1.643 | 1.332 | 2.071 |  | 1.113 | 0.872 | 1.780 |  | 0.186 | -0.104 | 1.989 |
| Heparin sodium | Vonoprazan | 1.000 | 1.000 | 1.740 |  | 1.000 | 0.392 | 1.000 |  | 1.000 | 0.435 | 1.842 |  | 0.000 | -0.711 | 0.604 |
| Heparin sodium | Ranitidine | 1.000 | 1.000 | 1.740 |  | 1.155 | 1.015 | 2.030 |  | 1.000 | 0.294 | 1.000 |  | 0.000 | -1.367 | 0.200 |
| Heparin sodium | Rabeprazole | 1.000 | 1.000 | 1.740 |  | 1.000 | 0.538 | 1.000 |  | 1.402 | 1.000 | 3.100 |  | 0.402 | -0.072 | 2.041 |
| Heparin sodium | Lansoprazole | 1.000 | 1.000 | 1.740 |  | 1.084 | 1.000 | 1.520 |  | 1.000 | 0.829 | 1.840 |  | 0.000 | -0.196 | 1.303 |
| Heparin sodium | Rebamipide | 1.000 | 1.000 | 1.740 |  | 0.739 | 0.345 | 0.760 |  | 1.000 | 1.000 | 2.905 |  | 0.000 | -0.230 | 1.144 |
| Heparin sodium | Magnesium oxide | 1.000 | 1.000 | 1.740 |  | 0.949 | 0.749 | 1.096 |  | 1.000 | 0.636 | 1.537 |  | 0.000 | -0.488 | 0.650 |
| Heparin sodium | Senna | 1.000 | 1.000 | 1.740 |  | 0.956 | 0.531 | 1.007 |  | 1.000 | 0.346 | 2.106 |  | 0.000 | -0.812 | 1.110 |
| Heparin sodium | Sennoside | 1.000 | 1.000 | 1.740 |  | 1.000 | 0.852 | 1.372 |  | 1.000 | 0.502 | 1.348 |  | 0.000 | -0.724 | 0.476 |
| Heparin sodium | Picosulfate | 1.000 | 1.000 | 1.740 |  | 1.072 | 1.000 | 2.023 |  | 1.000 | 0.404 | 1.507 |  | 0.000 | -1.052 | 0.899 |
| Heparin sodium | Haloperidol | 1.000 | 1.000 | 1.740 |  | 1.000 | 0.447 | 1.024 |  | 0.975 | 0.371 | 1.102 |  | -0.025 | -0.792 | 0.094 |
| Heparin sodium | Etizolam | 1.000 | 1.000 | 1.740 |  | 0.805 | 0.270 | 0.865 |  | 1.000 | 0.573 | 3.009 |  | 0.000 | -0.497 | 1.066 |
| Heparin sodium | Zopiclone | 1.000 | 1.000 | 1.740 |  | 1.000 | 0.681 | 1.535 |  | 0.802 | 0.308 | 2.343 |  | -0.198 | -1.002 | 1.755 |
| Heparin sodium | Zolpidem | 1.000 | 1.000 | 1.740 |  | 1.000 | 0.767 | 1.233 |  | 0.935 | 0.326 | 1.081 |  | -0.065 | -0.973 | 0.104 |
| Heparin sodium | Brotizolam | 1.000 | 1.000 | 1.740 |  | 1.000 | 0.621 | 1.195 |  | 1.000 | 0.445 | 1.755 |  | 0.000 | -0.743 | 0.883 |
| Heparin sodium | Adenosine triphosphate | 1.000 | 1.000 | 1.740 |  | 0.679 | 0.272 | 1.000 |  | 1.000 | 0.285 | 1.513 |  | 0.000 | -0.844 | 0.429 |
| Heparin sodium | Edaravone | 1.000 | 1.000 | 1.740 |  | 1.000 | 0.616 | 2.595 |  | 1.000 | 0.146 | 1.066 |  | 0.000 | -2.127 | 0.193 |
| Heparin sodium | Fentanyl | 1.000 | 1.000 | 1.740 |  | 4.031 | 3.726 | 8.961 |  | 1.000 | 1.000 | 6.152 |  | 0.000 | 0.203 | 49.030 |
| Heparin sodium | Remifentanil | 1.000 | 1.000 | 1.740 |  | 1.000 | 0.473 | 1.000 |  | 0.917 | 0.187 | 1.218 |  | -0.083 | -1.119 | 0.201 |
| Heparin sodium | Midazolam | 1.000 | 1.000 | 1.740 |  | 1.000 | 0.814 | 1.575 |  | 1.000 | 0.294 | 1.000 |  | 0.000 | -1.102 | 0.102 |
| Heparin sodium | Daikenchuto | 1.000 | 1.000 | 1.740 |  | 1.000 | 0.925 | 1.936 |  | 1.000 | 0.418 | 1.742 |  | 0.000 | -0.964 | 1.229 |
| Warfarin | Amlodipine | 0.907 | 0.652 | 1.285 |  | 0.797 | 0.467 | 0.994 |  | 1.000 | 0.564 | 2.353 |  | 0.019 | -0.305 | 0.876 |
| Warfarin | Carvedilol | 0.907 | 0.652 | 1.285 |  | 1.000 | 0.765 | 1.536 |  | 1.000 | 0.373 | 1.575 |  | 0.000 | -0.669 | 0.563 |
| Warfarin | Candesartan | 0.907 | 0.652 | 1.285 |  | 0.854 | 0.400 | 1.000 |  | 1.000 | 0.309 | 2.122 |  | 0.014 | -0.553 | 0.684 |

**Supplementary Table S7. Continued.**

| Drug 1 | Drug 2 | Adjusted odds ratio | | | | | | | | | | |  | Synergistic interaction | | |
| --- | --- | --- | --- | --- | --- | --- | --- | --- | --- | --- | --- | --- | --- | --- | --- | --- |
|  |  | Drug 1 | | |  | Drug 2 | | |  | Product term (Drug 1*Drug 2) | | |  |  |  |  |
|  |  | Estimate | 95%BootCI | |  | Estimate | 95%BootCI | |  | Estimate | 95%BootCI | |  | RERI | 95%BootCI | |
|  |  |  | Lower | Upper |  |  | Lower | Upper |  |  | Lower | Upper |  |  | Lower | Upper |
| Warfarin | Spironolactone | 0.907 | 0.652 | 1.285 |  | 1.000 | 0.452 | 1.310 |  | 1.000 | 0.644 | 2.709 |  | 0.000 | -0.306 | 1.377 |
| Warfarin | Bisoprolol | 0.907 | 0.652 | 1.285 |  | 1.000 | 0.621 | 1.236 |  | 0.766 | 0.150 | 1.000 |  | -0.212 | -0.942 | 0.000 |
| Warfarin | Furosemide | 0.907 | 0.652 | 1.285 |  | 1.204 | 1.079 | 2.334 |  | 1.000 | 0.343 | 1.035 |  | -0.019 | -1.248 | 0.078 |
| Warfarin | Nicorandil | 0.907 | 0.652 | 1.285 |  | 1.000 | 0.861 | 2.193 |  | 0.816 | 0.146 | 1.064 |  | -0.167 | -1.424 | 0.092 |
| Warfarin | Digoxin | 0.907 | 0.652 | 1.285 |  | 1.000 | 0.512 | 1.702 |  | 1.000 | 0.212 | 1.266 |  | 0.000 | -1.028 | 0.234 |
| Warfarin | Famotidine | 0.907 | 0.652 | 1.285 |  | 1.643 | 1.332 | 2.071 |  | 0.764 | 0.282 | 1.327 |  | -0.412 | -1.251 | 0.426 |
| Warfarin | Rabeprazole | 0.907 | 0.652 | 1.285 |  | 1.000 | 0.538 | 1.000 |  | 1.000 | 0.472 | 1.782 |  | 0.000 | -0.453 | 0.534 |
| Warfarin | Lansoprazole | 0.907 | 0.652 | 1.285 |  | 1.084 | 1.000 | 1.520 |  | 1.000 | 0.416 | 1.509 |  | -0.008 | -0.753 | 0.524 |
| Warfarin | Magnesium oxide | 0.907 | 0.652 | 1.285 |  | 0.949 | 0.749 | 1.096 |  | 0.726 | 0.241 | 1.448 |  | -0.231 | -0.740 | 0.391 |
| Azilsartan | Amlodipine | 0.785 | 0.129 | 0.702 |  | 0.797 | 0.467 | 0.994 |  | 1.000 | 1.000 | 5.658 |  | 0.044 | 0.015 | 0.923 |
| Azelnidipine | Olmesartan | 0.599 | 0.232 | 1.000 |  | 1.000 | 0.339 | 1.000 |  | 0.469 | 0.135 | 1.247 |  | -0.318 | -0.491 | 0.400 |
| Atenolol | Amlodipine | 1.000 | 0.450 | 1.350 |  | 0.797 | 0.467 | 0.994 |  | 1.000 | 0.135 | 1.411 |  | 0.000 | -0.742 | 0.305 |
| Amlodipine | Irbesartan | 0.797 | 0.467 | 0.994 |  | 1.000 | 0.158 | 1.000 |  | 1.000 | 0.819 | 7.519 |  | 0.000 | -0.040 | 1.266 |
| Amlodipine | Enalapril | 0.797 | 0.467 | 0.994 |  | 0.812 | 0.317 | 1.000 |  | 1.000 | 0.137 | 1.241 |  | 0.038 | -0.559 | 0.290 |
| Amlodipine | Olmesartan | 0.797 | 0.467 | 0.994 |  | 1.000 | 0.339 | 1.000 |  | 1.000 | 0.637 | 1.837 |  | 0.000 | -0.141 | 0.461 |
| Amlodipine | Carvedilol | 0.797 | 0.467 | 0.994 |  | 1.000 | 0.765 | 1.536 |  | 0.682 | 0.247 | 1.000 |  | -0.253 | -0.757 | 0.022 |
| Amlodipine | Candesartan | 0.797 | 0.467 | 0.994 |  | 0.854 | 0.400 | 1.000 |  | 1.000 | 0.622 | 1.787 |  | 0.030 | -0.215 | 0.437 |
| Amlodipine | Spironolactone | 0.797 | 0.467 | 0.994 |  | 1.000 | 0.452 | 1.310 |  | 1.207 | 1.000 | 7.619 |  | 0.165 | 0.002 | 3.507 |
| Amlodipine | Telmisartan | 0.797 | 0.467 | 0.994 |  | 0.603 | 0.225 | 0.872 |  | 1.000 | 0.219 | 1.000 |  | 0.081 | -0.384 | 0.303 |
| Amlodipine | Trichlormethiazide | 0.797 | 0.467 | 0.994 |  | 0.419 | 0.122 | 0.872 |  | 1.000 | 0.050 | 1.000 |  | 0.118 | -0.462 | 0.364 |
| Amlodipine | Doxazosin | 0.797 | 0.467 | 0.994 |  | 1.000 | 0.505 | 1.310 |  | 0.954 | 0.211 | 1.000 |  | -0.036 | -0.735 | 0.158 |
| Amlodipine | Nifedipine | 0.797 | 0.467 | 0.994 |  | 1.000 | 0.710 | 1.340 |  | 1.000 | 0.688 | 2.938 |  | 0.000 | -0.221 | 1.252 |
| Amlodipine | Bisoprolol | 0.797 | 0.467 | 0.994 |  | 1.000 | 0.621 | 1.236 |  | 1.000 | 0.673 | 2.512 |  | 0.000 | -0.221 | 0.966 |
| Amlodipine | Furosemide | 0.797 | 0.467 | 0.994 |  | 1.204 | 1.079 | 2.334 |  | 0.887 | 0.361 | 1.000 |  | -0.150 | -1.197 | -0.014 |
| Amlodipine | Nicorandil | 0.797 | 0.467 | 0.994 |  | 1.000 | 0.861 | 2.193 |  | 0.604 | 0.099 | 1.000 |  | -0.315 | -1.433 | 0.000 |
| Amlodipine | Ambroxol | 0.797 | 0.467 | 0.994 |  | 1.000 | 0.702 | 1.258 |  | 1.000 | 0.559 | 1.873 |  | 0.000 | -0.337 | 0.586 |
| Amlodipine | L-carbocisteine | 0.797 | 0.467 | 0.994 |  | 1.000 | 0.595 | 1.000 |  | 1.212 | 0.868 | 2.413 |  | 0.169 | -0.063 | 0.848 |
| Amlodipine | Itopride | 0.797 | 0.467 | 0.994 |  | 2.076 | 1.030 | 3.338 |  | 1.292 | 0.598 | 2.780 |  | 0.264 | -1.060 | 2.161 |
| Amlodipine | Metoclopramide | 0.797 | 0.467 | 0.994 |  | 1.000 | 0.765 | 1.290 |  | 1.000 | 0.393 | 1.607 |  | 0.000 | -0.464 | 0.409 |
| Amlodipine | Mosapride | 0.797 | 0.467 | 0.994 |  | 1.000 | 0.620 | 1.251 |  | 1.000 | 0.683 | 2.938 |  | 0.000 | -0.214 | 1.261 |
| Amlodipine | Irsogladine | 0.797 | 0.467 | 0.994 |  | 0.912 | 0.342 | 1.000 |  | 1.000 | 0.062 | 1.000 |  | 0.018 | -0.647 | 0.203 |
| Amlodipine | Esomeprazole | 0.797 | 0.467 | 0.994 |  | 0.671 | 0.373 | 0.954 |  | 1.000 | 0.772 | 3.316 |  | 0.067 | -0.019 | 1.033 |
| Amlodipine | Omeprazole | 0.797 | 0.467 | 0.994 |  | 1.045 | 0.928 | 1.644 |  | 0.990 | 0.220 | 1.306 |  | -0.018 | -0.893 | 0.201 |
| Amlodipine | Teprenone | 0.797 | 0.467 | 0.994 |  | 0.605 | 0.480 | 1.000 |  | 1.000 | 0.623 | 2.634 |  | 0.080 | -0.160 | 0.879 |
| Amlodipine | Famotidine | 0.797 | 0.467 | 0.994 |  | 1.643 | 1.332 | 2.071 |  | 1.000 | 0.821 | 1.995 |  | -0.131 | -0.463 | 0.839 |
| Amlodipine | Vonoprazan | 0.797 | 0.467 | 0.994 |  | 1.000 | 0.392 | 1.000 |  | 1.000 | 0.913 | 3.900 |  | 0.000 | -0.011 | 1.305 |
| Amlodipine | Misoprostol | 0.797 | 0.467 | 0.994 |  | 1.000 | 0.330 | 1.000 |  | 1.000 | 0.239 | 1.726 |  | 0.000 | -0.465 | 0.443 |
| Amlodipine | Ranitidine | 0.797 | 0.467 | 0.994 |  | 1.155 | 1.015 | 2.030 |  | 1.108 | 0.768 | 3.669 |  | 0.068 | -0.396 | 2.524 |
| Amlodipine | Rabeprazole | 0.797 | 0.467 | 0.994 |  | 1.000 | 0.538 | 1.000 |  | 1.000 | 0.414 | 1.184 |  | 0.000 | -0.353 | 0.208 |
| Amlodipine | Lansoprazole | 0.797 | 0.467 | 0.994 |  | 1.084 | 1.000 | 1.520 |  | 1.000 | 0.625 | 1.532 |  | -0.017 | -0.409 | 0.345 |
| Amlodipine | Rebamipide | 0.797 | 0.467 | 0.994 |  | 0.739 | 0.345 | 0.760 |  | 1.000 | 0.350 | 1.154 |  | 0.053 | -0.197 | 0.295 |
| Amlodipine | Magnesium oxide | 0.797 | 0.467 | 0.994 |  | 0.949 | 0.749 | 1.096 |  | 1.000 | 0.697 | 1.771 |  | 0.010 | -0.212 | 0.495 |
| Amlodipine | Senna | 0.797 | 0.467 | 0.994 |  | 0.956 | 0.531 | 1.007 |  | 1.000 | 0.321 | 1.356 |  | 0.009 | -0.440 | 0.256 |
| Amlodipine | Sennoside | 0.797 | 0.467 | 0.994 |  | 1.000 | 0.852 | 1.372 |  | 1.000 | 0.405 | 1.435 |  | 0.000 | -0.523 | 0.300 |
| Amlodipine | Picosulfate | 0.797 | 0.467 | 0.994 |  | 1.072 | 1.000 | 2.023 |  | 1.000 | 0.818 | 3.444 |  | -0.015 | -0.413 | 2.096 |
| Amlodipine | Haloperidol | 0.797 | 0.467 | 0.994 |  | 1.000 | 0.447 | 1.024 |  | 1.000 | 0.784 | 3.779 |  | 0.000 | -0.121 | 1.670 |

**Supplementary Table S7. Continued.**

| Drug 1 | Drug 2 | Adjusted odds ratio | | | | | | | | | | |  | Synergistic interaction | | |
| --- | --- | --- | --- | --- | --- | --- | --- | --- | --- | --- | --- | --- | --- | --- | --- | --- |
|  |  | Drug 1 | | |  | Drug 2 | | |  | Product term (Drug 1*Drug 2) | | |  |  |  |  |
|  |  | Estimate | 95%BootCI | |  | Estimate | 95%BootCI | |  | Estimate | 95%BootCI | |  | RERI | 95%BootCI | |
|  |  |  | Lower | Upper |  |  | Lower | Upper |  |  | Lower | Upper |  |  | Lower | Upper |
| Amlodipine | Etizolam | 0.797 | 0.467 | 0.994 |  | 0.805 | 0.270 | 0.865 |  | 1.000 | 0.929 | 4.262 |  | 0.040 | 0.000 | 1.189 |
| Amlodipine | Zolpidem | 0.797 | 0.467 | 0.994 |  | 1.000 | 0.767 | 1.233 |  | 0.970 | 0.287 | 1.358 |  | -0.024 | -0.552 | 0.245 |
| Amlodipine | Brotizolam | 0.797 | 0.467 | 0.994 |  | 1.000 | 0.621 | 1.195 |  | 1.000 | 0.448 | 1.871 |  | 0.000 | -0.398 | 0.551 |
| Amlodipine | Fentanyl | 0.797 | 0.467 | 0.994 |  | 4.031 | 3.726 | 8.961 |  | 1.000 | 0.638 | 2.580 |  | -0.616 | -3.594 | 5.360 |
| Amlodipine | Daikenchuto | 0.797 | 0.467 | 0.994 |  | 1.000 | 0.925 | 1.936 |  | 1.000 | 0.706 | 2.694 |  | 0.000 | -0.434 | 1.251 |
| Enalapril | Carvedilol | 0.812 | 0.317 | 1.000 |  | 1.000 | 0.765 | 1.536 |  | 1.000 | 0.382 | 2.177 |  | 0.000 | -0.597 | 0.693 |
| Enalapril | Furosemide | 0.812 | 0.317 | 1.000 |  | 1.204 | 1.079 | 2.334 |  | 1.000 | 0.438 | 2.154 |  | -0.038 | -0.969 | 0.723 |
| Eplerenone | Carvedilol | 1.000 | 0.400 | 1.338 |  | 1.000 | 0.765 | 1.536 |  | 1.393 | 0.866 | 5.950 |  | 0.393 | -0.171 | 3.875 |
| Eplerenone | Furosemide | 1.000 | 0.400 | 1.338 |  | 1.204 | 1.079 | 2.334 |  | 1.000 | 0.329 | 1.806 |  | 0.000 | -1.141 | 0.899 |
| Eplerenone | Azosemide | 1.000 | 0.400 | 1.338 |  | 1.000 | 0.436 | 1.093 |  | 1.000 | 0.344 | 2.662 |  | 0.000 | -0.567 | 1.053 |
| Olmesartan | Carvedilol | 1.000 | 0.339 | 1.000 |  | 1.000 | 0.765 | 1.536 |  | 1.000 | 0.294 | 1.320 |  | 0.000 | -0.641 | 0.221 |
| Olmesartan | Cilnidipine | 1.000 | 0.339 | 1.000 |  | 1.000 | 0.354 | 1.000 |  | 1.000 | 0.309 | 1.881 |  | 0.000 | -0.397 | 0.485 |
| Olmesartan | Doxazosin | 1.000 | 0.339 | 1.000 |  | 1.000 | 0.505 | 1.310 |  | 1.000 | 0.179 | 1.863 |  | 0.000 | -0.629 | 0.515 |
| Olmesartan | Nifedipine | 1.000 | 0.339 | 1.000 |  | 1.000 | 0.710 | 1.340 |  | 1.000 | 0.790 | 2.653 |  | 0.000 | -0.182 | 0.897 |
| Olmesartan | Bisoprolol | 1.000 | 0.339 | 1.000 |  | 1.000 | 0.621 | 1.236 |  | 1.000 | 0.442 | 2.511 |  | 0.000 | -0.383 | 0.848 |
| Olmesartan | Furosemide | 1.000 | 0.339 | 1.000 |  | 1.204 | 1.079 | 2.334 |  | 1.000 | 0.721 | 2.898 |  | 0.000 | -0.625 | 1.584 |
| Olmesartan | Benidipine | 1.000 | 0.339 | 1.000 |  | 0.714 | 0.353 | 1.000 |  | 1.000 | 0.266 | 1.376 |  | 0.000 | -0.372 | 0.365 |
| Olmesartan | Nicorandil | 1.000 | 0.339 | 1.000 |  | 1.000 | 0.861 | 2.193 |  | 1.000 | 0.298 | 3.480 |  | 0.000 | -0.769 | 2.124 |
| Olmesartan | Ambroxol | 1.000 | 0.339 | 1.000 |  | 1.000 | 0.702 | 1.258 |  | 1.000 | 0.472 | 2.545 |  | 0.000 | -0.345 | 0.921 |
| Olmesartan | L-carbocisteine | 1.000 | 0.339 | 1.000 |  | 1.000 | 0.595 | 1.000 |  | 1.000 | 0.361 | 1.903 |  | 0.000 | -0.379 | 0.542 |
| Olmesartan | Itopride | 1.000 | 0.339 | 1.000 |  | 2.076 | 1.030 | 3.338 |  | 1.452 | 0.880 | 4.672 |  | 0.938 | -0.860 | 4.054 |
| Olmesartan | Esomeprazole | 1.000 | 0.339 | 1.000 |  | 0.671 | 0.373 | 0.954 |  | 1.000 | 0.189 | 3.871 |  | 0.000 | -0.343 | 1.203 |
| Olmesartan | Famotidine | 1.000 | 0.339 | 1.000 |  | 1.643 | 1.332 | 2.071 |  | 1.000 | 0.757 | 2.702 |  | 0.000 | -0.559 | 1.339 |
| Olmesartan | Rabeprazole | 1.000 | 0.339 | 1.000 |  | 1.000 | 0.538 | 1.000 |  | 1.000 | 0.658 | 2.622 |  | 0.000 | -0.154 | 0.806 |
| Olmesartan | Lansoprazole | 1.000 | 0.339 | 1.000 |  | 1.084 | 1.000 | 1.520 |  | 1.000 | 0.396 | 1.526 |  | 0.000 | -0.622 | 0.294 |
| Olmesartan | Rebamipide | 1.000 | 0.339 | 1.000 |  | 0.739 | 0.345 | 0.760 |  | 1.000 | 0.194 | 1.495 |  | 0.000 | -0.246 | 0.409 |
| Olmesartan | Magnesium oxide | 1.000 | 0.339 | 1.000 |  | 0.949 | 0.749 | 1.096 |  | 1.000 | 0.437 | 2.626 |  | 0.000 | -0.417 | 0.866 |
| Kallidinogenase | Rabeprazole | 0.547 | 0.141 | 0.964 |  | 1.000 | 0.538 | 1.000 |  | 1.000 | 0.506 | 1.816 |  | 0.000 | -0.127 | 0.400 |
| Kallidinogenase | Adenosine triphosphate | 0.547 | 0.141 | 0.964 |  | 0.679 | 0.272 | 1.000 |  | 1.000 | 0.276 | 1.020 |  | 0.146 | -0.384 | 0.456 |
| Carvedilol | Candesartan | 1.000 | 0.765 | 1.536 |  | 0.854 | 0.400 | 1.000 |  | 1.000 | 0.140 | 1.134 |  | 0.000 | -0.908 | 0.124 |
| Carvedilol | Spironolactone | 1.000 | 0.765 | 1.536 |  | 1.000 | 0.452 | 1.310 |  | 1.000 | 0.383 | 1.491 |  | 0.000 | -0.759 | 0.394 |
| Carvedilol | Nifedipine | 1.000 | 0.765 | 1.536 |  | 1.000 | 0.710 | 1.340 |  | 0.871 | 0.276 | 1.111 |  | -0.129 | -0.884 | 0.120 |
| Carvedilol | Furosemide | 1.000 | 0.765 | 1.536 |  | 1.204 | 1.079 | 2.334 |  | 1.000 | 0.745 | 2.033 |  | 0.000 | -0.425 | 1.844 |
| Carvedilol | Nicorandil | 1.000 | 0.765 | 1.536 |  | 1.000 | 0.861 | 2.193 |  | 1.000 | 0.364 | 1.490 |  | 0.000 | -0.972 | 0.654 |
| Carvedilol | Azosemide | 1.000 | 0.765 | 1.536 |  | 1.000 | 0.436 | 1.093 |  | 1.000 | 0.627 | 3.339 |  | 0.000 | -0.384 | 1.978 |
| Carvedilol | Esomeprazole | 1.000 | 0.765 | 1.536 |  | 0.671 | 0.373 | 0.954 |  | 1.000 | 0.415 | 2.371 |  | 0.000 | -0.453 | 0.840 |
| Carvedilol | Famotidine | 1.000 | 0.765 | 1.536 |  | 1.643 | 1.332 | 2.071 |  | 1.063 | 0.714 | 2.993 |  | 0.103 | -0.489 | 3.385 |
| Carvedilol | Vonoprazan | 1.000 | 0.765 | 1.536 |  | 1.000 | 0.392 | 1.000 |  | 1.000 | 0.197 | 1.397 |  | 0.000 | -0.798 | 0.250 |
| Carvedilol | Rabeprazole | 1.000 | 0.765 | 1.536 |  | 1.000 | 0.538 | 1.000 |  | 1.000 | 0.582 | 2.083 |  | 0.000 | -0.424 | 0.896 |
| Carvedilol | Lansoprazole | 1.000 | 0.765 | 1.536 |  | 1.084 | 1.000 | 1.520 |  | 1.000 | 0.426 | 1.611 |  | 0.000 | -0.739 | 0.758 |
| Carvedilol | Rebamipide | 1.000 | 0.765 | 1.536 |  | 0.739 | 0.345 | 0.760 |  | 1.000 | 0.484 | 3.637 |  | 0.000 | -0.372 | 1.260 |
| Carvedilol | Magnesium oxide | 1.000 | 0.765 | 1.536 |  | 0.949 | 0.749 | 1.096 |  | 1.000 | 0.312 | 1.948 |  | 0.000 | -0.729 | 1.031 |
| Carvedilol | Sennoside | 1.000 | 0.765 | 1.536 |  | 1.000 | 0.852 | 1.372 |  | 0.699 | 0.058 | 1.000 |  | -0.301 | -1.343 | 0.000 |
| Candesartan | Nifedipine | 0.854 | 0.400 | 1.000 |  | 1.000 | 0.710 | 1.340 |  | 1.000 | 0.766 | 4.315 |  | 0.000 | -0.195 | 1.994 |
| Candesartan | Furosemide | 0.854 | 0.400 | 1.000 |  | 1.204 | 1.079 | 2.334 |  | 1.000 | 0.525 | 2.337 |  | -0.030 | -0.851 | 1.337 |
| Candesartan | Famotidine | 0.854 | 0.400 | 1.000 |  | 1.643 | 1.332 | 2.071 |  | 1.022 | 1.000 | 4.994 |  | -0.063 | -0.215 | 4.122 |

**Supplementary Table S7. Continued.**

| Drug 1 | Drug 2 | Adjusted odds ratio | | | | | | | | | | |  | Synergistic interaction | | |
| --- | --- | --- | --- | --- | --- | --- | --- | --- | --- | --- | --- | --- | --- | --- | --- | --- |
|  |  | Drug 1 | | |  | Drug 2 | | |  | Product term (Drug 1*Drug 2) | | |  |  |  |  |
|  |  | Estimate | 95%BootCI | |  | Estimate | 95%BootCI | |  | Estimate | 95%BootCI | |  | RERI | 95%BootCI | |
|  |  |  | Lower | Upper |  |  | Lower | Upper |  |  | Lower | Upper |  |  | Lower | Upper |
| Candesartan | Rabeprazole | 0.854 | 0.400 | 1.000 |  | 1.000 | 0.538 | 1.000 |  | 1.000 | 0.872 | 5.675 |  | 0.000 | -0.009 | 2.302 |
| Candesartan | Lansoprazole | 0.854 | 0.400 | 1.000 |  | 1.084 | 1.000 | 1.520 |  | 1.000 | 0.546 | 2.956 |  | -0.012 | -0.442 | 1.315 |
| Candesartan | Rebamipide | 0.854 | 0.400 | 1.000 |  | 0.739 | 0.345 | 0.760 |  | 1.000 | 0.098 | 1.011 |  | 0.038 | -0.412 | 0.297 |
| Candesartan | Magnesium oxide | 0.854 | 0.400 | 1.000 |  | 0.949 | 0.749 | 1.096 |  | 1.000 | 0.197 | 1.004 |  | 0.007 | -0.674 | 0.076 |
| Cilnidipine | Ambroxol | 1.000 | 0.354 | 1.000 |  | 1.000 | 0.702 | 1.258 |  | 1.000 | 0.796 | 5.850 |  | 0.000 | -0.147 | 3.066 |
| Cilnidipine | L-carbocisteine | 1.000 | 0.354 | 1.000 |  | 1.000 | 0.595 | 1.000 |  | 1.137 | 0.891 | 6.072 |  | 0.137 | -0.049 | 2.736 |
| Cilnidipine | Azulene sulfonate | 1.000 | 0.354 | 1.000 |  | 0.890 | 0.498 | 1.051 |  | 1.000 | 0.288 | 2.101 |  | 0.000 | -0.522 | 0.675 |
| Cilnidipine | Famotidine | 1.000 | 0.354 | 1.000 |  | 1.643 | 1.332 | 2.071 |  | 1.000 | 0.407 | 2.266 |  | 0.000 | -0.966 | 0.915 |
| Cilnidipine | Lansoprazole | 1.000 | 0.354 | 1.000 |  | 1.084 | 1.000 | 1.520 |  | 1.349 | 0.568 | 3.857 |  | 0.379 | -0.443 | 2.134 |
| Spironolactone | Bisoprolol | 1.000 | 0.452 | 1.310 |  | 1.000 | 0.621 | 1.236 |  | 1.000 | 0.208 | 1.000 |  | 0.000 | -0.832 | 0.063 |
| Spironolactone | Furosemide | 1.000 | 0.452 | 1.310 |  | 1.204 | 1.079 | 2.334 |  | 0.868 | 0.360 | 1.000 |  | -0.159 | -1.225 | 0.002 |
| Spironolactone | Nicorandil | 1.000 | 0.452 | 1.310 |  | 1.000 | 0.861 | 2.193 |  | 0.957 | 0.211 | 1.586 |  | -0.043 | -1.082 | 0.560 |
| Spironolactone | Azosemide | 1.000 | 0.452 | 1.310 |  | 1.000 | 0.436 | 1.093 |  | 0.943 | 0.279 | 1.634 |  | -0.057 | -0.699 | 0.429 |
| Spironolactone | Rabeprazole | 1.000 | 0.452 | 1.310 |  | 1.000 | 0.538 | 1.000 |  | 1.000 | 0.640 | 2.884 |  | 0.000 | -0.267 | 1.298 |
| Spironolactone | Lansoprazole | 1.000 | 0.452 | 1.310 |  | 1.084 | 1.000 | 1.520 |  | 1.000 | 0.309 | 1.420 |  | 0.000 | -0.831 | 0.405 |
| Telmisartan | Nifedipine | 0.603 | 0.225 | 0.872 |  | 1.000 | 0.710 | 1.340 |  | 1.000 | 0.673 | 4.532 |  | 0.000 | -0.227 | 1.305 |
| Telmisartan | Furosemide | 0.603 | 0.225 | 0.872 |  | 1.204 | 1.079 | 2.334 |  | 1.000 | 0.333 | 1.888 |  | -0.081 | -1.113 | 0.253 |
| Telmisartan | Rabeprazole | 0.603 | 0.225 | 0.872 |  | 1.000 | 0.538 | 1.000 |  | 1.000 | 0.419 | 2.243 |  | 0.000 | -0.237 | 0.531 |
| Telmisartan | Lansoprazole | 0.603 | 0.225 | 0.872 |  | 1.084 | 1.000 | 1.520 |  | 1.000 | 0.229 | 1.364 |  | -0.033 | -0.731 | 0.113 |
| Telmisartan | Rebamipide | 0.603 | 0.225 | 0.872 |  | 0.739 | 0.345 | 0.760 |  | 1.000 | 0.164 | 3.800 |  | 0.103 | -0.084 | 0.912 |
| Telmisartan | Magnesium oxide | 0.603 | 0.225 | 0.872 |  | 0.949 | 0.749 | 1.096 |  | 1.000 | 0.218 | 2.808 |  | 0.020 | -0.430 | 0.689 |
| Doxazosin | Nifedipine | 1.000 | 0.505 | 1.310 |  | 1.000 | 0.710 | 1.340 |  | 1.000 | 0.320 | 1.846 |  | 0.000 | -0.695 | 0.757 |
| Doxazosin | Furosemide | 1.000 | 0.505 | 1.310 |  | 1.204 | 1.079 | 2.334 |  | 0.410 | 0.042 | 0.944 |  | -0.711 | -1.992 | -0.227 |
| Nifedipine | Bisoprolol | 1.000 | 0.710 | 1.340 |  | 1.000 | 0.621 | 1.236 |  | 1.000 | 0.445 | 2.144 |  | 0.000 | -0.565 | 1.006 |
| Nifedipine | Furosemide | 1.000 | 0.710 | 1.340 |  | 1.204 | 1.079 | 2.334 |  | 0.552 | 0.279 | 1.000 |  | -0.540 | -1.432 | 0.000 |
| Nifedipine | Nicorandil | 1.000 | 0.710 | 1.340 |  | 1.000 | 0.861 | 2.193 |  | 0.925 | 0.426 | 2.424 |  | -0.075 | -0.752 | 1.801 |
| Nifedipine | Metoclopramide | 1.000 | 0.710 | 1.340 |  | 1.000 | 0.765 | 1.290 |  | 1.000 | 0.154 | 1.000 |  | 0.000 | -0.970 | 0.022 |
| Nifedipine | Esomeprazole | 1.000 | 0.710 | 1.340 |  | 0.671 | 0.373 | 0.954 |  | 0.778 | 0.128 | 1.000 |  | -0.149 | -0.737 | 0.078 |
| Nifedipine | Teprenone | 1.000 | 0.710 | 1.340 |  | 0.605 | 0.480 | 1.000 |  | 1.000 | 0.121 | 1.000 |  | 0.000 | -0.771 | 0.066 |
| Nifedipine | Famotidine | 1.000 | 0.710 | 1.340 |  | 1.643 | 1.332 | 2.071 |  | 1.030 | 0.821 | 2.436 |  | 0.049 | -0.304 | 2.334 |
| Nifedipine | Rabeprazole | 1.000 | 0.710 | 1.340 |  | 1.000 | 0.538 | 1.000 |  | 0.804 | 0.218 | 1.075 |  | -0.196 | -0.741 | 0.101 |
| Nifedipine | Lansoprazole | 1.000 | 0.710 | 1.340 |  | 1.084 | 1.000 | 1.520 |  | 1.000 | 0.585 | 2.149 |  | 0.000 | -0.498 | 1.279 |
| Nifedipine | Rebamipide | 1.000 | 0.710 | 1.340 |  | 0.739 | 0.345 | 0.760 |  | 1.000 | 0.370 | 2.173 |  | 0.000 | -0.367 | 0.594 |
| Nifedipine | Magnesium oxide | 1.000 | 0.710 | 1.340 |  | 0.949 | 0.749 | 1.096 |  | 0.966 | 0.171 | 1.000 |  | -0.032 | -0.873 | 0.004 |
| Nifedipine | Senna | 1.000 | 0.710 | 1.340 |  | 0.956 | 0.531 | 1.007 |  | 1.000 | 0.826 | 4.482 |  | 0.000 | -0.138 | 2.783 |
| Nifedipine | Sennoside | 1.000 | 0.710 | 1.340 |  | 1.000 | 0.852 | 1.372 |  | 1.000 | 0.444 | 2.378 |  | 0.000 | -0.600 | 1.366 |
| Nifedipine | Zolpidem | 1.000 | 0.710 | 1.340 |  | 1.000 | 0.767 | 1.233 |  | 1.000 | 0.253 | 2.439 |  | 0.000 | -0.790 | 1.416 |
| Bisoprolol | Furosemide | 1.000 | 0.621 | 1.236 |  | 1.204 | 1.079 | 2.334 |  | 1.002 | 0.681 | 2.279 |  | 0.002 | -0.522 | 1.848 |
| Bisoprolol | Azosemide | 1.000 | 0.621 | 1.236 |  | 1.000 | 0.436 | 1.093 |  | 1.019 | 0.595 | 3.387 |  | 0.019 | -0.340 | 1.592 |
| Bisoprolol | Esomeprazole | 1.000 | 0.621 | 1.236 |  | 0.671 | 0.373 | 0.954 |  | 1.000 | 0.284 | 1.968 |  | 0.000 | -0.461 | 0.555 |
| Bisoprolol | Vonoprazan | 1.000 | 0.621 | 1.236 |  | 1.000 | 0.392 | 1.000 |  | 1.000 | 0.213 | 1.522 |  | 0.000 | -0.672 | 0.354 |
| Bisoprolol | Rabeprazole | 1.000 | 0.621 | 1.236 |  | 1.000 | 0.538 | 1.000 |  | 1.000 | 0.417 | 1.568 |  | 0.000 | -0.491 | 0.430 |
| Bisoprolol | Lansoprazole | 1.000 | 0.621 | 1.236 |  | 1.084 | 1.000 | 1.520 |  | 1.000 | 0.554 | 2.150 |  | 0.000 | -0.518 | 1.232 |
| Bisoprolol | Rebamipide | 1.000 | 0.621 | 1.236 |  | 0.739 | 0.345 | 0.760 |  | 1.000 | 0.467 | 5.009 |  | 0.000 | -0.285 | 1.935 |
| Bisoprolol | Magnesium oxide | 1.000 | 0.621 | 1.236 |  | 0.949 | 0.749 | 1.096 |  | 1.000 | 0.285 | 2.883 |  | 0.000 | -0.661 | 1.803 |
| Furosemide | Benidipine | 1.204 | 1.079 | 2.334 |  | 0.714 | 0.353 | 1.000 |  | 1.000 | 0.374 | 2.699 |  | -0.058 | -1.026 | 1.284 |

**Supplementary Table S7. Continued.**

| Drug 1 | Drug 2 | Adjusted odds ratio | | | | | | | | | | |  | Synergistic interaction | | |
| --- | --- | --- | --- | --- | --- | --- | --- | --- | --- | --- | --- | --- | --- | --- | --- | --- |
|  |  | Drug 1 | | |  | Drug 2 | | |  | Product term (Drug 1*Drug 2) | | |  |  |  |  |
|  |  | Estimate | 95%BootCI | |  | Estimate | 95%BootCI | |  | Estimate | 95%BootCI | |  | RERI | 95%BootCI | |
|  |  |  | Lower | Upper |  |  | Lower | Upper |  |  | Lower | Upper |  |  | Lower | Upper |
| Furosemide | Nicorandil | 1.204 | 1.079 | 2.334 |  | 1.000 | 0.861 | 2.193 |  | 1.000 | 0.447 | 1.814 |  | 0.000 | -1.036 | 1.846 |
| Furosemide | Isosorbide mononitrate | 1.204 | 1.079 | 2.334 |  | 1.000 | 0.491 | 1.492 |  | 1.000 | 0.254 | 1.839 |  | 0.000 | -1.407 | 1.213 |
| Furosemide | Digoxin | 1.204 | 1.079 | 2.334 |  | 1.000 | 0.512 | 1.702 |  | 1.000 | 0.340 | 1.884 |  | 0.000 | -1.254 | 1.205 |
| Furosemide | Ambroxol | 1.204 | 1.079 | 2.334 |  | 1.000 | 0.702 | 1.258 |  | 0.573 | 0.112 | 1.000 |  | -0.514 | -1.796 | 0.000 |
| Furosemide | L-carbocisteine | 1.204 | 1.079 | 2.334 |  | 1.000 | 0.595 | 1.000 |  | 0.785 | 0.249 | 1.536 |  | -0.259 | -1.315 | 0.665 |
| Furosemide | Metoclopramide | 1.204 | 1.079 | 2.334 |  | 1.000 | 0.765 | 1.290 |  | 1.000 | 0.737 | 3.362 |  | 0.000 | -0.444 | 3.731 |
| Furosemide | Mosapride | 1.204 | 1.079 | 2.334 |  | 1.000 | 0.620 | 1.251 |  | 1.380 | 1.000 | 5.723 |  | 0.457 | -0.066 | 7.487 |
| Furosemide | Esomeprazole | 1.204 | 1.079 | 2.334 |  | 0.671 | 0.373 | 0.954 |  | 1.000 | 0.776 | 3.781 |  | -0.067 | -0.600 | 2.405 |
| Furosemide | Omeprazole | 1.204 | 1.079 | 2.334 |  | 1.045 | 0.928 | 1.644 |  | 1.000 | 0.505 | 2.062 |  | 0.009 | -0.914 | 2.067 |
| Furosemide | Teprenone | 1.204 | 1.079 | 2.334 |  | 0.605 | 0.480 | 1.000 |  | 1.000 | 0.370 | 2.093 |  | -0.081 | -1.048 | 1.075 |
| Furosemide | Famotidine | 1.204 | 1.079 | 2.334 |  | 1.643 | 1.332 | 2.071 |  | 1.000 | 0.354 | 1.008 |  | 0.131 | -1.453 | 0.526 |
| Furosemide | Vonoprazan | 1.204 | 1.079 | 2.334 |  | 1.000 | 0.392 | 1.000 |  | 1.000 | 0.369 | 2.379 |  | 0.000 | -1.093 | 1.329 |
| Furosemide | Rabeprazole | 1.204 | 1.079 | 2.334 |  | 1.000 | 0.538 | 1.000 |  | 1.000 | 0.527 | 1.551 |  | 0.000 | -0.916 | 0.557 |
| Furosemide | Lansoprazole | 1.204 | 1.079 | 2.334 |  | 1.084 | 1.000 | 1.520 |  | 1.000 | 0.647 | 1.618 |  | 0.017 | -0.609 | 1.158 |
| Furosemide | Rebamipide | 1.204 | 1.079 | 2.334 |  | 0.739 | 0.345 | 0.760 |  | 1.000 | 0.415 | 1.770 |  | -0.053 | -0.975 | 0.394 |
| Furosemide | Magnesium oxide | 1.204 | 1.079 | 2.334 |  | 0.949 | 0.749 | 1.096 |  | 0.967 | 0.286 | 1.000 |  | -0.048 | -1.342 | 0.000 |
| Furosemide | Senna | 1.204 | 1.079 | 2.334 |  | 0.956 | 0.531 | 1.007 |  | 0.869 | 0.220 | 1.322 |  | -0.160 | -1.369 | 0.232 |
| Furosemide | Sennoside | 1.204 | 1.079 | 2.334 |  | 1.000 | 0.852 | 1.372 |  | 0.902 | 0.280 | 1.169 |  | -0.118 | -1.404 | 0.330 |
| Furosemide | Picosulfate | 1.204 | 1.079 | 2.334 |  | 1.072 | 1.000 | 2.023 |  | 1.000 | 0.443 | 2.351 |  | 0.015 | -1.037 | 3.265 |
| Furosemide | Haloperidol | 1.204 | 1.079 | 2.334 |  | 1.000 | 0.447 | 1.024 |  | 1.000 | 0.420 | 2.853 |  | 0.000 | -0.952 | 2.525 |
| Furosemide | Etizolam | 1.204 | 1.079 | 2.334 |  | 0.805 | 0.270 | 0.865 |  | 1.000 | 0.494 | 3.083 |  | -0.040 | -0.921 | 1.442 |
| Furosemide | Zolpidem | 1.204 | 1.079 | 2.334 |  | 1.000 | 0.767 | 1.233 |  | 1.000 | 0.629 | 2.473 |  | 0.000 | -0.642 | 2.499 |
| Furosemide | Brotizolam | 1.204 | 1.079 | 2.334 |  | 1.000 | 0.621 | 1.195 |  | 0.599 | 0.177 | 1.100 |  | -0.483 | -1.517 | 0.155 |
| Furosemide | Polystyrene sulfonate | 1.204 | 1.079 | 2.334 |  | 1.000 | 0.612 | 1.479 |  | 1.000 | 0.187 | 1.706 |  | 0.000 | -1.513 | 1.120 |
| Benidipine | Nicorandil | 0.714 | 0.353 | 1.000 |  | 1.000 | 0.861 | 2.193 |  | 1.000 | 0.093 | 1.000 |  | 0.000 | -1.457 | 0.004 |
| Benidipine | Rabeprazole | 0.714 | 0.353 | 1.000 |  | 1.000 | 0.538 | 1.000 |  | 1.000 | 0.617 | 3.342 |  | 0.000 | -0.139 | 1.177 |
| Nicorandil | Isosorbide mononitrate | 1.000 | 0.861 | 2.193 |  | 1.000 | 0.491 | 1.492 |  | 1.000 | 0.234 | 1.075 |  | 0.000 | -1.311 | 0.090 |
| Nicorandil | Omeprazole | 1.000 | 0.861 | 2.193 |  | 1.045 | 0.928 | 1.644 |  | 1.000 | 0.899 | 3.670 |  | 0.000 | -0.118 | 3.765 |
| Nicorandil | Famotidine | 1.000 | 0.861 | 2.193 |  | 1.643 | 1.332 | 2.071 |  | 1.000 | 0.491 | 2.706 |  | 0.000 | -0.903 | 3.106 |
| Nicorandil | Rabeprazole | 1.000 | 0.861 | 2.193 |  | 1.000 | 0.538 | 1.000 |  | 1.000 | 0.304 | 1.553 |  | 0.000 | -1.011 | 0.427 |
| Nicorandil | Lansoprazole | 1.000 | 0.861 | 2.193 |  | 1.084 | 1.000 | 1.520 |  | 1.000 | 0.566 | 2.916 |  | 0.000 | -0.611 | 2.750 |
| Nicorandil | Magnesium oxide | 1.000 | 0.861 | 2.193 |  | 0.949 | 0.749 | 1.096 |  | 1.000 | 0.343 | 1.986 |  | 0.000 | -0.874 | 1.145 |
| Noradrenalin | Fentanyl | 1.000 | 0.277 | 2.006 |  | 4.031 | 3.726 | 8.961 |  | 0.545 | 0.064 | 1.000 |  | -1.834 | -7.895 | -1.598 |
| Salmeterol | Fluticasone | 1.000 | 0.154 | 1.000 |  | 1.000 | 0.565 | 2.040 |  | 1.000 | 1.000 | 2.000 |  | 0.000 | -0.769 | 0.388 |
| Theophylline | L-carbocisteine | 0.904 | 0.340 | 1.070 |  | 1.000 | 0.595 | 1.000 |  | 1.000 | 0.238 | 1.872 |  | 0.000 | -0.639 | 0.472 |
| Budesonide | Formoterol | 0.423 | 0.079 | 1.000 |  | 0.742 | 0.033 | 0.947 |  | 1.000 | 0.510 | 1.000 |  | 0.149 | -0.038 | 0.715 |
| Ambroxol | L-carbocisteine | 1.000 | 0.702 | 1.258 |  | 1.000 | 0.595 | 1.000 |  | 1.000 | 0.747 | 1.725 |  | 0.000 | -0.233 | 0.533 |
| Ambroxol | Itopride | 1.000 | 0.702 | 1.258 |  | 2.076 | 1.030 | 3.338 |  | 1.000 | 0.735 | 2.382 |  | 0.000 | -0.668 | 2.145 |
| Ambroxol | Mosapride | 1.000 | 0.702 | 1.258 |  | 1.000 | 0.620 | 1.251 |  | 1.000 | 0.255 | 1.039 |  | 0.000 | -0.781 | 0.062 |
| Ambroxol | Azulene sulfonate | 1.000 | 0.702 | 1.258 |  | 0.890 | 0.498 | 1.051 |  | 1.000 | 0.372 | 1.624 |  | 0.000 | -0.583 | 0.497 |
| Ambroxol | Teprenone | 1.000 | 0.702 | 1.258 |  | 0.605 | 0.480 | 1.000 |  | 1.000 | 0.423 | 2.570 |  | 0.000 | -0.446 | 1.062 |
| Ambroxol | Famotidine | 1.000 | 0.702 | 1.258 |  | 1.643 | 1.332 | 2.071 |  | 1.000 | 0.500 | 1.294 |  | 0.000 | -0.891 | 0.433 |
| Ambroxol | Polaprezinc | 1.000 | 0.702 | 1.258 |  | 1.000 | 0.668 | 1.512 |  | 1.000 | 0.223 | 2.388 |  | 0.000 | -0.816 | 1.461 |
| Ambroxol | Lansoprazole | 1.000 | 0.702 | 1.258 |  | 1.084 | 1.000 | 1.520 |  | 1.000 | 0.840 | 2.697 |  | 0.000 | -0.202 | 1.915 |
| Ambroxol | Rebamipide | 1.000 | 0.702 | 1.258 |  | 0.739 | 0.345 | 0.760 |  | 1.000 | 0.832 | 3.131 |  | 0.000 | -0.147 | 1.083 |
| Ambroxol | Magnesium oxide | 1.000 | 0.702 | 1.258 |  | 0.949 | 0.749 | 1.096 |  | 0.815 | 0.293 | 1.231 |  | -0.176 | -0.713 | 0.231 |

**Supplementary Table S7. Continued.**

| Drug 1 | Drug 2 | Adjusted odds ratio | | | | | | | | | | |  | Synergistic interaction | | |
| --- | --- | --- | --- | --- | --- | --- | --- | --- | --- | --- | --- | --- | --- | --- | --- | --- |
|  |  | Drug 1 | | |  | Drug 2 | | |  | Product term (Drug 1*Drug 2) | | |  |  |  |  |
|  |  | Estimate | 95%BootCI | |  | Estimate | 95%BootCI | |  | Estimate | 95%BootCI | |  | RERI | 95%BootCI | |
|  |  |  | Lower | Upper |  |  | Lower | Upper |  |  | Lower | Upper |  |  | Lower | Upper |
| Ambroxol | Senna | 1.000 | 0.702 | 1.258 |  | 0.956 | 0.531 | 1.007 |  | 1.000 | 0.452 | 2.365 |  | 0.000 | -0.477 | 1.023 |
| Ambroxol | Phenytoin | 1.000 | 0.702 | 1.258 |  | 2.847 | 1.685 | 4.093 |  | 1.000 | 0.404 | 1.957 |  | 0.000 | -1.821 | 2.319 |
| Ambroxol | Fentanyl | 1.000 | 0.702 | 1.258 |  | 4.031 | 3.726 | 8.961 |  | 1.000 | 0.392 | 1.573 |  | 0.000 | -4.246 | 3.235 |
| Ambroxol | Daikenchuto | 1.000 | 0.702 | 1.258 |  | 1.000 | 0.925 | 1.936 |  | 1.396 | 0.744 | 2.813 |  | 0.396 | -0.344 | 2.307 |
| L-carbocisteine | Dextromethorphan | 1.000 | 0.595 | 1.000 |  | 1.000 | 0.549 | 1.173 |  | 1.000 | 0.483 | 1.919 |  | 0.000 | -0.476 | 0.580 |
| L-carbocisteine | Itopride | 1.000 | 0.595 | 1.000 |  | 2.076 | 1.030 | 3.338 |  | 1.289 | 0.983 | 3.318 |  | 0.600 | -0.378 | 3.617 |
| L-carbocisteine | Metoclopramide | 1.000 | 0.595 | 1.000 |  | 1.000 | 0.765 | 1.290 |  | 1.000 | 0.562 | 2.825 |  | 0.000 | -0.368 | 1.586 |
| L-carbocisteine | Mosapride | 1.000 | 0.595 | 1.000 |  | 1.000 | 0.620 | 1.251 |  | 1.000 | 1.000 | 4.626 |  | 0.000 | -0.001 | 2.787 |
| L-carbocisteine | Azulene sulfonate | 1.000 | 0.595 | 1.000 |  | 0.890 | 0.498 | 1.051 |  | 1.000 | 0.179 | 1.000 |  | 0.000 | -0.729 | 0.045 |
| L-carbocisteine | Esomeprazole | 1.000 | 0.595 | 1.000 |  | 0.671 | 0.373 | 0.954 |  | 1.000 | 0.400 | 2.844 |  | 0.000 | -0.299 | 1.003 |
| L-carbocisteine | Teprenone | 1.000 | 0.595 | 1.000 |  | 0.605 | 0.480 | 1.000 |  | 1.000 | 0.145 | 1.000 |  | 0.000 | -0.654 | 0.136 |
| L-carbocisteine | Famotidine | 1.000 | 0.595 | 1.000 |  | 1.643 | 1.332 | 2.071 |  | 0.843 | 0.414 | 1.072 |  | -0.258 | -1.020 | 0.015 |
| L-carbocisteine | Vonoprazan | 1.000 | 0.595 | 1.000 |  | 1.000 | 0.392 | 1.000 |  | 1.000 | 0.411 | 2.387 |  | 0.000 | -0.418 | 0.802 |
| L-carbocisteine | Polaprezinc | 1.000 | 0.595 | 1.000 |  | 1.000 | 0.668 | 1.512 |  | 1.000 | 0.539 | 3.789 |  | 0.000 | -0.410 | 2.330 |
| L-carbocisteine | Rabeprazole | 1.000 | 0.595 | 1.000 |  | 1.000 | 0.538 | 1.000 |  | 1.000 | 0.258 | 1.679 |  | 0.000 | -0.558 | 0.465 |
| L-carbocisteine | Lansoprazole | 1.000 | 0.595 | 1.000 |  | 1.084 | 1.000 | 1.520 |  | 1.000 | 0.470 | 1.457 |  | 0.000 | -0.602 | 0.430 |
| L-carbocisteine | Rebamipide | 1.000 | 0.595 | 1.000 |  | 0.739 | 0.345 | 0.760 |  | 1.000 | 0.723 | 1.705 |  | 0.000 | -0.111 | 0.386 |
| L-carbocisteine | Magnesium oxide | 1.000 | 0.595 | 1.000 |  | 0.949 | 0.749 | 1.096 |  | 0.775 | 0.392 | 1.399 |  | -0.213 | -0.533 | 0.310 |
| L-carbocisteine | Senna | 1.000 | 0.595 | 1.000 |  | 0.956 | 0.531 | 1.007 |  | 1.000 | 0.626 | 3.062 |  | 0.000 | -0.268 | 1.542 |
| L-carbocisteine | Sennoside | 1.000 | 0.595 | 1.000 |  | 1.000 | 0.852 | 1.372 |  | 1.000 | 0.372 | 2.789 |  | 0.000 | -0.604 | 1.575 |
| L-carbocisteine | Haloperidol | 1.000 | 0.595 | 1.000 |  | 1.000 | 0.447 | 1.024 |  | 0.993 | 0.180 | 1.037 |  | -0.007 | -0.721 | 0.141 |
| L-carbocisteine | Fentanyl | 1.000 | 0.595 | 1.000 |  | 4.031 | 3.726 | 8.961 |  | 1.293 | 0.709 | 2.644 |  | 1.182 | -2.505 | 7.538 |
| L-carbocisteine | Midazolam | 1.000 | 0.595 | 1.000 |  | 1.000 | 0.814 | 1.575 |  | 1.000 | 0.830 | 5.435 |  | 0.000 | -0.173 | 3.932 |
| L-carbocisteine | Daikenchuto | 1.000 | 0.595 | 1.000 |  | 1.000 | 0.925 | 1.936 |  | 1.146 | 0.572 | 2.413 |  | 0.146 | -0.563 | 1.498 |
| Itopride | Azulene sulfonate | 2.076 | 1.030 | 3.338 |  | 0.890 | 0.498 | 1.051 |  | 1.000 | 0.557 | 2.500 |  | -0.119 | -1.042 | 2.091 |
| Itopride | Famotidine | 2.076 | 1.030 | 3.338 |  | 1.643 | 1.332 | 2.071 |  | 1.000 | 0.496 | 1.369 |  | 0.692 | -1.117 | 1.611 |
| Itopride | Polaprezinc | 2.076 | 1.030 | 3.338 |  | 1.000 | 0.668 | 1.512 |  | 1.843 | 0.960 | 6.470 |  | 1.750 | -0.183 | 9.964 |
| Itopride | Lansoprazole | 2.076 | 1.030 | 3.338 |  | 1.084 | 1.000 | 1.520 |  | 1.000 | 0.294 | 1.182 |  | 0.090 | -1.897 | 0.591 |
| Itopride | Magnesium oxide | 2.076 | 1.030 | 3.338 |  | 0.949 | 0.749 | 1.096 |  | 1.000 | 0.643 | 2.555 |  | -0.054 | -0.881 | 2.432 |
| Itopride | Senna | 2.076 | 1.030 | 3.338 |  | 0.956 | 0.531 | 1.007 |  | 1.000 | 0.369 | 1.807 |  | -0.047 | -1.518 | 1.134 |
| Itopride | Phenytoin | 2.076 | 1.030 | 3.338 |  | 2.847 | 1.685 | 4.093 |  | 1.000 | 0.495 | 2.302 |  | 1.988 | -1.019 | 8.554 |
| Itopride | Fentanyl | 2.076 | 1.030 | 3.338 |  | 4.031 | 3.726 | 8.961 |  | 1.115 | 0.397 | 1.783 |  | 4.225 | -2.505 | 15.472 |
| Itopride | Daikenchuto | 2.076 | 1.030 | 3.338 |  | 1.000 | 0.925 | 1.936 |  | 1.000 | 0.451 | 2.082 |  | 0.000 | -1.279 | 2.552 |
| Diastase | Pancreatin | 1.000 | 0.779 | 1.000 |  | 1.000 | 1.000 | 2.076 |  | 1.000 | 1.000 | 4.227 |  | 0.000 | 0.000 | 3.337 |
| Diastase | Magnesium oxide | 1.000 | 0.779 | 1.000 |  | 0.949 | 0.749 | 1.096 |  | 1.000 | 0.175 | 1.000 |  | 0.000 | -0.821 | 0.008 |
| Domperidone | Metoclopramide | 1.000 | 0.781 | 2.053 |  | 1.000 | 0.765 | 1.290 |  | 0.891 | 0.204 | 1.260 |  | -0.109 | -1.344 | 0.289 |
| Domperidone | Magnesium oxide | 1.000 | 0.781 | 2.053 |  | 0.949 | 0.749 | 1.096 |  | 1.000 | 0.310 | 2.660 |  | 0.000 | -0.927 | 1.803 |
| Metoclopramide | Esomeprazole | 1.000 | 0.765 | 1.290 |  | 0.671 | 0.373 | 0.954 |  | 1.000 | 0.504 | 2.827 |  | 0.000 | -0.329 | 1.099 |
| Metoclopramide | Teprenone | 1.000 | 0.765 | 1.290 |  | 0.605 | 0.480 | 1.000 |  | 1.000 | 0.663 | 3.244 |  | 0.000 | -0.231 | 1.568 |
| Metoclopramide | Famotidine | 1.000 | 0.765 | 1.290 |  | 1.643 | 1.332 | 2.071 |  | 1.000 | 0.527 | 1.377 |  | 0.000 | -0.842 | 0.612 |
| Metoclopramide | Ranitidine | 1.000 | 0.765 | 1.290 |  | 1.155 | 1.015 | 2.030 |  | 0.937 | 0.196 | 1.284 |  | -0.072 | -1.370 | 0.385 |
| Metoclopramide | Rabeprazole | 1.000 | 0.765 | 1.290 |  | 1.000 | 0.538 | 1.000 |  | 1.000 | 0.965 | 3.783 |  | 0.000 | -0.068 | 2.151 |
| Metoclopramide | Lansoprazole | 1.000 | 0.765 | 1.290 |  | 1.084 | 1.000 | 1.520 |  | 1.000 | 0.383 | 1.420 |  | 0.000 | -0.751 | 0.501 |
| Metoclopramide | Rebamipide | 1.000 | 0.765 | 1.290 |  | 0.739 | 0.345 | 0.760 |  | 1.000 | 0.282 | 1.000 |  | 0.000 | -0.489 | 0.070 |
| Metoclopramide | Magnesium oxide | 1.000 | 0.765 | 1.290 |  | 0.949 | 0.749 | 1.096 |  | 1.000 | 0.702 | 2.036 |  | 0.000 | -0.291 | 0.975 |
| Metoclopramide | Sennoside | 1.000 | 0.765 | 1.290 |  | 1.000 | 0.852 | 1.372 |  | 1.000 | 0.351 | 1.619 |  | 0.000 | -0.718 | 0.646 |

**Supplementary Table S7. Continued.**

| Drug 1 | Drug 2 | Adjusted odds ratio | | | | | | | | | | |  | Synergistic interaction | | |
| --- | --- | --- | --- | --- | --- | --- | --- | --- | --- | --- | --- | --- | --- | --- | --- | --- |
|  |  | Drug 1 | | |  | Drug 2 | | |  | Product term (Drug 1*Drug 2) | | |  |  |  |  |
|  |  | Estimate | 95%BootCI | |  | Estimate | 95%BootCI | |  | Estimate | 95%BootCI | |  | RERI | 95%BootCI | |
|  |  |  | Lower | Upper |  |  | Lower | Upper |  |  | Lower | Upper |  |  | Lower | Upper |
| Metoclopramide | Picosulfate | 1.000 | 0.765 | 1.290 |  | 1.072 | 1.000 | 2.023 |  | 1.187 | 0.467 | 2.607 |  | 0.200 | -0.725 | 2.073 |
| Metoclopramide | Haloperidol | 1.000 | 0.765 | 1.290 |  | 1.000 | 0.447 | 1.024 |  | 1.000 | 0.668 | 1.777 |  | 0.000 | -0.304 | 0.636 |
| Metoclopramide | Zolpidem | 1.000 | 0.765 | 1.290 |  | 1.000 | 0.767 | 1.233 |  | 1.000 | 0.607 | 2.860 |  | 0.000 | -0.387 | 1.857 |
| Metoclopramide | Adenosine triphosphate | 1.000 | 0.765 | 1.290 |  | 0.679 | 0.272 | 1.000 |  | 1.000 | 0.212 | 1.699 |  | 0.000 | -0.708 | 0.462 |
| Metoclopramide | Fentanyl | 1.000 | 0.765 | 1.290 |  | 4.031 | 3.726 | 8.961 |  | 1.000 | 0.501 | 2.656 |  | 0.000 | -3.217 | 10.504 |
| Metoclopramide | Buprenorphine | 1.000 | 0.765 | 1.290 |  | 1.000 | 0.998 | 7.643 |  | 0.925 | 0.020 | 1.000 |  | -0.075 | -6.379 | 0.001 |
| Metoclopramide | Pentazocine | 1.000 | 0.765 | 1.290 |  | 1.000 | 0.564 | 1.922 |  | 1.000 | 0.702 | 2.889 |  | 0.000 | -0.315 | 1.777 |
| Metoclopramide | Remifentanil | 1.000 | 0.765 | 1.290 |  | 1.000 | 0.473 | 1.000 |  | 0.495 | 0.145 | 1.000 |  | -0.505 | -0.821 | 0.008 |
| Metoclopramide | Daikenchuto | 1.000 | 0.765 | 1.290 |  | 1.000 | 0.925 | 1.936 |  | 1.090 | 0.660 | 3.730 |  | 0.090 | -0.431 | 3.285 |
| Mosapride | Azulene sulfonate | 1.000 | 0.620 | 1.251 |  | 0.890 | 0.498 | 1.051 |  | 1.000 | 0.854 | 3.829 |  | 0.000 | -0.129 | 2.139 |
| Mosapride | Famotidine | 1.000 | 0.620 | 1.251 |  | 1.643 | 1.332 | 2.071 |  | 1.000 | 0.628 | 1.637 |  | 0.000 | -0.665 | 0.835 |
| Mosapride | Rabeprazole | 1.000 | 0.620 | 1.251 |  | 1.000 | 0.538 | 1.000 |  | 1.000 | 0.761 | 2.923 |  | 0.000 | -0.176 | 1.336 |
| Mosapride | Lansoprazole | 1.000 | 0.620 | 1.251 |  | 1.084 | 1.000 | 1.520 |  | 1.000 | 0.285 | 1.000 |  | 0.000 | -0.927 | 0.006 |
| Mosapride | Rebamipide | 1.000 | 0.620 | 1.251 |  | 0.739 | 0.345 | 0.760 |  | 1.000 | 0.671 | 3.061 |  | 0.000 | -0.208 | 1.030 |
| Mosapride | Magnesium oxide | 1.000 | 0.620 | 1.251 |  | 0.949 | 0.749 | 1.096 |  | 1.000 | 0.307 | 1.000 |  | 0.000 | -0.733 | 0.017 |
| Mosapride | Senna | 1.000 | 0.620 | 1.251 |  | 0.956 | 0.531 | 1.007 |  | 1.000 | 0.350 | 1.630 |  | 0.000 | -0.586 | 0.456 |
| Mosapride | Sennoside | 1.000 | 0.620 | 1.251 |  | 1.000 | 0.852 | 1.372 |  | 1.000 | 0.788 | 3.539 |  | 0.000 | -0.227 | 2.390 |
| Mosapride | Fentanyl | 1.000 | 0.620 | 1.251 |  | 4.031 | 3.726 | 8.961 |  | 1.000 | 0.425 | 1.948 |  | 0.000 | -3.888 | 5.307 |
| Mosapride | Daikenchuto | 1.000 | 0.620 | 1.251 |  | 1.000 | 0.925 | 1.936 |  | 1.000 | 0.799 | 2.459 |  | 0.000 | -0.273 | 1.585 |
| Azulene sulfonate | Famotidine | 0.890 | 0.498 | 1.051 |  | 1.643 | 1.332 | 2.071 |  | 1.000 | 0.614 | 1.987 |  | -0.071 | -0.672 | 1.145 |
| Azulene sulfonate | Lansoprazole | 0.890 | 0.498 | 1.051 |  | 1.084 | 1.000 | 1.520 |  | 1.183 | 0.859 | 3.430 |  | 0.167 | -0.187 | 2.264 |
| Azulene sulfonate | Senna | 0.890 | 0.498 | 1.051 |  | 0.956 | 0.531 | 1.007 |  | 1.337 | 0.808 | 4.422 |  | 0.291 | -0.125 | 2.301 |
| Azulene sulfonate | Fentanyl | 0.890 | 0.498 | 1.051 |  | 4.031 | 3.726 | 8.961 |  | 1.000 | 0.637 | 2.813 |  | -0.334 | -3.059 | 8.705 |
| Azulene sulfonate | Daikenchuto | 0.890 | 0.498 | 1.051 |  | 1.000 | 0.925 | 1.936 |  | 1.000 | 0.521 | 3.071 |  | 0.000 | -0.570 | 2.290 |
| Esomeprazole | Rebamipide | 0.671 | 0.373 | 0.954 |  | 0.739 | 0.345 | 0.760 |  | 1.000 | 0.497 | 2.185 |  | 0.086 | -0.049 | 0.552 |
| Esomeprazole | Magnesium oxide | 0.671 | 0.373 | 0.954 |  | 0.949 | 0.749 | 1.096 |  | 1.000 | 0.166 | 1.615 |  | 0.017 | -0.545 | 0.354 |
| Omeprazole | Teprenone | 1.045 | 0.928 | 1.644 |  | 0.605 | 0.480 | 1.000 |  | 1.000 | 0.248 | 1.692 |  | -0.018 | -0.805 | 0.531 |
| Omeprazole | Rebamipide | 1.045 | 0.928 | 1.644 |  | 0.739 | 0.345 | 0.760 |  | 1.000 | 0.334 | 1.538 |  | -0.012 | -0.662 | 0.246 |
| Omeprazole | Magnesium oxide | 1.045 | 0.928 | 1.644 |  | 0.949 | 0.749 | 1.096 |  | 1.000 | 0.303 | 1.540 |  | -0.002 | -0.894 | 0.555 |
| Teprenone | Famotidine | 0.605 | 0.480 | 1.000 |  | 1.643 | 1.332 | 2.071 |  | 1.000 | 0.887 | 2.539 |  | -0.254 | -0.404 | 1.549 |
| Teprenone | Ranitidine | 0.605 | 0.480 | 1.000 |  | 1.155 | 1.015 | 2.030 |  | 1.000 | 0.585 | 2.445 |  | -0.061 | -0.622 | 1.382 |
| Teprenone | Rabeprazole | 0.605 | 0.480 | 1.000 |  | 1.000 | 0.538 | 1.000 |  | 1.028 | 0.938 | 3.406 |  | 0.017 | 0.000 | 1.234 |
| Teprenone | Lansoprazole | 0.605 | 0.480 | 1.000 |  | 1.084 | 1.000 | 1.520 |  | 1.000 | 0.935 | 3.668 |  | -0.033 | -0.171 | 2.099 |
| Teprenone | Rebamipide | 0.605 | 0.480 | 1.000 |  | 0.739 | 0.345 | 0.760 |  | 1.000 | 0.389 | 2.799 |  | 0.103 | -0.135 | 0.759 |
| Teprenone | Magnesium oxide | 0.605 | 0.480 | 1.000 |  | 0.949 | 0.749 | 1.096 |  | 1.000 | 0.520 | 1.410 |  | 0.020 | -0.334 | 0.293 |
| Teprenone | Sennoside | 0.605 | 0.480 | 1.000 |  | 1.000 | 0.852 | 1.372 |  | 1.000 | 0.307 | 1.863 |  | 0.000 | -0.622 | 0.623 |
| Teprenone | Picosulfate | 0.605 | 0.480 | 1.000 |  | 1.072 | 1.000 | 2.023 |  | 1.000 | 0.227 | 1.586 |  | -0.028 | -1.046 | 0.451 |
| Teprenone | Etizolam | 0.605 | 0.480 | 1.000 |  | 0.805 | 0.270 | 0.865 |  | 1.000 | 0.106 | 1.502 |  | 0.077 | -0.359 | 0.359 |
| Teprenone | Zolpidem | 0.605 | 0.480 | 1.000 |  | 1.000 | 0.767 | 1.233 |  | 1.000 | 0.452 | 2.422 |  | 0.000 | -0.422 | 1.009 |
| Teprenone | Midazolam | 0.605 | 0.480 | 1.000 |  | 1.000 | 0.814 | 1.575 |  | 0.895 | 0.086 | 1.000 |  | -0.063 | -0.938 | 0.012 |
| Teprenone | Daikenchuto | 0.605 | 0.480 | 1.000 |  | 1.000 | 0.925 | 1.936 |  | 1.000 | 0.022 | 1.000 |  | 0.000 | -1.454 | 0.000 |
| Famotidine | Polaprezinc | 1.643 | 1.332 | 2.071 |  | 1.000 | 0.668 | 1.512 |  | 0.499 | 0.189 | 1.020 |  | -0.824 | -1.543 | 0.119 |
| Famotidine | Ranitidine | 1.643 | 1.332 | 2.071 |  | 1.155 | 1.015 | 2.030 |  | 1.000 | 0.416 | 1.793 |  | 0.100 | -1.145 | 2.157 |
| Famotidine | Rabeprazole | 1.643 | 1.332 | 2.071 |  | 1.000 | 0.538 | 1.000 |  | 1.068 | 0.803 | 3.558 |  | 0.111 | -0.434 | 3.142 |
| Famotidine | Lansoprazole | 1.643 | 1.332 | 2.071 |  | 1.084 | 1.000 | 1.520 |  | 1.000 | 0.887 | 2.566 |  | 0.054 | -0.100 | 3.219 |
| Famotidine | Rebamipide | 1.643 | 1.332 | 2.071 |  | 0.739 | 0.345 | 0.760 |  | 0.987 | 0.541 | 1.426 |  | -0.184 | -0.839 | 0.030 |

**Supplementary Table S7. Continued.**

| Drug 1 | Drug 2 | Adjusted odds ratio | | | | | | | | | | |  | Synergistic interaction | | |
| --- | --- | --- | --- | --- | --- | --- | --- | --- | --- | --- | --- | --- | --- | --- | --- | --- |
|  |  | Drug 1 | | |  | Drug 2 | | |  | Product term (Drug 1*Drug 2) | | |  |  |  |  |
|  |  | Estimate | 95%BootCI | |  | Estimate | 95%BootCI | |  | Estimate | 95%BootCI | |  | RERI | 95%BootCI | |
|  |  |  | Lower | Upper |  |  | Lower | Upper |  |  | Lower | Upper |  |  | Lower | Upper |
| Famotidine | Magnesium oxide | 1.643 | 1.332 | 2.071 |  | 0.949 | 0.749 | 1.096 |  | 1.000 | 0.792 | 1.632 |  | -0.032 | -0.359 | 0.904 |
| Famotidine | Senna | 1.643 | 1.332 | 2.071 |  | 0.956 | 0.531 | 1.007 |  | 1.000 | 0.427 | 1.547 |  | -0.028 | -0.966 | 0.591 |
| Famotidine | Sennoside | 1.643 | 1.332 | 2.071 |  | 1.000 | 0.852 | 1.372 |  | 1.000 | 0.717 | 2.077 |  | 0.000 | -0.460 | 1.939 |
| Famotidine | Picosulfate | 1.643 | 1.332 | 2.071 |  | 1.072 | 1.000 | 2.023 |  | 1.000 | 0.521 | 2.162 |  | 0.046 | -0.907 | 2.773 |
| Famotidine | Haloperidol | 1.643 | 1.332 | 2.071 |  | 1.000 | 0.447 | 1.024 |  | 1.000 | 0.619 | 2.237 |  | 0.000 | -0.665 | 1.655 |
| Famotidine | Etizolam | 1.643 | 1.332 | 2.071 |  | 0.805 | 0.270 | 0.865 |  | 1.000 | 0.460 | 2.531 |  | -0.126 | -0.870 | 0.852 |
| Famotidine | Zolpidem | 1.643 | 1.332 | 2.071 |  | 1.000 | 0.767 | 1.233 |  | 1.000 | 0.447 | 1.531 |  | 0.000 | -0.936 | 0.840 |
| Famotidine | Brotizolam | 1.643 | 1.332 | 2.071 |  | 1.000 | 0.621 | 1.195 |  | 0.737 | 0.270 | 1.240 |  | -0.431 | -1.252 | 0.328 |
| Famotidine | Phenytoin | 1.643 | 1.332 | 2.071 |  | 2.847 | 1.685 | 4.093 |  | 1.139 | 0.651 | 2.015 |  | 1.838 | -0.285 | 4.574 |
| Famotidine | Fentanyl | 1.643 | 1.332 | 2.071 |  | 4.031 | 3.726 | 8.961 |  | 0.845 | 0.437 | 1.276 |  | 0.924 | -2.633 | 6.122 |
| Famotidine | Remifentanil | 1.643 | 1.332 | 2.071 |  | 1.000 | 0.473 | 1.000 |  | 1.000 | 0.926 | 4.474 |  | 0.000 | -0.259 | 4.012 |
| Famotidine | Midazolam | 1.643 | 1.332 | 2.071 |  | 1.000 | 0.814 | 1.575 |  | 0.878 | 0.341 | 1.118 |  | -0.200 | -1.243 | 0.405 |
| Famotidine | Daikenchuto | 1.643 | 1.332 | 2.071 |  | 1.000 | 0.925 | 1.936 |  | 1.000 | 0.412 | 1.214 |  | 0.000 | -1.135 | 0.503 |
| Vonoprazan | Rebamipide | 1.000 | 0.392 | 1.000 |  | 0.739 | 0.345 | 0.760 |  | 1.000 | 0.241 | 1.292 |  | 0.000 | -0.346 | 0.334 |
| Vonoprazan | Magnesium oxide | 1.000 | 0.392 | 1.000 |  | 0.949 | 0.749 | 1.096 |  | 0.846 | 0.199 | 1.046 |  | -0.146 | -0.700 | 0.095 |
| Misoprostol | Magnesium oxide | 1.000 | 0.330 | 1.000 |  | 0.949 | 0.749 | 1.096 |  | 1.000 | 0.241 | 1.725 |  | 0.000 | -0.644 | 0.393 |
| Ranitidine | Rabeprazole | 1.155 | 1.015 | 2.030 |  | 1.000 | 0.538 | 1.000 |  | 1.000 | 0.052 | 1.117 |  | 0.000 | -1.441 | 0.071 |
| Ranitidine | Rebamipide | 1.155 | 1.015 | 2.030 |  | 0.739 | 0.345 | 0.760 |  | 1.000 | 0.294 | 1.054 |  | -0.040 | -0.970 | -0.024 |
| Ranitidine | Magnesium oxide | 1.155 | 1.015 | 2.030 |  | 0.949 | 0.749 | 1.096 |  | 1.000 | 0.983 | 3.376 |  | -0.008 | -0.118 | 3.127 |
| Ranitidine | Sennoside | 1.155 | 1.015 | 2.030 |  | 1.000 | 0.852 | 1.372 |  | 1.537 | 1.000 | 2.794 |  | 0.621 | -0.029 | 2.417 |
| Ranitidine | Fentanyl | 1.155 | 1.015 | 2.030 |  | 4.031 | 3.726 | 8.961 |  | 1.000 | 0.268 | 1.050 |  | 0.471 | -4.481 | 3.906 |
| Ranitidine | Midazolam | 1.155 | 1.015 | 2.030 |  | 1.000 | 0.814 | 1.575 |  | 1.000 | 0.457 | 1.000 |  | 0.000 | -1.022 | 0.087 |
| Rabeprazole | Rebamipide | 1.000 | 0.538 | 1.000 |  | 0.739 | 0.345 | 0.760 |  | 1.000 | 0.765 | 2.212 |  | 0.000 | -0.054 | 0.547 |
| Rabeprazole | Magnesium oxide | 1.000 | 0.538 | 1.000 |  | 0.949 | 0.749 | 1.096 |  | 1.000 | 0.536 | 1.656 |  | 0.000 | -0.364 | 0.490 |
| Rabeprazole | Senna | 1.000 | 0.538 | 1.000 |  | 0.956 | 0.531 | 1.007 |  | 1.000 | 0.088 | 1.239 |  | 0.000 | -0.743 | 0.216 |
| Rabeprazole | Sennoside | 1.000 | 0.538 | 1.000 |  | 1.000 | 0.852 | 1.372 |  | 1.098 | 0.777 | 2.467 |  | 0.098 | -0.213 | 1.095 |
| Rabeprazole | Etizolam | 1.000 | 0.538 | 1.000 |  | 0.805 | 0.270 | 0.865 |  | 1.000 | 0.548 | 3.758 |  | 0.000 | -0.147 | 1.126 |
| Rabeprazole | Zolpidem | 1.000 | 0.538 | 1.000 |  | 1.000 | 0.767 | 1.233 |  | 1.323 | 0.768 | 3.284 |  | 0.323 | -0.188 | 1.692 |
| Rabeprazole | Brotizolam | 1.000 | 0.538 | 1.000 |  | 1.000 | 0.621 | 1.195 |  | 1.000 | 0.521 | 3.588 |  | 0.000 | -0.374 | 1.769 |
| Rabeprazole | Adenosine triphosphate | 1.000 | 0.538 | 1.000 |  | 0.679 | 0.272 | 1.000 |  | 0.634 | 0.074 | 1.000 |  | -0.249 | -0.736 | 0.170 |
| Lansoprazole | Rebamipide | 1.084 | 1.000 | 1.520 |  | 0.739 | 0.345 | 0.760 |  | 1.000 | 1.000 | 3.368 |  | -0.022 | -0.158 | 1.229 |
| Lansoprazole | Magnesium oxide | 1.084 | 1.000 | 1.520 |  | 0.949 | 0.749 | 1.096 |  | 0.986 | 0.428 | 1.029 |  | -0.019 | -0.740 | 0.028 |
| Lansoprazole | Senna | 1.084 | 1.000 | 1.520 |  | 0.956 | 0.531 | 1.007 |  | 1.000 | 0.507 | 2.050 |  | -0.004 | -0.524 | 0.959 |
| Lansoprazole | Sennoside | 1.084 | 1.000 | 1.520 |  | 1.000 | 0.852 | 1.372 |  | 1.000 | 0.588 | 1.839 |  | 0.000 | -0.552 | 0.970 |
| Lansoprazole | Picosulfate | 1.084 | 1.000 | 1.520 |  | 1.072 | 1.000 | 2.023 |  | 1.149 | 0.807 | 3.600 |  | 0.179 | -0.270 | 4.169 |
| Lansoprazole | Haloperidol | 1.084 | 1.000 | 1.520 |  | 1.000 | 0.447 | 1.024 |  | 1.000 | 0.216 | 1.380 |  | 0.000 | -0.913 | 0.368 |
| Lansoprazole | Etizolam | 1.084 | 1.000 | 1.520 |  | 0.805 | 0.270 | 0.865 |  | 0.718 | 0.134 | 1.000 |  | -0.262 | -0.886 | 0.000 |
| Lansoprazole | Zolpidem | 1.084 | 1.000 | 1.520 |  | 1.000 | 0.767 | 1.233 |  | 1.000 | 0.456 | 1.673 |  | 0.000 | -0.644 | 0.809 |
| Lansoprazole | Brotizolam | 1.084 | 1.000 | 1.520 |  | 1.000 | 0.621 | 1.195 |  | 1.136 | 0.838 | 3.567 |  | 0.148 | -0.194 | 2.582 |
| Lansoprazole | Fentanyl | 1.084 | 1.000 | 1.520 |  | 4.031 | 3.726 | 8.961 |  | 1.000 | 0.631 | 2.607 |  | 0.254 | -1.871 | 12.965 |
| Lansoprazole | Daikenchuto | 1.084 | 1.000 | 1.520 |  | 1.000 | 0.925 | 1.936 |  | 1.000 | 0.412 | 1.652 |  | 0.000 | -0.972 | 0.923 |
| Rebamipide | Magnesium oxide | 0.739 | 0.345 | 0.760 |  | 0.949 | 0.749 | 1.096 |  | 1.145 | 0.975 | 2.538 |  | 0.115 | -0.020 | 0.736 |
| Rebamipide | Senna | 0.739 | 0.345 | 0.760 |  | 0.956 | 0.531 | 1.007 |  | 0.765 | 0.130 | 1.192 |  | -0.155 | -0.454 | 0.239 |
| Rebamipide | Sennoside | 0.739 | 0.345 | 0.760 |  | 1.000 | 0.852 | 1.372 |  | 1.000 | 0.574 | 2.335 |  | 0.000 | -0.303 | 0.674 |
| Rebamipide | Haloperidol | 0.739 | 0.345 | 0.760 |  | 1.000 | 0.447 | 1.024 |  | 1.000 | 1.000 | 3.643 |  | 0.000 | -0.012 | 1.145 |
| Rebamipide | Etizolam | 0.739 | 0.345 | 0.760 |  | 0.805 | 0.270 | 0.865 |  | 1.000 | 0.536 | 2.873 |  | 0.051 | 0.002 | 0.709 |

**Supplementary Table S7. Continued.**

| Drug 1 | Drug 2 | Adjusted odds ratio | | | | | | | | | | |  | Synergistic interaction | | |
| --- | --- | --- | --- | --- | --- | --- | --- | --- | --- | --- | --- | --- | --- | --- | --- | --- |
|  |  | Drug 1 | | |  | Drug 2 | | |  | Product term (Drug 1*Drug 2) | | |  |  |  |  |
|  |  | Estimate | 95%BootCI | |  | Estimate | 95%BootCI | |  | Estimate | 95%BootCI | |  | RERI | 95%BootCI | |
|  |  |  | Lower | Upper |  |  | Lower | Upper |  |  | Lower | Upper |  |  | Lower | Upper |
| Rebamipide | Zolpidem | 0.739 | 0.345 | 0.760 |  | 1.000 | 0.767 | 1.233 |  | 1.000 | 0.855 | 3.229 |  | 0.000 | -0.112 | 1.126 |
| Rebamipide | Brotizolam | 0.739 | 0.345 | 0.760 |  | 1.000 | 0.621 | 1.195 |  | 1.000 | 0.391 | 1.790 |  | 0.000 | -0.338 | 0.402 |
| Rebamipide | Fentanyl | 0.739 | 0.345 | 0.760 |  | 4.031 | 3.726 | 8.961 |  | 0.957 | 0.427 | 2.051 |  | -0.919 | -5.222 | 0.865 |
| Rebamipide | Remifentanil | 0.739 | 0.345 | 0.760 |  | 1.000 | 0.473 | 1.000 |  | 1.000 | 0.318 | 2.534 |  | 0.000 | -0.296 | 0.706 |
| Rebamipide | Midazolam | 0.739 | 0.345 | 0.760 |  | 1.000 | 0.814 | 1.575 |  | 0.764 | 0.262 | 1.000 |  | -0.175 | -0.624 | 0.109 |
| Magnesium oxide | Dimeticone | 0.949 | 0.749 | 1.096 |  | 1.000 | 0.476 | 1.062 |  | 1.000 | 0.647 | 2.797 |  | 0.000 | -0.296 | 1.111 |
| Magnesium oxide | Senna | 0.949 | 0.749 | 1.096 |  | 0.956 | 0.531 | 1.007 |  | 1.000 | 0.464 | 1.257 |  | 0.002 | -0.494 | 0.195 |
| Magnesium oxide | Sennoside | 0.949 | 0.749 | 1.096 |  | 1.000 | 0.852 | 1.372 |  | 0.811 | 0.336 | 1.000 |  | -0.179 | -0.766 | 0.000 |
| Magnesium oxide | Picosulfate | 0.949 | 0.749 | 1.096 |  | 1.072 | 1.000 | 2.023 |  | 1.000 | 0.736 | 2.028 |  | -0.004 | -0.407 | 1.190 |
| Magnesium oxide | Haloperidol | 0.949 | 0.749 | 1.096 |  | 1.000 | 0.447 | 1.024 |  | 1.000 | 0.538 | 3.026 |  | 0.000 | -0.402 | 1.498 |
| Magnesium oxide | Risperidone | 0.949 | 0.749 | 1.096 |  | 0.916 | 0.620 | 1.437 |  | 0.858 | 0.098 | 1.013 |  | -0.119 | -1.043 | 0.049 |
| Magnesium oxide | Etizolam | 0.949 | 0.749 | 1.096 |  | 0.805 | 0.270 | 0.865 |  | 1.000 | 0.627 | 2.954 |  | 0.010 | -0.182 | 0.902 |
| Magnesium oxide | Zolpidem | 0.949 | 0.749 | 1.096 |  | 1.000 | 0.767 | 1.233 |  | 1.000 | 0.637 | 1.915 |  | 0.000 | -0.354 | 0.842 |
| Magnesium oxide | Brotizolam | 0.949 | 0.749 | 1.096 |  | 1.000 | 0.621 | 1.195 |  | 1.000 | 0.348 | 1.351 |  | 0.000 | -0.611 | 0.302 |
| Magnesium oxide | Fentanyl | 0.949 | 0.749 | 1.096 |  | 4.031 | 3.726 | 8.961 |  | 1.056 | 1.000 | 5.446 |  | 0.061 | -0.483 | 25.686 |
| Magnesium oxide | Daikenchuto | 0.949 | 0.749 | 1.096 |  | 1.000 | 0.925 | 1.936 |  | 1.000 | 0.425 | 1.131 |  | 0.000 | -0.912 | 0.142 |
| Dimeticone | Sennoside | 1.000 | 0.476 | 1.062 |  | 1.000 | 0.852 | 1.372 |  | 1.000 | 0.338 | 1.323 |  | 0.000 | -0.706 | 0.188 |
| Senna | Sennoside | 0.956 | 0.531 | 1.007 |  | 1.000 | 0.852 | 1.372 |  | 1.000 | 0.229 | 1.729 |  | 0.000 | -0.773 | 0.620 |
| Senna | Picosulfate | 0.956 | 0.531 | 1.007 |  | 1.072 | 1.000 | 2.023 |  | 0.940 | 0.372 | 2.116 |  | -0.065 | -0.945 | 1.064 |
| Senna | Fentanyl | 0.956 | 0.531 | 1.007 |  | 4.031 | 3.726 | 8.961 |  | 1.644 | 1.000 | 7.031 |  | 2.348 | -1.475 | 29.626 |
| Senna | Daikenchuto | 0.956 | 0.531 | 1.007 |  | 1.000 | 0.925 | 1.936 |  | 1.000 | 0.674 | 2.378 |  | 0.000 | -0.453 | 1.286 |
| Sennoside | Picosulfate | 1.000 | 0.852 | 1.372 |  | 1.072 | 1.000 | 2.023 |  | 0.916 | 0.277 | 1.290 |  | -0.090 | -1.172 | 0.384 |
| Sennoside | Etizolam | 1.000 | 0.852 | 1.372 |  | 0.805 | 0.270 | 0.865 |  | 1.000 | 0.370 | 2.508 |  | 0.000 | -0.466 | 0.664 |
| Sennoside | Zolpidem | 1.000 | 0.852 | 1.372 |  | 1.000 | 0.767 | 1.233 |  | 1.000 | 0.488 | 1.835 |  | 0.000 | -0.577 | 0.810 |
| Sennoside | Brotizolam | 1.000 | 0.852 | 1.372 |  | 1.000 | 0.621 | 1.195 |  | 1.000 | 0.651 | 2.717 |  | 0.000 | -0.362 | 1.585 |
| Sennoside | Fentanyl | 1.000 | 0.852 | 1.372 |  | 4.031 | 3.726 | 8.961 |  | 1.000 | 0.872 | 2.993 |  | 0.000 | -0.896 | 12.259 |
| Sennoside | Daikenchuto | 1.000 | 0.852 | 1.372 |  | 1.000 | 0.925 | 1.936 |  | 1.435 | 0.804 | 3.255 |  | 0.435 | -0.237 | 2.799 |
| Picosulfate | Zolpidem | 1.072 | 1.000 | 2.023 |  | 1.000 | 0.767 | 1.233 |  | 1.000 | 0.231 | 1.365 |  | 0.000 | -1.258 | 0.469 |
| Picosulfate | Daikenchuto | 1.072 | 1.000 | 2.023 |  | 1.000 | 0.925 | 1.936 |  | 1.000 | 0.243 | 1.655 |  | 0.000 | -1.400 | 1.055 |
| Haloperidol | Fentanyl | 1.000 | 0.447 | 1.024 |  | 4.031 | 3.726 | 8.961 |  | 1.000 | 0.460 | 3.756 |  | 0.000 | -3.883 | 13.735 |
| Etizolam | Zolpidem | 0.805 | 0.270 | 0.865 |  | 1.000 | 0.767 | 1.233 |  | 1.000 | 0.745 | 4.597 |  | 0.000 | -0.167 | 1.563 |
| Etizolam | Brotizolam | 0.805 | 0.270 | 0.865 |  | 1.000 | 0.621 | 1.195 |  | 1.000 | 0.234 | 1.630 |  | 0.000 | -0.518 | 0.301 |
| Adenosine triphosphate | Betahistine | 0.679 | 0.272 | 1.000 |  | 1.000 | 0.418 | 1.357 |  | 1.000 | 0.111 | 1.000 |  | 0.000 | -0.919 | 0.253 |
| Benserazide | Levodopa | 0.769 | 0.107 | 1.000 |  | 0.632 | 0.139 | 1.000 |  | 1.000 | 0.954 | 1.000 |  | 0.085 | 0.000 | 0.530 |
| Fentanyl | Remifentanil | 4.031 | 3.726 | 8.961 |  | 1.000 | 0.473 | 1.000 |  | 1.000 | 1.000 | 1.264 |  | 0.000 | -3.639 | 1.233 |
| Fentanyl | Midazolam | 4.031 | 3.726 | 8.961 |  | 1.000 | 0.814 | 1.575 |  | 1.636 | 1.000 | 3.821 |  | 2.564 | 0.000 | 18.489 |
| Fentanyl | Daikenchuto | 4.031 | 3.726 | 8.961 |  | 1.000 | 0.925 | 1.936 |  | 1.158 | 0.528 | 2.693 |  | 0.638 | -2.476 | 15.482 |
| Buprenorphine | Pentazocine | 1.000 | 0.998 | 7.643 |  | 1.000 | 0.564 | 1.922 |  | 1.000 | 0.016 | 1.000 |  | 0.000 | -5.616 | 0.282 |

Relative excess risk due to interaction (RERI), which is a synergistic interaction measure, was calculated using adjusted odds ratios for Drug 1, Drug 2, and corresponding product term. Abbreviations: 95%BootCI, 95% bootstrap percentile confidence interval; RERI, relative excess risk due to interaction.
